# Supplementary material for: Proteomic, Transcriptomic, Mutational, and Functional Assays Reveal the Involvement of Both THF and PLP Sites at the GmSHMT08 in Resistance to Soybean Cyst Nematode
Source: Int J Mol Sci. 2022 Sep 24;23(19):11278. doi: 10.3390/ijms231911278 (PMC9570156; doi:10.3390/ijms231911278)
Supplement: Supplementary file 1 [file ijms-23-11278-s001.zip › Supplemental Figures S1-S11.pdf]

Proteomic, Transcriptomic, Mutational, and Functional Assays Reveal the Involvement of Both THF and PLP Sites at the GmSHMT08 in Resistance to Soybean Cyst Nematode

Naoufal Lakhssassi [1], Dounya Knizia [1], Abdelhalim El Baze [1], Aicha Lakhssassi [2], Jonas Meksem [3] and Khalid Meksem [1],*

1 Department of Plant, Soil and Agricultural Systems, Southern Illinois University, Carbondale, IL 62901, USA
2 Faculté des Sciences et Techniques, Université Abdelmalek Essaâdi, Tanger 90000, Morocco
3 Trinity College of Arts and Sciences, Duke University, Durham, NC 27708, USA; jonas.meksem@duke.edu
* Correspondence: meksem@siu.edu

Supplemental material

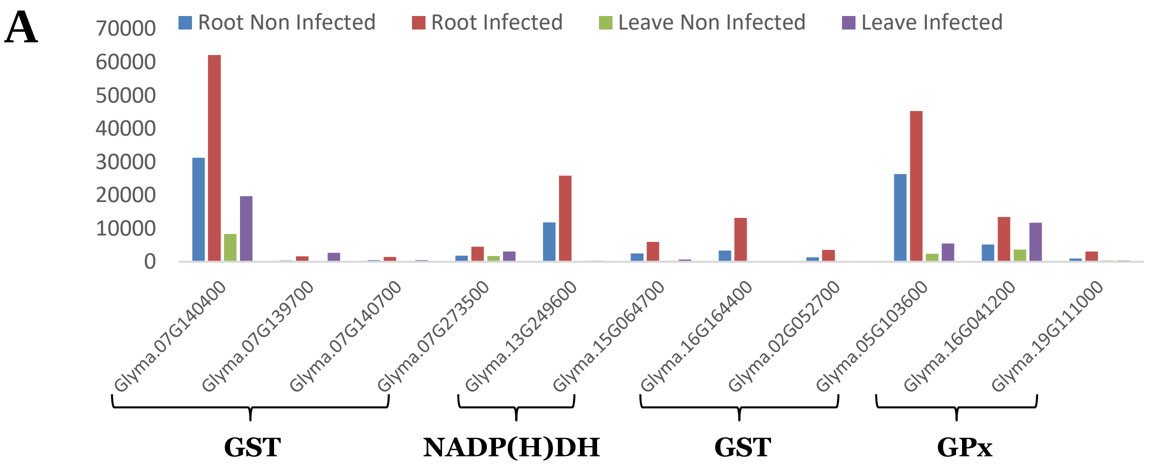

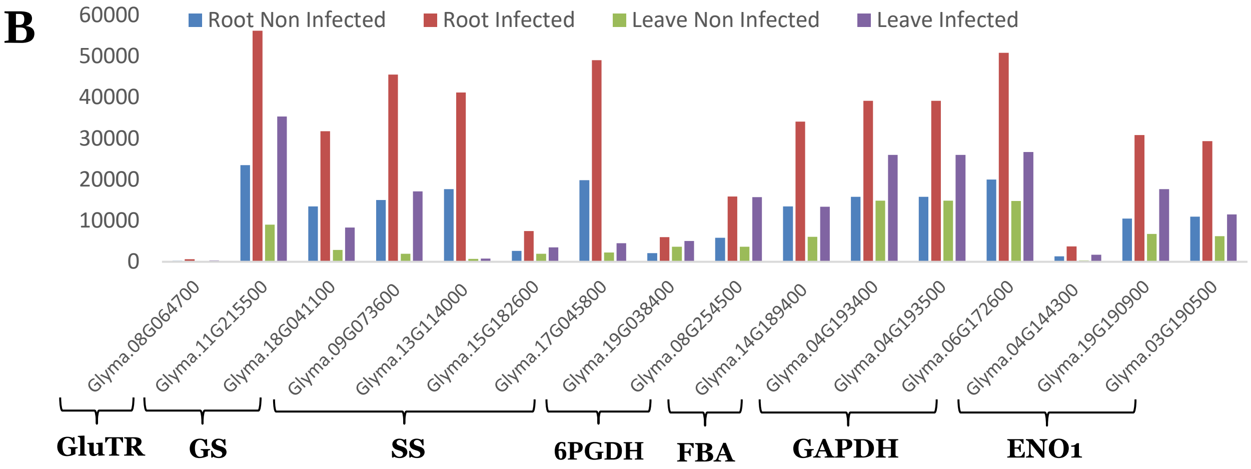

Supplemental Figure S1. Expression analysis of SCN infected (3DAI) and non-infected soybean roots and leaves based on RNAseq data analysis of all the identified genes from mass spectrometry shown in Table S2, corresponding to key components related to redox hemeostasis **(A)**, Glycolysis **(B)**, Glyoxylate cycle **(C)**, Succinyl-CoA and heme biosynthesis related enzymes **(D),** Cytoskeleton-related genes **(E)**, and ATP mitochondrial related genes **(F)**.

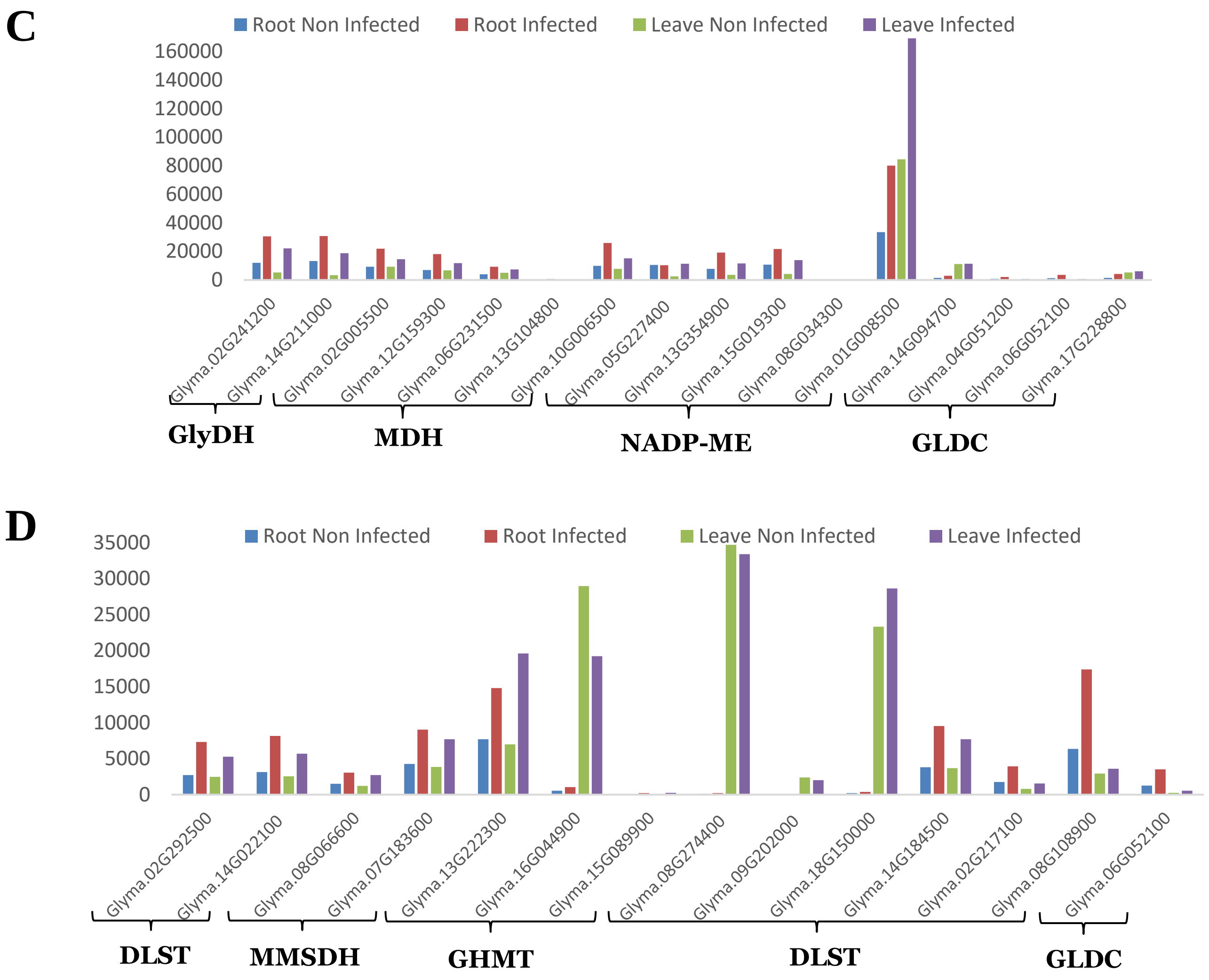

Supplemental Figure S1. Expression analysis of SCN infected (3DAI) and non-infected soybean roots and leaves based on RNAseq data analysis of all the identified genes from mass spectrometry shown in Table S2, corresponding to key components related to redox hemeostasis **(A)**, Glycolysis **(B)**, Glyoxylate cycle **(C)**, Succinyl-CoA and heme biosynthesis related enzymes **(D),** Cytoskeleton-related genes **(E)**, and ATP mitochondrial related genes **(F)**.

E

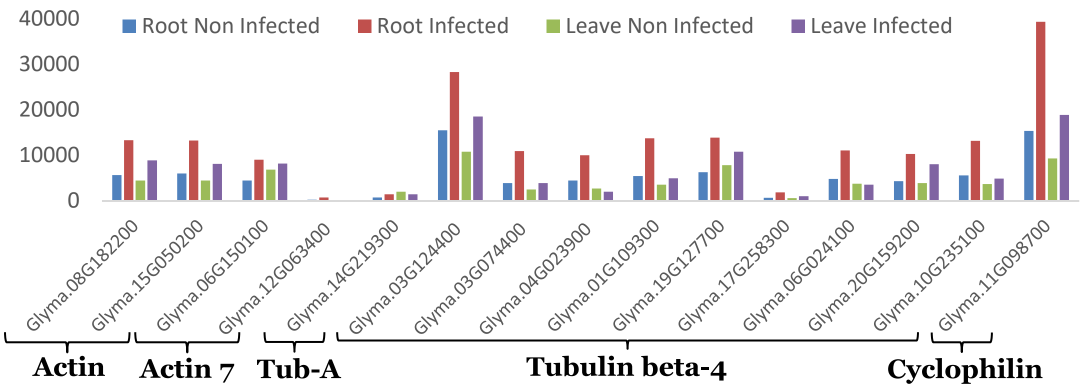

F

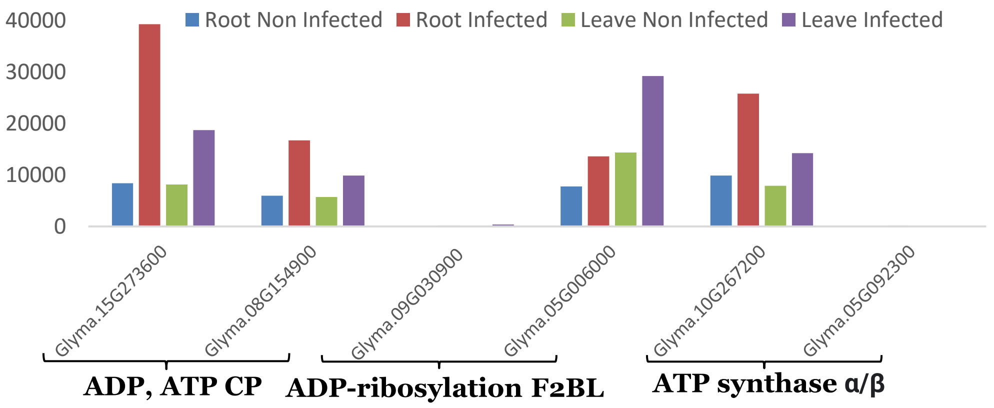

Supplemental Figure S1. Expression analysis of SCN infected (3DAI) and non-infected soybean roots and leaves based on RNAseq data analysis of all the identified genes from mass spectrometry shown in Table S2, corresponding to key components related to redox hemeostasis **(A)**, Glycolysis **(B)**, Glyoxylate cycle **(C)**, Succinyl-CoA and heme biosynthesis related enzymes **(D),** Cytoskeleton-related genes **(E)**, and ATP mitochondrial related genes **(F)**.

Gene Name F-SHMT08c-CDS
Cloning Vector pG2RNAi2
Length (bp) 1430
Cloning Sites AscI-AvrII

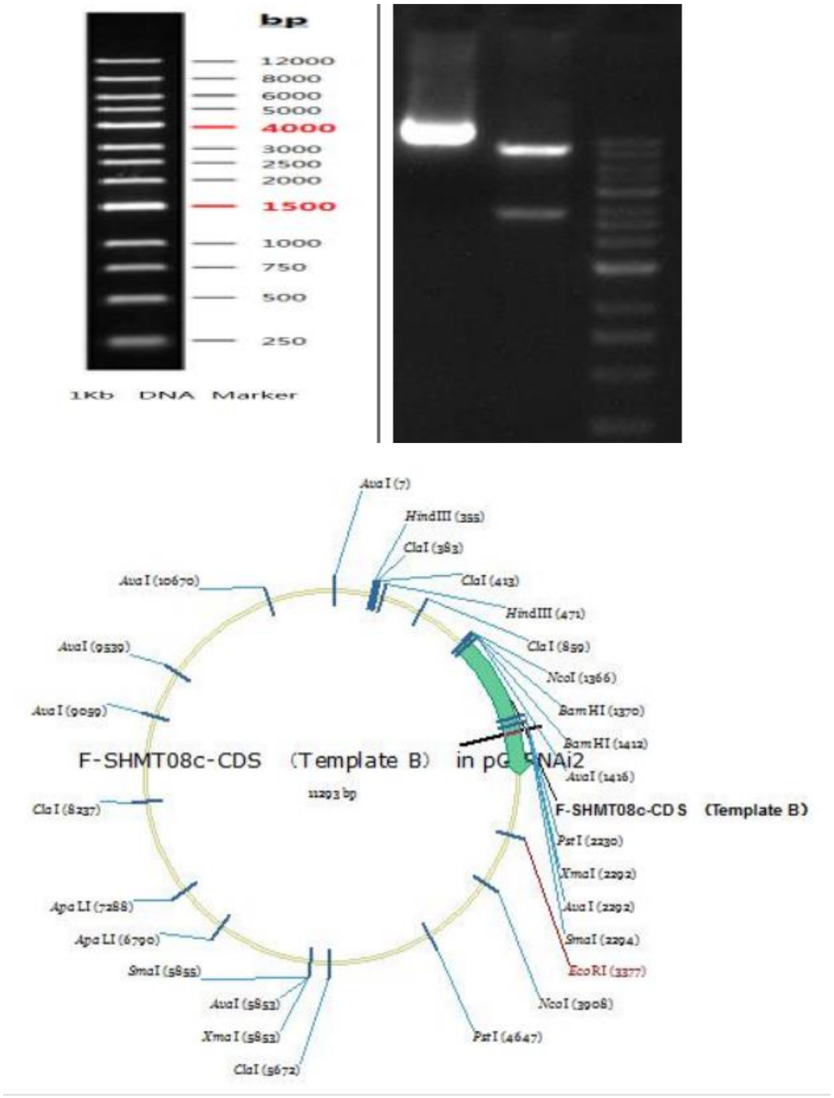

Supplemental Figure S2. F-SHMT08c-CDS, insert size, vector, restriction enzymes, and sequenced positive clones used for cloning.

Detailed Sequence of the Whole Construct:

```
   1 TTGATCCCGA GGGGAACCCT GTGGTTGGCA TGCACATACA AATGGACGAA CGGATAAACC
  61 TTTTCACGCC CTTTTAAATA TCCGTTATTC TAATAAACGC TCTTTTCTCT TAGGTTTACC
 121 CGCCAATATA TCCTGTCAAA CACTGATAGT TTAAACTGAA GGCGGGAAAC GACAATCTGA
 181 TCCAAGCTCA AGCTCATTCG CCATTCAGGC TGCGCAACTG TTGGGAAGGG CGATCGGTGC
 241 GGGCCTCTTC GCTATTACGC CAGCTGGCGA AAGGGGGATG TGCTGCAAGG CGATTAAGTT
 301 GGGTAACGCC AGGGTTTTCC CAGTCACGAC GTTGTAAAAC GACGGCCAGT GCCAAGCTTA
 361 TTACCCTGTT ATCCCTAGAT ATCGATTTTG GCTACCTTAA GGTAGCCAAA ATCGATCACT
 421 AGTGCGGCCG CGGGCCCAAT ATAACAACGA CGTCGTAACA GATAAAGCGA AGCTTGAAGG
 481 TGCATGTGAC TCCGTCAAGA TTACGAAACC GCCAACTACC ACGCAAATTG CAATTCTCAA
 541 TTTCCTAGAA GGACTCTCCG AAAATGCATC CAATACCAAA TATTACCCGT GTCATAGGCA
 601 CCAAGTGACA CCATACATGA ACACGCGTCA CAATATGACT GGAGAAGGGT TCCACACCTT
 661 ATGCTATAAA ACGCCCCACA CCCCTCCTCC TTCCTTCGCA GTTCAATTCC AATATATTCC
 721 ATTCTCTCTG TGTATTTCCC TACCTCTCCC TTCAAGGTTA GTCGATTTCT TCTGTTTTTC
 781 TTCTTCGTTC TTTCCATGAA TTGTGTATGT TCTTTGATCA ATACGATGTT GATTTGATTG
 841 TGTTTTGTTT GGTTTCATCG ATCTTCAATT TTCATAATCA GATTCAGCTT TTATTATCTT
 901 TACAACAACG TCCTTAATTT GATGATTCTT TAATCGTAGA TTTGCTCTAA TTAGAGCTTT
 961 TTCATGTCAG ATCCCTTTAC AACAAGCCTT AATTGTTGAT TCATTAATCG TAGATTAGGG
1021 CTTTTTTCAT TGATTACTTC AGATCCGTTA AACGTAACCA TAGATCAGGG CTTTTTCATG
1081 AATTACTTCA GATCCGTTAA ACAACAGCCT TATTTTTTAT ACTTCTGTGG TTTTTCAAGA
1141 AATTGTTCAG ATCCGTTGAC AAAAAGCCTT ATTCGTTGAT TCTATATCGT TTTTCGAGAG
1201 ATATTGCTCA GATCTGTTAG CAACTGCCTT GTTTGTTGAT TCTATTGCCG TGGATTAGGG
1261 TTTTTTTTCA CGAGATTGCT TCAGATCCGT ACTTAAGATT ACGTAATGGA TTTTGATTCT
1321 GATTTATCTG TGATTGTTGA CTCGACAGCG GCCGCACCGG CGCGCCATGG ATCCAGTAAG
1381 CGTGTGGGGT AACACGCCCT TGGCGACGGT GGATCCCGAG ATCCATGACC TCATCGAGAA
1441 GGAGAAGCGC CGTCAATGCC GCGGAATCGA GCTCATCGCC TCCGAGAACT TCACCTCCTT
1501 CGCCGTCATC GAGGCCCTCG GCAGCGCTCT CACGAACAAA TACTCCGAGG GCATGCCGGG
```

Supplemental Figure S2. F-SHMT08c-CDS, insert size, vector, restriction enzymes, and sequenced positive clones used for cloning.

```
1561 CAACCGCTAC TACGGCGGCA ATGAATACAT CGACCAGATC GAAAACCTCT GCCGCTCACG
1621 CGCCCTCCAA GCCTTCCACC TCGACGCCCA ATCCTGGGGC GTCAACGTCC AGCCCTACTC
1681 CGGCTCCCCG GCCAACTTCG CCGCCTACAC CGCCGTCCTC AACCCCCACG ACCGCATCAT
1741 GGGGCTAGAT CTCCGCTCCG GCGGCCACCT CACCCACGGC TACTACACCT CCGGCGGAAA
1801 GAAGATCTCC GCCACCTCCA TTTACTTCGA GAGTCTCCCT TACAAGGTAA ACTCCACCAC
1861 CGGCTACATC GACTACGACC GCTTGGAAGA AAAAGCCCTA GACTTCAGGC CAAAACTCAT
1921 AATCTGCGGT GGCAGCGCGT ACCCTCGCGA TTGGGACTAC AAACGTTTCA GGGAAGTCGC
1981 TGATAAGTGC GGAGCATTGC TTCTCTGCGA CATGGCGCAC ACTAGCGGCC TTGTGGCCGC
2041 GCAGGAAGTG AACAGCCCCT TCGAGTATTG CGACATTGTG ACCACCACGA CTCACAAGAG
2101 CTTGCGGGGC CCACGTGCGG GGATGATCTT TTACCGGAAG GGCCCCAAGC CGCCGAAGAA
2161 GGGGCAGCCG GAGAACGCGG TTTATGATTT CGAGGACAAG ATTAACTTCG CGGTGTTCCC
2221 TTCGCTGCAG GGTGGGCCCC ACAACCACCA GATCGGTGCT CTCGCCGTGG CGCTGAAGCA
2281 GGCCGCGTCG CCCGGGTTTA AGGCCTACGC GAAGCAGGTT AAGGCGAACG CCGTTGCGCT
2341 TGGAAAATAC TTGATGGGGA AAGGGTACAG CCTTGTCACT GGCGGAACGG AGAACCATCT
2401 TGTTTTGTGG GATCTGAGAC CTCTTGGATT GACTGGGTAT AAGGTGGAGA AACTCTGTGA
2461 TCTCTGTAAC ATTACTGTTA ACAAGAACGC TGTTTTTGGT GATAGCAGTG CCTTGGCCCC
2521 TGGTGGAGTG CGAATTGGTG CCCCTGCCAT GACTTCTAGG GGTTTGGTTG AAAAAGACTT
2581 TGAGCAGATT GGTGAGTTCC TTCACCGTGC TGTGACTCTC ACACTGGAGA TCCAGAAGGA
2641 GCATGGCAAA CTTCTCAAGG ATTTCAACAA GGGTCTCGTC AACAACAAGG CTATTGAAGA
2701 TCTCAAAGCT GATGTTGAGA AGTTCTCTGC CTTGTTTGAC ATGCCTGGCT TCCTGGTATC
2761 TGAAATGAAG TACAAGGATT AGCCTAGGTT CGAGTATTAT GGCATTGGGA AAACTGTTTT
2821 TCTTGTACCA TTTGTTGTGC TTGTAATTTA CTGTGTTTTT TATTCGGTTT TCGCTATCGA
2881 ACTGTGAAAT GGAAATGGAT GGAGAAGAGT TAATGAATGA TATGGTCCTT TTGTTCATTC
2941 TCAAATTAAT ATTATTTGTT TTTTCTCTTA TTTGTTGTGT GTTGAATTTG AAATTATAAG
3001 AGATATGCAA ACATTTTGTT TTGAGTAAAA ATGTGTCAAA TCGTGGCCTC TAATGACCGA
3061 AGTTAATATG AGGAGTAAAA CACTTGTAGT TGTACCATTA TGCTTATTCA CTAGGCAACA
3121 AATATATTTT CAGACCTAGA AAAGCTGCAA ATGTTACTGA ATACAAGTAT GTCCTCTTGT
3181 GTTTTAGACA TTTATGAACT TTCCTTTATG TAATTTTCCA GAATCCTTGT CAGATTCTAA
3241 TCATTGCTTT ATAATTATAG TTATACTCAT GGATTTGTAG TTGAGTATGA AAATATTTTT
3301 TAATGCATTT TATGACTTGC CAATTGATTG ACAACATGCA TCAATCCGCG GTTATGACTC
3361 TCTTAAGAGA GTCATGAATT CGAGCTTCCA GAAGGTAATT ATCCAAGATG TAGCATCAAG
3421 AATCCAATGT TTACGGGAAA AACTATGGAA GTATTATGTG AGCTCAGCAA GAAGCAGATC
3481 AATATGCGGC ACATATGCAA CCTATGTTCA AAAATGAAGA ATGTACAGAT ACAAGATCCT
3541 ATACTGCCAG AATACGAAGA AGAATACGTA GAAATTGAAA AAGAAGAACC AGGCGAAGAA
3601 AAGAATCTTG AAGACGTAAG CACTGACGAC AACAATGAAA AGAAGAAGAT AAGGTCGGTG
3661 ATTGTGAAAG AGACATAGAG GACACATGTA AGGTGGAAAA TGTAAGGGCG GAAAGTAACC
3721 TTATCACAAA GGAATCTTAT CCCCCACTAC TTATCCTTTT ATATTTTTCC GTGTCATTTT
3781 TGCCCTTGAG TTTTCCTATA TAAGGAACCA AGTTCGGCAT TTGTGAAAAC AAGAAAAAAT
3841 TTGGTGTAAG CTATTTTCTT TGAAGTACTG AGGATACAAC TTCAGAGAAA TTTGTAAGTT
3901 TGTGATCCAT GGTGAGCAAG GGCGAGGAGC TGTTCACCGG GGTGGTGCCC ATCCTGGTCG
3961 AGCTGGACGG CGACGTAAAC GGCCACAAGT TCAGCGTGTC CGGCGAGGGC GAGGGCGATG
4021 CCACCTACGG CAAGCTGACC CTGAAGTTCA TCTGCACCAC CGGCAAGCTG CCCGTGCCCT
4081 GGCCCACCCT CGTGACCACC TTCACCTACG GCGTGCAGTG CTTCAGCCGC TACCCCGACC
4141 ACATGAAGCA GCACGACTTC TTCAAGTCCG CCATGCCCGA AGGCTACGTC CAGGAGCGCA
4201 CCATCTTCTT CAAGGACGAC GGCAACTACA AGACCCGCGC CGAGGTGAAG TTCGAGGGCG
4261 ACACCCTGGT GAACCGCATC GAGCTGAAGG GCATCGACTT CAAGGAGGAC GGCAACATCC
4321 TGGGGCACAA GCTGGAGTAC AACTACAACA GCCACAACGT CTATATCATG GCCGACAAGC
4381 AGAAGAACGG CATCAAGGTG AACTTCAAGA TCCGCCACAA CATCGAGGAC GGCAGCGTGC
4441 AGCTCGCCGA CCACTACCAG CAGAACACCC CCATCGGCGA CGGCCCCGTG CTGCTGCCCG
4501 ACAACCACTA CCTGAGCACC CAGTCCGCCC TGAGCAAAGA CCCCAACGAG AAGCGCGATC
4561 ACATGGTCCT GCTGGAGTTC GTGACCGCCG CCGGGATCAC TCACGGCATG GACGAGCTGT
4621 ACAAGTAAAG CGGCCGCCCG GCTGCAGATC GTTCAAACAT TTGGCAATAA AGTTTCTTAA
4681 GATTGAATCC TGTTGCCGGT CTTGCGATGA TTATCATATA ATTTCTGTTG AATTACGTTA
4741 AGCATGTAAT AATTAACATG TAATGCATGA CGTTATTTAT GAGATGGGTT TTTATGATTA
```

Supplemental Figure S2. F-SHMT08c-CDS, insert size, vector, restriction enzymes, and sequenced positive clones used for cloning.

```
4801 GAGTCCCGCA ATTATACATT TAATACGCGA TAGAAAACAA AATATAGCGC GCAAACTAGG
4861 ATAAATTATC GCGCGCGGTG TCATCTATGT TACTAGATCC GATGATAAGC TGTCAAACAT
4921 GAGAATTAAT TCGTAATCAT GTCATAGCTG TTTCCTGTGT GAAATTGTTA TCCGCTCACA
4981 ATTCCACACA ACATACGAGC CGGAAGCATA AAGTGTAAAG CCTGGGGTGC CTAATGAGTG
5041 AGCTAACTCA CATTAATTGC GTTGCGCTCA CTGCCCGCTT TCCAGTCGGG AAACCTGTCG
5101 TGCCAGCTGC ATTAATGAAT CGGCCAACGC GCGGGGAGAG GCGGTTTGCG TATTGGCTAG
5161 AGCAATTCGG CGTTAATTCA GTACATTAAA AACGTCCGCA ATGTGTTATT AAGTTGTCTA
5221 AGCGTCAATT TGTTTACACC ACAATATATC CTGCCACCAG CCAGCCAACA GCTCCCCGAC
5281 CGGCAGCTCG GCACAAAATC ACCACTCGAT ACAGGCAGCC CATCAGTCCG GGACGGCGTC
5341 AGCGGGAGAG CCGTTGTAAG GCGGCAGACT TTGCTCATGT TACCGATGCT ATTCGGAAGA
5401 ACGGCAACTA AGCTGCCGGG TTTGAAACAC GGATGATCTC GCGGAGGGTA GCATGTTGAT
5461 TGTAACGATG ACAGAGCGTT GCTGCCTGTG ATCCAGATCA TGAACAATAA AACTGTCTGC
5521 TTACATAAAC AGTAATACAA GGGGTGTTAT GAGCCATATT CAACGGGAAA CGTCTTGCTC
5581 TAGGCCGCGA TTAAATTCCA ACATGGATGC TGATTTATAT GGGTATAAAT GGGCTCGCGA
5641 TAATGTCGGG CAATCAGGTG CGACAATCTA TCGATTGTAT GGGAAGCCCG ATGCGCCAGA
5701 GTTGTTTCTG AAACATGGCA AAGGTAGCGT TGCCAATGAT GTTACAGATG AGATGGTCAG
5761 ACTAAACTGG CTGACGGAAT TTATGCCTCT TCCGACCATC AAGCATTTTA TCCGTACTCC
5821 TGATGATGCA TGGTTACTCA CCACTGCGAT CCCCGGGAAA ACAGCATTCC AGGTATTAGA
5881 AGAATATCCT GATTCAGGTG AAAATATTGT TGATGCGCTG GCAGTGTTCC TGCGCCGGTT
5941 GCATTCGATT CCTGTTTGTA ATTGTCCTTT TAACAGCGAT CGCGTATTTC GTCTCGCTCA
6001 GGCGCAATCA CGAATGAATA ACGGTTTGGT TGATGCGAGT GATTTTGATG ACGAGCGTAA
6061 TGGCTGGCCT GTTGAACAAG TCTGGAAAGA AATGCATAAA CTTTTGCCAT TCTCACCGGA
6121 TTCAGTCGTC ACTCATGGTG ATTTCTCACT TGATAACCTT ATTTTTGACG AGGGGAAATT
6181 AATAGGTTGT ATTGATGTTG GACGAGTCGG AATCGCAGAC CGATACCAGG ATCTTGCCAT
6241 CCTATGGAAC TGCCTCGGTG AGTTTTCTCC TTCATTACAG AAACGGCTTT TTCAAAAATA
6301 TGGTATTGAT AATCCTGATA TGAATAAATT GCAGTTTCAT TTGATGCTCG ATGAGTTTTT
6361 CTAAGAATTA ATTCATGATC CTGCATGACC AAAATCCCTT AACGTGAGTT TTCGTTCCAC
6421 TGAGCGTCAG ACCCCGTAGA AAAGATCAAA GGATCTTCTT GAGATCCTTT TTTTCTGCGC
6481 GTAATCTGCT GCTTGCAAAC AAAAAAACCA CCGCTACCAG CGGTGGTTTG TTTGCCGGAT
6541 CAAGAGCTAC CAACTCTTTT TCCGAAGGTA ACTGGCTTCA GCAGAGCGCA GATACCAAAT
6601 ACTGTCCTTC TAGTGTAGCC GTAGTTAGGC CACCACTTCA AGAACTCTGT AGCACCGCCT
6661 ACATACCTCG CTCTGCTAAT CCTGTTACCA GTGGCTGCTG CCAGTGGCGA TAAGTCGTGT
6721 CTTACCGGGT TGGACTCAAG ACGATAGTTA CCGGATAAGG CGCAGCGGTC GGGCTGAACG
6781 GGGGGTTCGT GCACACAGCC CAGCTTGGAG CGAACGACCT ACACCGAACT GAGATACCTA
6841 CAGCGTGAGC TATGAGAAAG CGCCACGCTT CCCGAAGGGA GAAAGGCGGA CAGGTATCCG
6901 GTAAGCGGCA GGGTCGGAAC AGGAGAGCGC ACGAGGGAGC TTCCAGGGGG AAACGCCTGG
6961 TATCTTTATA GTCCTGTCGG GTTTCGCCAC CTCTGACTTG AGCGTCGATT TTTGTGATGC
7021 TCGTCAGGGG GGCGGAGCCT ATGGAAAAAC GCCAGCAACG CGGCCTTTTT ACGGTTCCTG
7081 GCCTTTTGCT GGCCTTTTGC TCACATGTTC TTTCCTGCGT TATCCCCTGA TTCTGTGGAT
7141 AACCGTATTA CCGCCTTTGA GTGAGCTGAT ACCGCTCGCC GCAGCCGAAC GACCGAGCGC
7201 AGCGAGTCAG TGAGCGAGGA AGCGGAAGAG CGCCTGATGC GGTATTTTCT CCTTACGCAT
7261 CTGTGCGGTA TTTCACACCG CATATGGTGC ACTCTCAGTA CAATCTGCTC TGATGCCGCA
7321 TAGTTAAGCC AGTATACACT CCGCTATCGC TACGTGACTG GGTCATGGCT GCGCCCCGAC
7381 ACCCGCCAAC ACCCGCTGAC GCGCCCTGAC GGGCTTGTCT GCTCCCGGCA TCCGCTTACA
7441 GACAAGCTGT GACCGTCTCC GGGAGCTGCA TGTGTCAGAG GTTTTCACCG TCATCACCGA
7501 AACGCGCGAG GCAGGGTGCC TTGATGTGGG CGCCGGCGGT CGAGTGGCGA CGGCGCGGCT
7561 TGTCCGCGCC CTGGTAGATT GCCTGGCCGT AGGCCAGCCA TTTTTGAGCG GCCAGCGGCC
7621 GCGATAGGCC GACGCGAAGC GGCGGGGCGT AGGGAGCGCA GCGACCGAAG GGTAGGCGCT
7681 TTTTGCAGCT CTTCGGCTGT GCGCTGGCCA GACAGTTATG CACAGGCCAG GCGGGTTTTA
7741 AGAGTTTTAA TAAGTTTTAA AGAGTTTTAG GCGGAAAAAT CGCCTTTTTT CTCTTTTATA
7801 TCAGTCACTT ACATGTGTGA CCGGTTCCCA ATGTACGGCT TTGGGTTCCC AATGTACGGG
7861 TTCCGGTTCC CAATGTACGG CTTTGGGTTC CCAATGTACG TGCTATCCAC AGGAAAGAGA
7921 CCTTTTCGAC CTTTTTCCCC TGCTAGGGCA ATTTGCCCTA GCATCTGCTC CGTACATTAG
7981 GAACCGGCGG ATGCTTCGCC CTCGATCAGG TTGCGGTAGC GCATGACTAG GATCGGGCCA
```

```
8041 GCCTGCCCCG CCTCCTCCTT CAAATCGTAC TCCGGCAGGT CATTTGACCC GATCAGCTTG
8101 CGCACGGTGA AACAGAACTT CTTGAACTCT CCGGCGCTGC CACTGCGTTC GTAGATCGTC
8161 TTGAACAACC ATCTGGCTTC TGCCTTGCCT GCGGCGCGGC GTGCCAGGCG GTAGAGAAAA
8221 CGGCCGATGC CGGGATCGAT CAAAAAGTAA TCGGGGTGAA CCGTCAGCAC GTCCGGGTTC
8281 TTGCCTTCTG TGATCTCGCG GTACATCCAA TCAGCTAGCT CGATCTCGAT GTACTCCGGC
8341 CGCCCGGTTT CGCTCTTTAC GATCTTGTAG CGGCTAATCA AGGCTTCACC CTCGGATACC
8401 GTCACCAGGC GGCCGTTCTT GGCCTTCTTC GTACGCTGCA TGGCAACGTG CGTGGTGTTT
8461 AACCGAATGC AGGTTTCTAC CAGGTCGTCT TTCTGCTTTC CGCCATCGGC TCGCCGGCAG
8521 AACTTGAGTA CGTCCGCAAC GTGTGGACGG AACACGCGGC CGGGCTTGTC TCCCTTCCCT
8581 TCCCGGTATC GGTTCATGGA TTCGGTTAGA TGGGAAACCG CCATCAGTAC CAGGTCGTAA
8641 TCCCACACAC TGGCCATGCC GGCCGGCCCT GCGGAAACCT CTACGTGCCC GTCTGGAAGC
8701 TCGTAGCGGA TCACCTCGCC AGCTCGTCGG TCACGCTTCG ACAGACGGAA AACGGCCACG
8761 TCCATGATGC TGCGACTATC GCGGGTGCCC ACGTCATAGA GCATCGGAAC GAAAAAATCT
8821 GGTTGCTCGT CGCCCTTGGG CGGCTTCCTA ATCGACGGCG CACCGGCTGC CGGCGGTTGC
8881 CGGGATTCTT TGCGGATTCG ATCAGCGGCC GCTTGCCACG ATTCACCGGG GCGTGCTTCT
8941 GCCTCGATGC GTTGCCGCTG GGCGGCCTGC GCGGCCTTCA ACTTCTCCAC CAGGTCATCA
9001 CCCAGCGCCG CGCCGATTTG TACCGGGCCG GATGGTTTGC GACCGTCACG CCGATTCCTC
9061 GGGCTTGGGG GTTCCAGTGC CATTGCAGGG CCGGCAGACA ACCCAGCCGC TTACGCCTGG
9121 CCAACCGCCC GTTCCTCCAC ACATGGGGCA TTCCACGGCG TCGGTGCCTG GTTGTTCTTG
9181 ATTTTCCATG CCGCCTCCTT TAGCCGCTAA AATTCATCTA CTCATTTATT CATTTGCTCA
9241 TTTACTCTGG TAGCTGCGCG ATGTATTCAG ATAGCAGCTC GGTAATGGTC TTGCCTTGGC
9301 GTACCGCGTA CATCTTCAGC TTGGTGTGAT CCTCCGCCGG CAACTGAAAG TTGACCCGCT
9361 TCATGGCTGG CGTGTCTGCC AGGCTGGCCA ACGTTGCAGC CTTGCTGCTG CGTGCGCTCG
9421 GACGGCCGGC ACTTAGCGTG TTTGTGCTTT TGCTCATTTT CTCTTTACCT CATTAACTCA
9481 AATGAGTTTT GATTTAATTT CAGCGGCCAG CGCCTGGACC TCGCGGGCAG CGTCGCCCTC
9541 GGGTTCTGAT TCAAGAACGG TTGTGCCGGC GGCGGCAGTG CCTGGGTAGC TCACGCGCTG
9601 CGTGATACGG GACTCAAGAA TGGGCAGCTC GTACCCGGCC AGCGCCTCGG CAACCTCACC
9661 GCCGATGCGC GTGCCTTTGA TCGCCCGCGA CACGACAAAG GCCGCTTGTA GCCTTCCATC
9721 CGTGACCTCA ATGCGCTGCT TAACCAGCTC CACCAGGTCG GCGGTGGCCC ATATGTCGTA
9781 AGGGCTTGGC TGCACCGGAA TCAGCACGAA GTCGGCTGCC TTGATCGCGG ACACAGCCAA
9841 GTCCGCCGCC TGGGGCGCTC CGTCGATCAC TACGAAGTCG CGCCGGCCGA TGGCCTTCAC
9901 GTCGCGGTCA ATCGTCGGGC GGTCGATGCC GACAACGGTT AGCGGTTGAT CTTCCCGCAC
9961 GGCCGCCCAA TCGCGGGCAC TGCCCTGGGG ATCGGAATCG ACTAACAGAA CATCGGCCCC
10021 GGCGAGTTGC AGGGCGCGGG CTAGATGGGT TGCGATGGTC GTCTTGCCTG ACCCGCCTTT
10081 CTGGTTAAGT ACAGCGATAA CCTTCATGCG TTCCCCTTGC GTATTTGTTT ATTTACTCAT
10141 CGCATCATAT ACGCAGCGAC CGCATGACGC AAGCTGTTTT ACTCAAATAC ACATCACCTT
10201 TTTAGACGGC GGCGCTCGGT TTCTTCAGCG GCCAAGCTGG CCGGCCAGGC CGCCAGCTTG
10261 GCATCAGACA AACCGGCCAG GATTTCATGC AGCCGCACGG TTGAGACGTG CGCGGGCGGC
10321 TCGAACACGT ACCCGGCCGC GATCATCTCC GCCTCGATCT CTTCGGTAAT GAAAAACGGT
10381 TCGTCCTGGC CGTCCTGGTG CGGTTTCATG CTTGTTCCTC TTGGCGTTCA TTCTCGGCGG
10441 CCGCCAGGGC GTCGGCCTCG GTCAATGCGT CCTCACGGAA GGCACCGCGC CGCCTGGCCT
10501 CGGTGGGCGT CACTTCCTCG CTGCGCTCAA GTGCGCGGTA CAGGGTCGAG CGATGCACGC
10561 CAAGCAGTGC AGCCGCCTCT TTCACGGTGC GGCCTTCCTG GTCGATCAGC TCGCGGGCGT
10621 GCGCGATCTG TGCCGGGGTG AGGGTAGGGC GGGGGCCAAA CTTCACGCCT CGGGCCTTGG
10681 CGGCCTCGCG CCCGCTCCGG GTGCGGTCGA TGATTAGGGA ACGCTCGAAC TCGGCAATGC
10741 CGGCGAACAC GGTCAACACC ATGCGGCCGG CCGGCGTGGT GGTGTCGGCC CACGGCTCTG
10801 CCAGGCTACG CAGGCCCGCG CCGGCCTCCT GGATGCGCTC GGCAATGTCC AGTAGGTCGC
10861 GGGTGCTGCG GGCCAGGCGG TCTAGCCTGG TCACTGTCAC AACGTCGCCA GGGCGTAGGT
10921 GGTCAAGCAT CCTGGCCAGC TCCGGGCGGT CGCGCCTGGT GCCGGTGATC TTCTCGGAAA
10981 ACAGCTTGGT GCAGCCGGCC GCGTGCAGTT CGGCCCGTTG GTTGGTCAAG TCCTGGTCGT
11041 CGGTGCTGAC GCGGGCATAG CCCAGCAGGC CAGCGGCGGC GCTCTTGTTC ATGGCGTAAT
11101 GTCTCCGGTT CTAGTCGCAA GTATTCTACT TTATGCGACT AAAACACGCG ACAAGAAAAC
11161 GCCAGGAAAA GGGCAGGGCG GCAGCCTGTC GCGTAACTTA GGACTTGTGC GACATGTCGT
11221 TTTCAGAAGA CGGCTGCACT GAACGTCAGA AGCCGACTGC ACTATAGCAG CGGAGGGGTT
11281 GGATCAAAGT ACT
```

Supplemental Figure S2. F-SHMT08c-CDS, insert size, vector, restriction enzymes, and sequenced positive clones used for cloning.

Gene Name F-SHMT08cΔ+Y59A-CDS
Cloning Vector pG2RNAi2
Length (bp) 1430
Cloning Sites AscI-AvrII

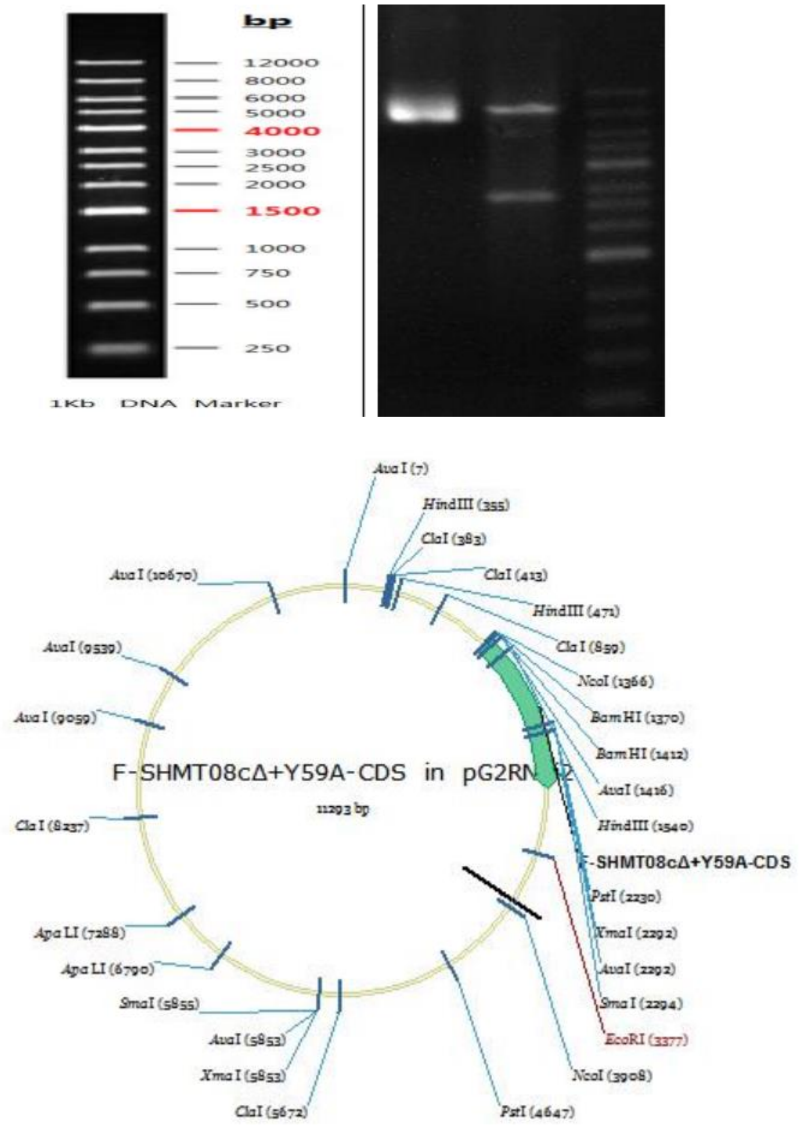

Supplemental Figure S3. F-SHMT08cΔ+Y59A-CDS, insert size, vector, restriction enzymes, and sequenced positive clones used for cloning.

Detailed Sequence of the Whole Construct:

```
   1 TTGATCCCGA GGGGAACCCT GTGGTTGGCA TGCACATACA AATGGACGAA CGGATAAACC
  61 TTTTCACGCC CTTTTAAATA TCCGTTATTC TAATAAACGC TCTTTTCTCT TAGGTTTACC
 121 CGCCAATATA TCCTGTCAAA CACTGATAGT TTAAACTGAA GGCGGGAAAC GACAATCTGA
 181 TCCAAGCTCA AGCTCATTCG CCATTCAGGC TGCGCAACTG TTGGGAAGGG CGATCGGTGC
 241 GGGCCTCTTC GCTATTACGC CAGCTGGCGA AAGGGGGATG TGCTGCAAGG CGATTAAGTT
 301 GGGTAACGCC AGGGTTTTCC CAGTCACGAC GTTGTAAAAC GACGGCCAGT GCCAAGCTTA
 361 TTACCCTGTT ATCCCTAGAT ATCGATTTTG GCTACCTTAA GGTAGCCAAA ATCGATCACT
 421 AGTGCGGCCG CGGGCCCAAT ATAACAACGA CGTCGTAACA GATAAAGCGA AGCTTGAAGG
 481 TGCATGTGAC TCCGTCAAGA TTACGAAACC GCCAACTACC ACGCAAATTG CAATTCTCAA
 541 TTTCCTAGAA GGACTCTCCG AAAATGCATC CAATACCAAA TATTACCCGT GTCATAGGCA
 601 CCAAGTGACA CCATACATGA ACACGCGTCA CAATATGACT GGAGAAGGGT TCCACACCTT
 661 ATGCTATAAA ACGCCCCACA CCCCTCCTCC TTCCTTCGCA GTTCAATTCC AATATATTCC
 721 ATTCTCTCTG TGTATTTCCC TACCTCTCCC TTCAAGGTTA GTCGATTTCT TCTGTTTTTC
 781 TTCTTCGTTC TTTCCATGAA TTGTGTATGT TCTTTGATCA ATACGATGTT GATTTGATTG
 841 TGTTTTGTTT GGTTTCATCG ATCTTCAATT TTCATAATCA GATTCAGCTT TTATTATCTT
 901 TACAACAACG TCCTTAATTT GATGATTCTT TAATCGTAGA TTTGCTCTAA TTAGAGCTTT
 961 TTCATGTCAG ATCCCTTTAC AACAAGCCTT AATTGTTGAT TCATTAATCG TAGATTAGGG
1021 CTTTTTTCAT TGATTACTTC AGATCCGTTA AACGTAACCA TAGATCAGGG CTTTTTCATG
1081 AATTACTTCA GATCCGTTAA ACAACAGCCT TATTTTTTAT ACTTCTGTGG TTTTTCAAGA
1141 AATTGTTCAG ATCCGTTGAC AAAAAGCCTT ATTCGTTGAT TCTATATCGT TTTTCGAGAG
1201 ATATTGCTCA GATCTGTTAG CAACTGCCTT GTTTGTTGAT TCTATTGCCG TGGATTAGGG
1261 TTTTTTTTCA CGAGATTGCT TCAGATCCGT ACTTAAGATT ACGTAATGGA TTTTGATTCT
1321 GATTTATCTG TGATTGTTGA CTCGACAGCG GCCGCACCGG CGCGCCATGG ATCCAGTAAG
1381 CGTGTGGGGT AACACGCCCT TGGCGACGGT GGATCCCGAG ATCCATGACC TCATCGAGAA
1441 GGAGAAGCGC CGTCAATGCC GCGGAATCGA GCTCATCGCC TCCGAGAACT TCACCTCCTT
1501 CGCCGTCATC GAGGCCCTCG GCAGCGCTCT CACGAACAAA GCTTCCGAGG GCATGCCGGG
```

Supplemental Figure S3. F-SHMT08cΔ+Y59A-CDS, insert size, vector, restriction enzymes, and sequenced positive clones used for cloning.

1561 CAACCGCTAC TACGGCGGCA ATGAATACAT CGACCAGATC GAAAACCTCT GCCGCTCACG
1621 CGCCCTCCAA GCCTTCCACC TCGACGCCCA ATCCTGGGGC GTCAACGTCC AGCCCTACTC
1681 CGGCTCCCCG GCCAACTTCG CCGCCTACAC CGCCGTCCTC AACCCCCACG ACCGCATCAT
1741 GGGGCTAGAT CTCCGCTCCG GCGGCCACCT CACCCACGGC TACTACACCT CCGGCGGAAA
1801 GAAGATCTCC GCCACCTCCA TTTACTTCGA GAGTCTCCCT TACAAGGTAA ACTCCACCAC
1861 CGGCTACATC GACTACGACC GCTTGGAAGA AAAAGCCCTA GACTTCAGGC CAAAACTCAT
1921 AATCTGCGGT GGCAGCGCGT ACCCTCGCGA TTGGGACTAC AAACGTTTCA GGGAAGTCGC
1981 TGATAAGTGC GGAGCATTGC TTCTCTGCGA CATGGCGCAC ACTAGCGGCC TTGTGGCCGC
2041 GCAGGAAGTG AACAGCCCCT TCGAGTATTG CGACATTGTG ACCACCACGA CTCACAAGAG
2101 CTTGCGGGGC CCACGTGCGG GGATGATCTT TTACCGGAAG GGCCCCAAGC CGCCGAAGAA
2161 GGGGCAGCCG GAGAACGCGG TTTATGATTT CGAGGACAAG ATTAACTTCG CGGTGTTCCC
2221 TTCGCTGCAG GGTGGGCCCC ACAACCACCA GATCGGTGCT CTCGCCGTGG CGCTGAAGCA
2281 GGCCGCGTCG CCCGGGTTTA AGGCCTACGC GAAGCAGGTT AAGGCGAACG CCGTTGCGCT
2341 TGGAAAATAC TTGATGGGGA AAGGGTACAG CCTTGTCACT GGCGGAACGG AGAACCATCT
2401 TGTTTTGTGG GATCTGAGAC CTCTTGGATT GACTGGGTAT AAGGTGGAGA AACTCTGTGA
2461 TCTCTGTAAC ATTACTGTTA ACAAGAACGC TGTTTTTGGT GATAGCAGTG CCTTGGCCCC
2521 TGGTGGAGTG CGAATTGGTG CCCCTGCCAT GACTTCTAGG GGTTTGGTTG AAAAAGACTT
2581 TGAGCAGATT GGTGAGTTCC TTCACCGTGC TGTGACTCTC ACACTGGAGA TCCAGAAGGA
2641 GCATGGCAAA CTTCTCAAGG ATTTCAACAA GGGTCTCGTC AACAACAAGG CTATTGAAGA
2701 TCTCAAAGCT GATGTTGAGA AGTTCTCTGC CTTGTTTGAC ATGCCTGGCT TCCTGGTATC
2761 TGAAATGAAG TACAAGGATT AGCCTAGGTT CGAGTATTAT GGCATTGGGA AAACTGTTTT
2821 TCTTGTACCA TTTGTTGTGC TTGTAATTTA CTGTGTTTTT TATTCGGTTT TCGCTATCGA
2881 ACTGTGAAAT GGAAATGGAT GGAGAAGAGT TAATGAATGA TATGGTCCTT TTGTTCATTC
2941 TCAAATTAAT ATTATTTGTT TTTTCTCTTA TTTGTTGTGT GTTGAATTTG AAATTATAAG
3001 AGATATGCAA ACATTTTGTT TTGAGTAAAA ATGTGTCAAA TCGTGGCCTC TAATGACCGA
3061 AGTTAATATG AGGAGTAAAA CACTTGTAGT TGTACCATTA TGCTTATTCA CTAGGCAACA
3121 AATATATTTT CAGACCTAGA AAAGCTGCAA ATGTTACTGA ATACAAGTAT GTCCTCTTGT
3181 GTTTTAGACA TTTATGAACT TTCCTTTATG TAATTTTCCA GAATCCTTGT CAGATTCTAA
3241 TCATTGCTTT ATAATTATAG TTATACTCAT GGATTTGTAG TTGAGTATGA AAATATTTTT
3301 TAATGCATTT TATGACTTGC CAATTGATTG ACAACATGCA TCAATCCGCG GTTATGACTC
3361 TCTTAAGAGA GTCATGAATT CGAGCTTCCA GAAGGTAATT ATCCAAGATG TAGCATCAAG
3421 AATCCAATGT TTACGGGAAA AACTATGGAA GTATTATGTG AGCTCAGCAA GAAGCAGATC
3481 AATATGCGGC ACATATGCAA CCTATGTTCA AAAATGAAGA ATGTACAGAT ACAAGATCCT
3541 ATACTGCCAG AATACGAAGA AGAATACGTA GAAATTGAAA AAGAAGAACC AGGCGAAGAA
3601 AAGAATCTTG AAGACGTAAG CACTGACGAC AACAATGAAA AGAAGAAGAT AAGGTCGGTG
3661 ATTGTGAAAG AGACATAGAG GACACATGTA AGGTGGAAAA TGTAAGGGCG GAAAGTAACC
3721 TTATCACAAA GGAATCTTAT CCCCCACTAC TTATCCTTTT ATATTTTTCC GTGTCATTTT
3781 TGCCCTTGAG TTTTCCTATA TAAGGAACCA AGTTCGGCAT TTGTGAAAAC AAGAAAAAAT
3841 TTGGTGTAAG CTATTTTCTT TGAAGTACTG AGGATACAAC TTCAGAGAAA TTTGTAAGTT
3901 TGTGATCCAT GGTGAGCAAG GGCGAGGAGC TGTTCACCGG GGTGGTGCCC ATCCTGGTCG
3961 AGCTGGACGG CGACGTAAAC GGCCACAAGT TCAGCGTGTC CGGCGAGGGC GAGGGCGATG
4021 CCACCTACGG CAAGCTGACC CTGAAGTTCA TCTGCACCAC CGGCAAGCTG CCCGTGCCCT
4081 GGCCCACCCT CGTGACCACC TTCACCTACG GCGTGCAGTG CTTCAGCCGC TACCCCGACC
4141 ACATGAAGCA GCACGACTTC TTCAAGTCCG CCATGCCCGA AGGCTACGTC CAGGAGCGCA
4201 CCATCTTCTT CAAGGACGAC GGCAACTACA AGACCCGCGC CGAGGTGAAG TTCGAGGGCG
4261 ACACCCTGGT GAACCGCATC GAGCTGAAGG GCATCGACTT CAAGGAGGAC GGCAACATCC
4321 TGGGGCACAA GCTGGAGTAC AACTACAACA GCCACAACGT CTATATCATG GCCGACAAGC
4381 AGAAGAACGG CATCAAGGTG AACTTCAAGA TCCGCCACAA CATCGAGGAC GGCAGCGTGC
4441 AGCTCGCCGA CCACTACCAG CAGAACACCC CCATCGGCGA CGGCCCCGTG CTGCTGCCCG
4501 ACAACCACTA CCTGAGCACC CAGTCCGCCC TGAGCAAAGA CCCCAACGAG AAGCGCGATC
4561 ACATGGTCCT GCTGGAGTTC GTGACCGCCG CCGGGATCAC TCACGGCATG GACGAGCTGT
4621 ACAAGTAAAG CGGCCGCCCG GCTGCAGATC GTTCAAACAT TTGGCAATAA AGTTTCTTAA
4681 GATTGAATCC TGTTGCCGGT CTTGCGATGA TTATCATATA ATTTCTGTTG AATTACGTTA
4741 AGCATGTAAT AATTAACATG TAATGCATGA CGTTATTTAT GAGATGGGTT TTTATGATTA

Supplemental Figure S3. F-SHMT08cΔ+Y59A-CDS, insert size, vector, restriction enzymes, and sequenced positive clones used for cloning.

4801 GAGTCCCGCA ATTATACATT TAATACGCGA TAGAAAACAA AATATAGCGC GCAAACTAGG
4861 ATAAATTATC GCGCGCGGTG TCATCTATGT TACTAGATCC GATGATAAGC TGTCAAACAT
4921 GAGAATTAAT TCGTAATCAT GTCATAGCTG TTTCCTGTGT GAAATTGTTA TCCGCTCACA
4981 ATTCCACACA ACATACGAGC CGGAAGCATA AAGTGTAAAG CCTGGGGTGC CTAATGAGTG
5041 AGCTAACTCA CATTAATTGC GTTGCGCTCA CTGCCCGCTT TCCAGTCGGG AAACCTGTCG
5101 TGCCAGCTGC ATTAATGAAT CGGCCAACGC GCGGGGAGAG GCGGTTTGCG TATTGGCTAG
5161 AGCAATTCGG CGTTAATTCA GTACATTAAA AACGTCCGCA ATGTGTTATT AAGTTGTCTA
5221 AGCGTCAATT TGTTTACACC ACAATATATC CTGCCACCAG CCAGCCAACA GCTCCCCGAC
5281 CGGCAGCTCG GCACAAAATC ACCACTCGAT ACAGGCAGCC CATCAGTCCG GGACGGCGTC
5341 AGCGGGAGAG CCGTTGTAAG GCGGCAGACT TTGCTCATGT TACCGATGCT ATTCGGAAGA
5401 ACGGCAACTA AGCTGCCGGG TTTGAAACAC GGATGATCTC GCGGAGGGTA GCATGTTGAT
5461 TGTAACGATG ACAGAGCGTT GCTGCCTGTG ATCCAGATCA TGAACAATAA AACTGTCTGC
5521 TTACATAAAC AGTAATACAA GGGGTGTTAT GAGCCATATT CAACGGGAAA CGTCTTGCTC
5581 TAGGCCGCGA TTAAATTCCA ACATGGATGC TGATTTATAT GGGTATAAAT GGGCTCGCGA
5641 TAATGTCGGG CAATCAGGTG CGACAATCTA TCGATTGTAT GGGAAGCCCG ATGCGCCAGA
5701 GTTGTTTCTG AAACATGGCA AAGGTAGCGT TGCCAATGAT GTTACAGATG AGATGGTCAG
5761 ACTAAACTGG CTGACGGAAT TTATGCCTCT TCCGACCATC AAGCATTTTA TCCGTACTCC
5821 TGATGATGCA TGGTTACTCA CCACTGCGAT CCCCGGGAAA ACAGCATTCC AGGTATTAGA
5881 AGAATATCCT GATTCAGGTG AAAATATTGT TGATGCGCTG GCAGTGTTCC TGCGCCGGTT
5941 GCATTCGATT CCTGTTTGTA ATTGTCCTTT TAACAGCGAT CGCGTATTTC GTCTCGCTCA
6001 GGCGCAATCA CGAATGAATA ACGGTTTGGT TGATGCGAGT GATTTTGATG ACGAGCGTAA
6061 TGGCTGGCCT GTTGAACAAG TCTGGAAAGA AATGCATAAA CTTTTGCCAT TCTCACCGGA
6121 TTCAGTCGTC ACTCATGGTG ATTTCTCACT TGATAACCTT ATTTTTGACG AGGGGAAATT
6181 AATAGGTTGT ATTGATGTTG GACGAGTCGG AATCGCAGAC CGATACCAGG ATCTTGCCAT
6241 CCTATGGAAC TGCCTCGGTG AGTTTTCTCC TTCATTACAG AAACGGCTTT TTCAAAAATA
6301 TGGTATTGAT AATCCTGATA TGAATAAATT GCAGTTTCAT TTGATGCTCG ATGAGTTTTT
6361 CTAAGAATTA ATTCATGATC CTGCATGACC AAAATCCCTT AACGTGAGTT TTCGTTCCAC
6421 TGAGCGTCAG ACCCCGTAGA AAAGATCAAA GGATCTTCTT GAGATCCTTT TTTTCTGCGC
6481 GTAATCTGCT GCTTGCAAAC AAAAAAACCA CCGCTACCAG CGGTGGTTTG TTTGCCGGAT
6541 CAAGAGCTAC CAACTCTTTT TCCGAAGGTA ACTGGCTTCA GCAGAGCGCA GATACCAAAT
6601 ACTGTCCTTC TAGTGTAGCC GTAGTTAGGC CACCACTTCA AGAACTCTGT AGCACCGCCT
6661 ACATACCTCG CTCTGCTAAT CCTGTTACCA GTGGCTGCTG CCAGTGGCGA TAAGTCGTGT
6721 CTTACCGGGT TGGACTCAAG ACGATAGTTA CCGGATAAGG CGCAGCGGTC GGGCTGAACG
6781 GGGGGTTCGT GCACACAGCC CAGCTTGGAG CGAACGACCT ACACCGAACT GAGATACCTA
6841 CAGCGTGAGC TATGAGAAAG CGCCACGCTT CCCGAAGGGA GAAAGGCGGA CAGGTATCCG
6901 GTAAGCGGCA GGGTCGGAAC AGGAGAGCGC ACGAGGGAGC TTCCAGGGGG AAACGCCTGG
6961 TATCTTTATA GTCCTGTCGG GTTTCGCCAC CTCTGACTTG AGCGTCGATT TTTGTGATGC
7021 TCGTCAGGGG GGCGGAGCCT ATGGAAAAAC GCCAGCAACG CGGCCTTTTT ACGGTTCCTG
7081 GCCTTTTGCT GGCCTTTTGC TCACATGTTC TTTCCTGCGT TATCCCCTGA TTCTGTGGAT
7141 AACCGTATTA CCGCCTTTGA GTGAGCTGAT ACCGCTCGCC GCAGCCGAAC GACCGAGCGC
7201 AGCGAGTCAG TGAGCGAGGA AGCGGAAGAG CGCCTGATGC GGTATTTTCT CCTTACGCAT
7261 CTGTGCGGTA TTTCACACCG CATATGGTGC ACTCTCAGTA CAATCTGCTC TGATGCCGCA
7321 TAGTTAAGCC AGTATACACT CCGCTATCGC TACGTGACTG GGTCATGGCT GCGCCCCGAC
7381 ACCCGCCAAC ACCCGCTGAC GCGCCCTGAC GGGCTTGTCT GCTCCCGGCA TCCGCTTACA
7441 GACAAGCTGT GACCGTCTCC GGGAGCTGCA TGTGTCAGAG GTTTTCACCG TCATCACCGA
7501 AACGCGCGAG GCAGGGTGCC TTGATGTGGG CGCCGGCGGT CGAGTGGCGA CGGCGCGGCT
7561 TGTCCGCGCC CTGGTAGATT GCCTGGCCGT AGGCCAGCCA TTTTTGAGCG GCCAGCGGCC
7621 GCGATAGGCC GACGCGAAGC GGCGGGGCGT AGGGAGCGCA GCGACCGAAG GGTAGGCGCT
7681 TTTTGCAGCT CTTCGGCTGT GCGCTGGCCA GACAGTTATG CACAGGCCAG GCGGGTTTTA
7741 AGAGTTTTAA TAAGTTTTAA AGAGTTTTAG GCGGAAAAAT CGCCTTTTTT CTCTTTTATA
7801 TCAGTCACTT ACATGTGTGA CCGGTTCCCA ATGTACGGCT TTGGGTTCCC AATGTACGGG
7861 TTCCGGTTCC CAATGTACGG CTTTGGGTTC CCAATGTACG TGCTATCCAC AGGAAAGAGA
7921 CCTTTTCGAC CTTTTTCCCC TGCTAGGGCA ATTTGCCCTA GCATCTGCTC CGTACATTAG
7981 GAACCGGCGG ATGCTTCGCC CTCGATCAGG TTGCGGTAGC GCATGACTAG GATCGGGCCA
8041 GCCTGCCCCG CCTCCTCCTT CAAATCGTAC TCCGGCAGGT CATTTGACCC GATCAGCTTG
8101 CGCACGGTGA AACAGAACTT CTTGAACTCT CCGGCGCTGC CACTGCGTTC GTAGATCGTC
8161 TTGAACAACC ATCTGGCTTC TGCCTTGCCT GCGGCGCGGC GTGCCAGGCG GTAGAGAAAA
8221 CGGCCGATGC CGGGATCGAT CAAAAAGTAA TCGGGGTGAA CCGTCAGCAC GTCCGGGTTC
8281 TTGCCTTCTG TGATCTCGCG GTACATCCAA TCAGCTAGCT CGATCTCGAT GTACTCCGGC
8341 CGCCCGGTTT CGCTCTTTAC GATCTTGTAG CGGCTAATCA AGGCTTCACC CTCGGATACC
8401 GTCACCAGGC GGCCGTTCTT GGCCTTCTTC GTACGCTGCA TGGCAACGTG CGTGGTGTTT
8461 AACCGAATGC AGGTTTCTAC CAGGTCGTCT TTCTGCTTTC CGCCATCGGC TCGCCGGCAG
8521 AACTTGAGTA CGTCCGCAAC GTGTGGACGG AACACGCGGC CGGGCTTGTC TCCCTTCCCT
8581 TCCCGGTATC GGTTCATGGA TTCGGTTAGA TGGGAAACCG CCATCAGTAC CAGGTCGTAA
8641 TCCCACACAC TGGCCATGCC GGCCGGCCCT GCGGAAACCT CTACGTGCCC GTCTGGAAGC
8701 TCGTAGCGGA TCACCTCGCC AGCTCGTCGG TCACGCTTCG ACAGACGGAA AACGGCCACG
8761 TCCATGATGC TGCGACTATC GCGGGTGCCC ACGTCATAGA GCATCGGAAC GAAAAAATCT
8821 GGTTGCTCGT CGCCCTTGGG CGGCTTCCTA ATCGACGGCG CACCGGCTGC CGGCGGTTGC
8881 CGGGATTCTT TGCGGATTCG ATCAGCGGCC GCTTGCCACG ATTCACCGGG GCGTGCTTCT
8941 GCCTCGATGC GTTGCCGCTG GGCGGCCTGC GCGGCCTTCA ACTTCTCCAC CAGGTCATCA
9001 CCCAGCGCCG CGCCGATTTG TACCGGGCCG GATGGTTTGC GACCGTCACG CCGATTCCTC
9061 GGGCTTGGGG GTTCCAGTGC CATTGCAGGG CCGGCAGACA ACCCAGCCGC TTACGCCTGG
9121 CCAACCGCCC GTTCCTCCAC ACATGGGGCA TTCCACGGCG TCGGTGCCTG GTTGTTCTTG
9181 ATTTTCCATG CCGCCTCCTT TAGCCGCTAA AATTCATCTA CTCATTTATT CATTTGCTCA
9241 TTTACTCTGG TAGCTGCGCG ATGTATTCAG ATAGCAGCTC GGTAATGGTC TTGCCTTGGC
9301 GTACCGCGTA CATCTTCAGC TTGGTGTGAT CCTCCGCCGG CAACTGAAAG TTGACCCGCT
9361 TCATGGCTGG CGTGTCTGCC AGGCTGGCCA ACGTTGCAGC CTTGCTGCTG CGTGCGCTCG
9421 GACGGCCGGC ACTTAGCGTG TTTGTGCTTT TGCTCATTTT CTCTTTACCT CATTAACTCA
9481 AATGAGTTTT GATTTAATTT CAGCGGCCAG CGCCTGGACC TCGCGGGCAG CGTCGCCCTC
9541 GGGTTCTGAT TCAAGAACGG TTGTGCCGGC GGCGGCAGTG CCTGGGTAGC TCACGCGCTG
9601 CGTGATACGG GACTCAAGAA TGGGCAGCTC GTACCCGGCC AGCGCCTCGG CAACCTCACC
9661 GCCGATGCGC GTGCCTTTGA TCGCCCGCGA CACGACAAAG GCCGCTTGTA GCCTTCCATC
9721 CGTGACCTCA ATGCGCTGCT TAACCAGCTC CACCAGGTCG GCGGTGGCCC ATATGTCGTA
9781 AGGGCTTGGC TGCACCGGAA TCAGCACGAA GTCGGCTGCC TTGATCGCGG ACACAGCCAA
9841 GTCCGCCGCC TGGGGCGCTC CGTCGATCAC TACGAAGTCG CGCCGGCCGA TGGCCTTCAC
9901 GTCGCGGTCA ATCGTCGGGC GGTCGATGCC GACAACGGTT AGCGGTTGAT CTTCCCGCAC
9961 GGCCGCCCAA TCGCGGGCAC TGCCCTGGGG ATCGGAATCG ACTAACAGAA CATCGGCCCC
10021 GGCGAGTTGC AGGGCGCGGG CTAGATGGGT TGCGATGGTC GTCTTGCCTG ACCCGCCTTT
10081 CTGGTTAAGT ACAGCGATAA CCTTCATGCG TTCCCCTTGC GTATTTGTTT ATTTACTCAT
10141 CGCATCATAT ACGCAGCGAC CGCATGACGC AAGCTGTTTT ACTCAAATAC ACATCACCTT
10201 TTTAGACGGC GGCGCTCGGT TTCTTCAGCG GCCAAGCTGG CCGGCCAGGC CGCCAGCTTG
10261 GCATCAGACA AACCGGCCAG GATTTCATGC AGCCGCACGG TTGAGACGTG CGCGGGCGGC
10321 TCGAACACGT ACCCGGCCGC GATCATCTCC GCCTCGATCT CTTCGGTAAT GAAAAACGGT
10381 TCGTCCTGGC CGTCCTGGTG CGGTTTCATG CTTGTTCCTC TTGGCGTTCA TTCTCGGCGG
10441 CCGCCAGGGC GTCGGCCTCG GTCAATGCGT CCTCACGGAA GGCACCGCGC CGCCTGGCCT
10501 CGGTGGGCGT CACTTCCTCG CTGCGCTCAA GTGCGCGGTA CAGGGTCGAG CGATGCACGC
10561 CAAGCAGTGC AGCCGCCTCT TTCACGGTGC GGCCTTCCTG GTCGATCAGC TCGCGGGCGT
10621 GCGCGATCTG TGCCGGGGTG AGGGTAGGGC GGGGGCCAAA CTTCACGCCT CGGGCCTTGG
10681 CGGCCTCGCG CCCGCTCCGG GTGCGGTCGA TGATTAGGGA ACGCTCGAAC TCGGCAATGC
10741 CGGCGAACAC GGTCAACACC ATGCGGCCGG CCGGCGTGGT GGTGTCGGCC CACGGCTCTG
10801 CCAGGCTACG CAGGCCCGCG CCGGCCTCCT GGATGCGCTC GGCAATGTCC AGTAGGTCGC
10861 GGGTGCTGCG GGCCAGGCGG TCTAGCCTGG TCACTGTCAC AACGTCGCCA GGGCGTAGGT
10921 GGTCAAGCAT CCTGGCCAGC TCCGGGCGGT CGCGCCTGGT GCCGGTGATC TTCTCGGAAA
10981 ACAGCTTGGT GCAGCCGGCC GCGTGCAGTT CGGCCCGTTG GTTGGTCAAG TCCTGGTCGT
11041 CGGTGCTGAC GCGGGCATAG CCCAGCAGGC CAGCGGCGGC GCTCTTGTTC ATGGCGTAAT
11101 GTCTCCGGTT CTAGTCGCAA GTATTCTACT TTATGCGACT AAAACACGCG ACAAGAAAAC
11161 GCCAGGAAAA GGGCAGGGCG GCAGCCTGTC GCGTAACTTA GGACTTGTGC GACATGTCGT
11221 TTTCAGAAGA CGGCTGCACT GAACGTCAGA AGCCGACTGC ACTATAGCAG CGGAGGGGTT
11281 GGATCAAAGT ACT

Supplemental Figure S3. F-SHMT08cΔ+Y59A-CDS, insert size, vector, restriction enzymes, and sequenced positive clones used for cloning.

Gene Name F-SHMT08cΔ+L129A-CDS
Cloning Vector pG2RNAi2
Length (bp) 1430
Cloning Sites AscI-AvrII

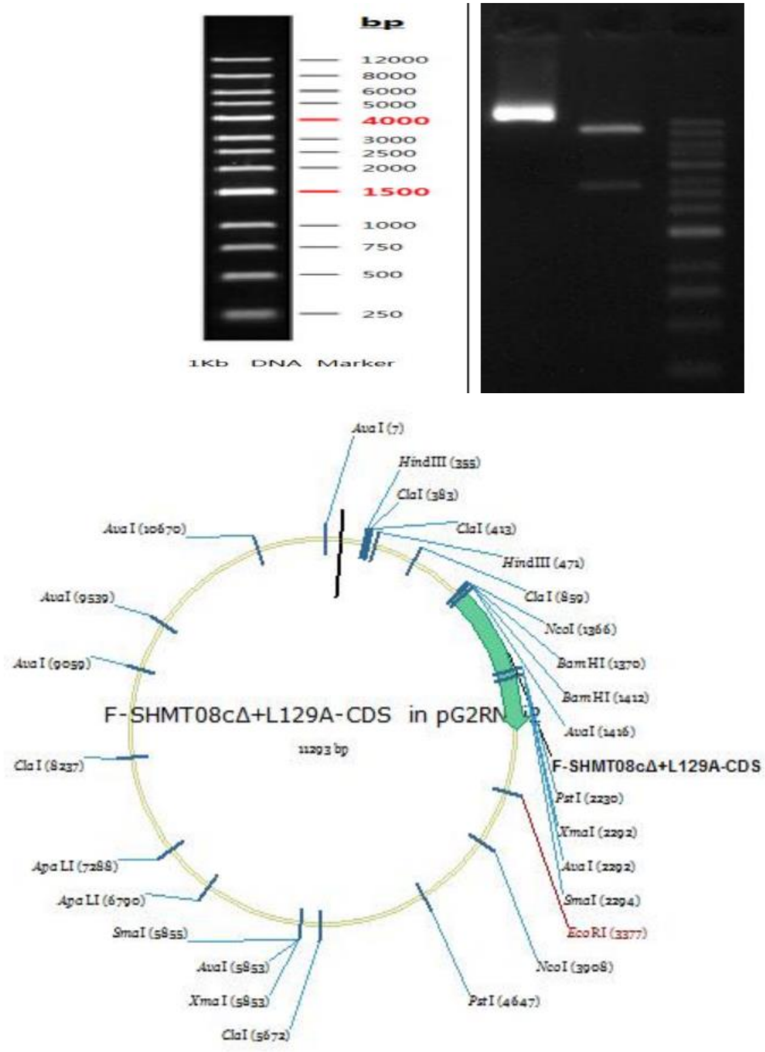

Supplemental Figure S4. F-SHMT08cΔ+L129A-CDS, insert size, vector, restriction enzymes, and sequenced positive clones used for cloning.

Detailed Sequence of the Whole Construct:

```
   1 TTGATCCCGA GGGGAACCCT GTGGTTGGCA TGCACATACA AATGGACGAA CGGATAAACC
  61 TTTTCACGCC CTTTTAAATA TCCGTTATTC TAATAAACGC TCTTTTCTCT TAGGTTTACC
 121 CGCCAATATA TCCTGTCAAA CACTGATAGT TTAAACTGAA GGCGGGAAAC GACAATCTGA
 181 TCCAAGCTCA AGCTCATTCG CCATTCAGGC TGCGCAACTG TTGGGAAGGG CGATCGGTGC
 241 GGGCCTCTTC GCTATTACGC CAGCTGGCGA AAGGGGGATG TGCTGCAAGG CGATTAAGTT
 301 GGGTAACGCC AGGGTTTTCC CAGTCACGAC GTTGTAAAAC GACGGCCAGT GCCAAGCTTA
 361 TTACCCTGTT ATCCCTAGAT ATCGATTTTG GCTACCTTAA GGTAGCCAAA ATCGATCACT
 421 AGTGCGGCCG CGGGCCCAAT ATAACAACGA CGTCGTAACA GATAAAGCGA AGCTTGAAGG
 481 TGCATGTGAC TCCGTCAAGA TTACGAAACC GCCAACTACC ACGCAAATTG CAATTCTCAA
 541 TTTCCTAGAA GGACTCTCCG AAAATGCATC CAATACCAAA TATTACCCGT GTCATAGGCA
 601 CCAAGTGACA CCATACATGA ACACGCGTCA CAATATGACT GGAGAAGGGT TCCACACCTT
 661 ATGCTATAAA ACGCCCCACA CCCCTCCTCC TTCCTTCGCA GTTCAATTCC AATATATTCC
 721 ATTCTCTCTG TGTATTTCCC TACCTCTCCC TTCAAGGTTA GTCGATTTCT TCTGTTTTTC
 781 TTCTTCGTTC TTTCCATGAA TTGTGTATGT TCTTTGATCA ATACGATGTT GATTTGATTG
 841 TGTTTTGTTT GGTTTCATCG ATCTTCAATT TTCATAATCA GATTCAGCTT TTATTATCTT
 901 TACAACAACG TCCTTAATTT GATGATTCTT TAATCCTAGA TTTGCTCTAA TTACACCTTT
 961 TTCATGTCAG ATCCCTTTAC AACAAGCCTT AATTGTTGAT TCATTAATCG TAGATTAGGG
1021 CTTTTTTCAT TGATTACTTC AGATCCGTTA AACGTAACCA TAGATCAGGG CTTTTTCATG
1081 AATTACTTCA GATCCGTTAA ACAACAGCCT TATTTTTTAT ACTTCTGTGG TTTTTCAAGA
1141 AATTGTTCAG ATCCGTTGAC AAAAAGCCTT ATTCGTTGAT TCTATATCGT TTTTCGAGAG
1201 ATATTGCTCA GATCTGTTAG CAACTGCCTT GTTTGTTGAT TCTATTGCCG TGGATTAGGG
1261 TTTTTTTTCA CGAGATTGCT TCAGATCCGT ACTTAAGATT ACGTAATGGA TTTTGATTCT
1321 GATTTATCTG TGATTGTTGA CTCGACAGCG GCCGCACCGG CGCGCCATGG ATCCAGTAAG
1381 CGTGTGGGGT AACACGCCCT TGGCGACGGT GGATCCCGAG ATCCATGACC TCATCGAGAA
1441 GGAGAAGCGC CGTCAATGCC GCGGAATCGA GCTCATCGCC TCCGAGAACT TCACCTCCTT
```

Supplemental Figure S4. F-SHMT08cΔ+L129A-CDS, insert size, vector, restriction enzymes, and sequenced positive clones used for cloning.

```
1501 CGCCGTCATC GAGGCCCTCG GCAGCGCTCT CACGAACAAA TACTCCGAGG GCATGCCGGG
1561 CAACCGCTAC TACGGCGGCA ATGAATACAT CGACCAGATC GAAAACCTCT GCCGCTCACG
1621 CGCCCTCCAA GCCTTCCACC TCGACGCCCA ATCCTGGGGC GTCAACGTCC AGCCCTACTC
1681 CGGCTCCCCG GCCAACTTCG CCGCCTACAC CGCCGTCCTC AACCCCCACG ACCGCATCAT
1741 GGGGCTAGAT GCTCGCTCCG GCGGCCACCT CACCCACGGC TACTACACCT CCGGCGGAAA
1801 GAAGATCTCC GCCACCTCCA TTTACTTCGA GAGTCTCCCT TACAAGGTAA ACTCCACCAC
1861 CGGCTACATC GACTACGACC GCTTGGAAGA AAAAGCCCTA GACTTCAGGC CAAAACTCAT
1921 AATCTGCGGT GGCAGCGCGT ACCCTCGCGA TTGGGACTAC AAACGTTTCA GGGAAGTCGC
1981 TGATAAGTGC GGAGCATTGC TTCTCTGCGA CATGGCGCAC ACTAGCGGCC TTGTGGCCGC
2041 GCAGGAAGTG AACAGCCCCT TCGAGTATTG CGACATTGTG ACCACCACGA CTCACAAGAG
2101 CTTGCGGGGC CCACGTGCGG GGATGATCTT TTACCGGAAG GGCCCCAAGC CGCCGAAGAA
2161 GGGGCAGCCG GAGAACGCGG TTTATGATTT CGAGGACAAG ATTAACTTCG CGGTGTTCCC
2221 TTCGCTGCAG GGTGGGCCCC ACAACCACCA GATCGGTGCT CTCGCCGTGG CGCTGAAGCA
2281 GGCCGCGTCG CCCGGGTTTA AGGCCTACGC GAAGCAGGTT AAGGCGAACG CCGTTGCGCT
2341 TGGAAAATAC TTGATGGGGA AAGGGTACAG CCTTGTCACT GGCGGAACGG AGAACCATCT
2401 TGTTTTGTGG GATCTGAGAC CTCTTGGATT GACTGGGTAT AAGGTGGAGA AACTCTGTGA
2461 TCTCTGTAAC ATTACTGTTA ACAAGAACGC TGTTTTTGGT GATAGCAGTG CCTTGGCCCC
2521 TGGTGGAGTG CGAATTGGTG CCCCTGCCAT GACTTCTAGG GGTTTGGTTG AAAAAGACTT
2581 TGAGCAGATT GGTGAGTTCC TTCACCGTGC TGTGACTCTC ACACTGGAGA TCCAGAAGGA
2641 GCATGGCAAA CTTCTCAAGG ATTTCAACAA GGGTCTCGTC AACAACAAGG CTATTGAAGA
2701 TCTCAAAGCT GATGTTGAGA AGTTCTCTGC CTTGTTTGAC ATGCCTGGCT TCCTGGTATC
2761 TGAAATGAAG TACAAGGATT AGCCTAGGTT CGAGTATTAT GGCATTGGGA AAACTGTTTT
2821 TCTTGTACCA TTTGTTGTGC TTGTAATTTA CTGTGTTTTT TATTCGGTTT TCGCTATCGA
2881 ACTGTGAAAT GGAAATGGAT GGAGAAGAGT TAATGAATGA TATGGTCCTT TTGTTCATTC
2941 TCAAATTAAT ATTATTTGTT TTTTCTCTTA TTTGTTGTGT GTTGAATTTG AAATTATAAG
3001 AGATATGCAA ACATTTTGTT TTGAGTAAAA ATGTGTCAAA TCGTGGCCTC TAATGACCGA
3061 AGTTAATATG AGGAGTAAAA CACTTGTAGT TGTACCATTA TGCTTATTCA CTAGGCAACA
3121 AATATATTTT CAGACCTAGA AAAGCTGCAA ATGTTACTGA ATACAAGTAT GTCCTCTTGT
3181 GTTTTAGACA TTTATGAACT TTCCTTTATG TAATTTTCCA GAATCCTTGT CAGATTCTAA
3241 TCATTGCTTT ATAATTATAG TTATACTCAT GGATTTGTAG TTGAGTATGA AAATATTTTT
3301 TAATGCATTT TATGACTTGC CAATTGATTG ACAACATGCA TCAATCCGCG GTTATGACTC
3361 TCTTAAGAGA GTCATGAATT CGAGCTTCCA GAAGGTAATT ATCCAAGATG TAGCATCAAG
3421 AATCCAATGT TTACGGGAAA AACTATGGAA GTATTATGTG AGCTCAGCAA GAAGCAGATC
3481 AATATGCGGC ACATATGCAA CCTATGTTCA AAAATGAAGA ATGTACAGAT ACAAGATCCT
3541 ATACTGCCAG AATACGAAGA AGAATACGTA GAAATTGAAA AAGAAGAACC AGGCGAAGAA
3601 AAGAATCTTG AAGACGTAAG CACTGACGAC AACAATGAAA AGAAGAAGAT AAGGTCGGTG
3661 ATTGTGAAAG AGACATAGAG GACACATGTA AGGTGGAAAA TGTAAGGGCG GAAAGTAACC
3721 TTATCACAAA GGAATCTTAT CCCCCACTAC TTATCCTTTT ATATTTTTCC GTGTCATTTT
3781 TGCCCTTGAG TTTTCCTATA TAAGGAACCA AGTTCGGCAT TTGTGAAAAC AAGAAAAAAT
3841 TTGGTGTAAG CTATTTTCTT TGAAGTACTG AGGATACAAC TTCAGAGAAA TTTGTAAGTT
3901 TGTGATCCAT GGTGAGCAAG GGCGAGGAGC TGTTCACCGG GGTGGTGCCC ATCCTGGTCG
3961 AGCTGGACGG CGACGTAAAC GGCCACAAGT TCAGCGTGTC CGGCGAGGGC GAGGGCGATG
4021 CCACCTACGG CAAGCTGACC CTGAAGTTCA TCTGCACCAC CGGCAAGCTG CCCGTGCCCT
4081 GGCCCACCCT CGTGACCACC TTCACCTACG GCGTGCAGTG CTTCAGCCGC TACCCCGACC
4141 ACATGAAGCA GCACGACTTC TTCAAGTCCG CCATGCCCGA AGGCTACGTC CAGGAGCGCA
4201 CCATCTTCTT CAAGGACGAC GGCAACTACA AGACCCGCGC CGAGGTGAAG TTCGAGGGCG
4261 ACACCCTGGT GAACCGCATC GAGCTGAAGG GCATCGACTT CAAGGAGGAC GGCAACATCC
4321 TGGGGCACAA GCTGGAGTAC AACTACAACA GCCACAACGT CTATATCATG GCCGACAAGC
4381 AGAAGAACGG CATCAAGGTG AACTTCAAGA TCCGCCACAA CATCGAGGAC GGCAGCGTGC
4441 AGCTCGCCGA CCACTACCAG CAGAACACCC CCATCGGCGA CGGCCCCGTG CTGCTGCCCG
4501 ACAACCACTA CCTGAGCACC CAGTCCGCCC TGAGCAAAGA CCCCAACGAG AAGCGCGATC
4561 ACATGGTCCT GCTGGAGTTC GTGACCGCCG CCGGGATCAC TCACGGCATG GACGAGCTGT
4621 ACAAGTAAAG CGGCCGCCCG GCTGCAGATC GTTCAAACAT TTGGCAATAA AGTTTCTTAA
4681 GATTGAATCC TGTTGCCGGT CTTGCGATGA TTATCATATA ATTTCTGTTG AATTACGTTA
```

Supplemental Figure S4. F-SHMT08cΔ+L129A-CDS, insert size, vector, restriction enzymes, and sequenced positive clones used for cloning.

```
4801 GAGTCCCGCA ATTATACATT TAATACGCGA TAGAAAACAA AATATAGCGC GCAAACTAGG
4861 ATAAATTATC GCGCGCGGTG TCATCTATGT TACTAGATCC GATGATAAGC TGTCAAACAT
4921 GAGAATTAAT TCGTAATCAT GTCATAGCTG TTTCCTGTGT GAAATTGTTA TCCGCTCACA
4981 ATTCCACACA ACATACGAGC CGGAAGCATA AAGTGTAAAG CCTGGGGTGC CTAATGAGTG
5041 AGCTAACTCA CATTAATTGC GTTGCGCTCA CTGCCCGCTT TCCAGTCGGG AAACCTGTCG
5101 TGCCAGCTGC ATTAATGAAT CGGCCAACGC GCGGGGAGAG GCGGTTTGCG TATTGGCTAG
5161 AGCAATTCGG CGTTAATTCA GTACATTAAA AACGTCCGCA ATGTGTTATT AAGTTGTCTA
5221 AGCGTCAATT TGTTTACACC ACAATATATC CTGCCACCAG CCAGCCAACA GCTCCCCGAC
5281 CGGCAGCTCG GCACAAAATC ACCACTCGAT ACAGGCAGCC CATCAGTCCG GGACGGCGTC
5341 AGCGGGAGAG CCGTTGTAAG GCGGCAGACT TTGCTCATGT TACCGATGCT ATTCGGAAGA
5401 ACGGCAACTA AGCTGCCGGG TTTGAAACAC GGATGATCTC GCGGAGGGTA GCATGTTGAT
5461 TGTAACGATG ACAGAGCGTT GCTGCCTGTG ATCCAGATCA TGAACAATAA AACTGTCTGC
5521 TTACATAAAC AGTAATACAA GGGGTGTTAT GAGCCATATT CAACGGGAAA CGTCTTGCTC
5581 TAGGCCGCGA TTAAATTCCA ACATGGATGC TGATTTATAT GGGTATAAAT GGGCTCGCGA
5641 TAATGTCGGG CAATCAGGTG CGACAATCTA TCGATTGTAT GGGAAGCCCG ATGCGCCAGA
5701 GTTGTTTCTG AAACATGGCA AAGGTAGCGT TGCCAATGAT GTTACAGATG AGATGGTCAG
5761 ACTAAACTGG CTGACGGAAT TTATGCCTCT TCCGACCATC AAGCATTTTA TCCGTACTCC
5821 TGATGATGCA TGGTTACTCA CCACTGCGAT CCCCGGGAAA ACAGCATTCC AGGTATTAGA
5881 AGAATATCCT GATTCAGGTG AAAATATTGT TGATGCGCTG GCAGTGTTCC TGCGCCGGTT
5941 GCATTCGATT CCTGTTTGTA ATTGTCCTTT TAACAGCGAT CGCGTATTTC GTCTCGCTCA
6001 GGCGCAATCA CGAATGAATA ACGGTTTGGT TGATGCGAGT GATTTTGATG ACGAGCGTAA
6061 TGGCTGGCCT GTTGAACAAG TCTGGAAAGA AATGCATAAA CTTTTGCCAT TCTCACCGGA
6121 TTCAGTCGTC ACTCATGGTG ATTTCTCACT TGATAACCTT ATTTTTGACG AGGGGAAATT
6181 AATAGGTTGT ATTGATGTTG GACGAGTCGG AATCGCAGAC CGATACCAGG ATCTTGCCAT
6241 CCTATGGAAC TGCCTCGGTG AGTTTTCTCC TTCATTACAG AAACGGCTTT TTCAAAAATA
6301 TGGTATTGAT AATCCTGATA TGAATAAATT GCAGTTTCAT TTGATGCTCG ATGAGTTTTT
6361 CTAAGAATTA ATTCATGATC CTGCATGACC AAAATCCCTT AACGTGAGTT TTCGTTCCAC
6421 TGAGCGTCAG ACCCCGTAGA AAAGATCAAA GGATCTTCTT GAGATCCTTT TTTTCTGCGC
6481 GTAATCTGCT GCTTGCAAAC AAAAAAACCA CCGCTACCAG CGGTGGTTTG TTTGCCGGAT
6541 CAAGAGCTAC CAACTCTTTT TCCGAAGGTA ACTGGCTTCA GCAGAGCGCA GATACCAAAT
6601 ACTGTCCTTC TAGTGTAGCC GTAGTTAGGC CACCACTTCA AGAACTCTGT AGCACCGCCT
6661 ACATACCTCG CTCTGCTAAT CCTGTTACCA GTGGCTGCTG CCAGTGGCGA TAAGTCGTGT
6721 CTTACCGGGT TGGACTCAAG ACGATAGTTA CCGGATAAGG CGCAGCGGTC GGGCTGAACG
6781 GGGGGTTCGT GCACACAGCC CAGCTTGGAG CGAACGACCT ACACCGAACT GAGATACCTA
6841 CAGCGTGAGC TATGAGAAAG CGCCACGCTT CCCGAAGGGA GAAAGGCGGA CAGGTATCCG
6901 GTAAGCGGCA GGGTCGGAAC AGGAGAGCGC ACGAGGGAGC TTCCAGGGGG AAACGCCTGG
6961 TATCTTTATA GTCCTGTCGG GTTTCGCCAC CTCTGACTTG AGCGTCGATT TTTGTGATGC
7021 TCGTCAGGGG GGCGGAGCCT ATGGAAAAAC GCCAGCAACG CGGCCTTTTT ACGGTTCCTG
7081 GCCTTTTGCT GGCCTTTTGC TCACATGTTC TTTCCTGCGT TATCCCCTGA TTCTGTGGAT
7141 AACCGTATTA CCGCCTTTGA GTGAGCTGAT ACCGCTCGCC GCAGCCGAAC GACCGAGCGC
7201 AGCGAGTCAG TGAGCGAGGA AGCGGAAGAG CGCCTGATGC GGTATTTTCT CCTTACGCAT
7261 CTGTGCGGTA TTTCACACCG CATATGGTGC ACTCTCAGTA CAATCTGCTC TGATGCCGCA
7321 TAGTTAAGCC AGTATACACT CCGCTATCGC TACGTGACTG GGTCATGGCT GCGCCCCGAC
7381 ACCCGCCAAC ACCCGCTGAC GCGCCCTGAC GGGCTTGTCT GCTCCCGGCA TCCGCTTACA
7441 GACAAGCTGT GACCGTCTCC GGGAGCTGCA TGTGTCAGAG GTTTTCACCG TCATCACCGA
7501 AACGCGCGAG GCAGGGTGCC TTGATGTGGG CGCCGGCGGT CGAGTGGCGA CGGCGCGGCT
7561 TGTCCGCGCC CTGGTAGATT GCCTGGCCGT AGGCCAGCCA TTTTTGAGCG GCCAGCGGCC
7621 GCGATAGGCC GACGCGAAGC GGCGGGGCGT AGGGAGCGCA GCGACCGAAG GGTAGGCGCT
7681 TTTTGCAGCT CTTCGGCTGT GCGCTGGCCA GACAGTTATG CACAGGCCAG GCGGGTTTTA
7741 AGAGTTTTAA TAAGTTTTAA AGAGTTTTAG GCGGAAAAAT CGCCTTTTTT CTCTTTTATA
7801 TCAGTCACTT ACATGTGTGA CCGGTTCCCA ATGTACGGCT TTGGGTTCCC AATGTACGGG
7861 TTCCGGTTCC CAATGTACGG CTTTGGGTTC CCAATGTACG TGCTATCCAC AGGAAAGAGA
7921 CCTTTTCGAC CTTTTTCCCC TGCTAGGGCA ATTTGCCCTA GCATCTGCTC CGTACATTAG
7981 GAACCGGCGG ATGCTTCGCC CTCGATCAGG TTGCGGTAGC GCATGACTAG GATCGGGCCA
8041 GCCTGCCCCG CCTCCTCCTT CAAATCGTAC TCCGGCAGGT CATTTGACCC GATCAGCTTG
8101 CGCACGGTGA AACAGAACTT CTTGAACTCT CCGGCGCTGC CACTGCGTTC GTAGATCGTC
8161 TTGAACAACC ATCTGGCTTC TGCCTTGCCT GCGGCGCGGC GTGCCAGGCG GTAGAGAAAA
8221 CGGCCGATGC CGGGATCGAT CAAAAAGTAA TCGGGGTGAA CCGTCAGCAC GTCCGGGTTC
8281 TTGCCTTCTG TGATCTCGCG GTACATCCAA TCAGCTAGCT CGATCTCGAT GTACTCCGGC
8341 CGCCCGGTTT CGCTCTTTAC GATCTTGTAG CGGCTAATCA AGGCTTCACC CTCGGATACC
8401 GTCACCAGGC GGCCGTTCTT GGCCTTCTTC GTACGCTGCA TGGCAACGTG CGTGGTGTTT
8461 AACCGAATGC AGGTTTCTAC CAGGTCGTCT TTCTGCTTTC CGCCATCGGC TCGCCGGCAG
8521 AACTTGAGTA CGTCCGCAAC GTGTGGACGG AACACGCGGC CGGGCTTGTC TCCCTTCCCT
8581 TCCCGGTATC GGTTCATGGA TTCGGTTAGA TGGGAAACCG CCATCAGTAC CAGGTCGTAA
8641 TCCCACACAC TGGCCATGCC GGCCGGCCCT GCGGAAACCT CTACGTGCCC GTCTGGAAGC
8701 TCGTAGCGGA TCACCTCGCC AGCTCGTCGG TCACGCTTCG ACAGACGGAA AACGGCCACG
8761 TCCATGATGC TGCGACTATC GCGGGTGCCC ACGTCATAGA GCATCGGAAC GAAAAAATCT
8821 GGTTGCTCGT CGCCCTTGGG CGGCTTCCTA ATCGACGGCG CACCGGCTGC CGGCGGTTGC
8881 CGGGATTCTT TGCGGATTCG ATCAGCGGCC GCTTGCCACG ATTCACCGGG GCGTGCTTCT
8941 GCCTCGATGC GTTGCCGCTG GGCGGCCTGC GCGGCCTTCA ACTTCTCCAC CAGGTCATCA
9001 CCCAGCGCCG CGCCGATTTG TACCGGGCCG GATGGTTTGC GACCGTCACG CCGATTCCTC
9061 GGGCTTGGGG GTTCCAGTGC CATTGCAGGG CCGGCAGACA ACCCAGCCGC TTACGCCTGG
9121 CCAACCGCCC GTTCCTCCAC ACATGGGGCA TTCCACGGCG TCGGTGCCTG GTTGTTCTTG
9181 ATTTTCCATG CCGCCTCCTT TAGCCGCTAA AATTCATCTA CTCATTTATT CATTTGCTCA
9241 TTTACTCTGG TAGCTGCGCG ATGTATTCAG ATAGCAGCTC GGTAATGGTC TTGCCTTGGC
9301 GTACCGCGTA CATCTTCAGC TTGGTGTGAT CCTCCGCCGG CAACTGAAAG TTGACCCGCT
9361 TCATGGCTGG CGTGTCTGCC AGGCTGGCCA ACGTTGCAGC CTTGCTGCTG CGTGCGCTCG
9421 GACGGCCGGC ACTTAGCGTG TTTGTGCTTT TGCTCATTTT CTCTTTACCT CATTAACTCA
9481 AATGAGTTTT GATTTAATTT CAGCGGCCAG CGCCTGGACC TCGCGGGCAG CGTCGCCCTC
9541 GGGTTCTGAT TCAAGAACGG TTGTGCCGGC GGCGGCAGTG CCTGGGTAGC TCACGCGCTG
9601 CGTGATACGG GACTCAAGAA TGGGCAGCTC GTACCCGGCC AGCGCCTCGG CAACCTCACC
9661 GCCGATGCGC GTGCCTTTGA TCGCCCGCGA CACGACAAAG GCCGCTTGTA GCCTTCCATC
9721 CGTGACCTCA ATGCGCTGCT TAACCAGCTC CACCAGGTCG GCGGTGGCCC ATATGTCGTA
9781 AGGGCTTGGC TGCACCGGAA TCAGCACGAA GTCGGCTGCC TTGATCGCGG ACACAGCCAA
9841 GTCCGCCGCC TGGGGCGCTC CGTCGATCAC TACGAAGTCG CGCCGGCCGA TGGCCTTCAC
9901 GTCGCGGTCA ATCGTCGGGC GGTCGATGCC GACAACGGTT AGCGGTTGAT CTTCCCGCAC
9961 GGCCGCCCAA TCGCGGGCAC TGCCCTGGGG ATCGGAATCG ACTAACAGAA CATCGGCCCC
10021 GGCGAGTTGC AGGGCGCGGG CTAGATGGGT TGCGATGGTC GTCTTGCCTG ACCCGCCTTT
10081 CTGGTTAAGT ACAGCGATAA CCTTCATGCG TTCCCCTTGC GTATTTGTTT ATTTACTCAT
10141 CGCATCATAT ACGCAGCGAC CGCATGACGC AAGCTGTTTT ACTCAAATAC ACATCACCTT
10201 TTTAGACGGC GGCGCTCGGT TTCTTCAGCG GCCAAGCTGG CCGGCCAGGC CGCCAGCTTG
10261 GCATCAGACA AACCGGCCAG GATTTCATGC AGCCGCACGG TTGAGACGTG CGCGGGCGGC
10321 TCGAACACGT ACCCGGCCGC GATCATCTCC GCCTCGATCT CTTCGGTAAT GAAAAACGGT
10381 TCGTCCTGGC CGTCCTGGTG CGGTTTCATG CTTGTTCCTC TTGGCGTTCA TTCTCGGCGG
10441 CCGCCAGGGC GTCGGCCTCG GTCAATGCGT CCTCACGGAA GGCACCGCGC CGCCTGGCCT
10501 CGGTGGGCGT CACTTCCTCG CTGCGCTCAA GTGCGCGGTA CAGGGTCGAG CGATGCACGC
10561 CAAGCAGTGC AGCCGCCTCT TTCACGGTGC GGCCTTCCTG GTCGATCAGC TCGCGGGCGT
10621 GCGCGATCTG TGCCGGGGTG AGGGTAGGGC GGGGGCCAAA CTTCACGCCT CGGGCCTTGG
10681 CGGCCTCGCG CCCGCTCCGG GTGCGGTCGA TGATTAGGGA ACGCTCGAAC TCGGCAATGC
10741 CGGCGAACAC GGTCAACACC ATGCGGCCGG CCGGCGTGGT GGTGTCGGCC CACGGCTCTG
10801 CCAGGCTACG CAGGCCCGCG CCGGCCTCCT GGATGCGCTC GGCAATGTCC AGTAGGTCGC
10861 GGGTGCTGCG GGCCAGGCGG TCTAGCCTGG TCACTGTCAC AACGTCGCCA GGGCGTAGGT
10921 GGTCAAGCAT CCTGGCCAGC TCCGGGCGGT CGCGCCTGGT GCCGGTGATC TTCTCGGAAA
10981 ACAGCTTGGT GCAGCCGGCC GCGTGCAGTT CGGCCCGTTG GTTGGTCAAG TCCTGGTCGT
11041 CGGTGCTGAC GCGGGCATAG CCCAGCAGGC CAGCGGCGGC GCTCTTGTTC ATGGCGTAAT
11101 GTCTCCGGTT CTAGTCGCAA GTATTCTACT TTATGCGACT AAAACACGCG ACAAGAAAAC
11161 GCCAGGAAAA GGGCAGGGCG GCAGCCTGTC GCGTAACTTA GGACTTGTGC GACATGTCGT
11221 TTTCAGAAGA CGGCTGCACT GAACGTCAGA AGCCGACTGC ACTATAGCAG CGGAGGGGTT
11281 GGATCAAAGT ACT
```

Supplemental Figure S4. F-SHMT08cΔ+L129A-CDS, insert size, vector, restriction enzymes, and sequenced positive clones used for cloning.

Gene Name F-SHMT08cΔ+L135A-CDS
Cloning Vector pG2RNAi2
Length (bp) 1430
Cloning Sites AscI-AvrII

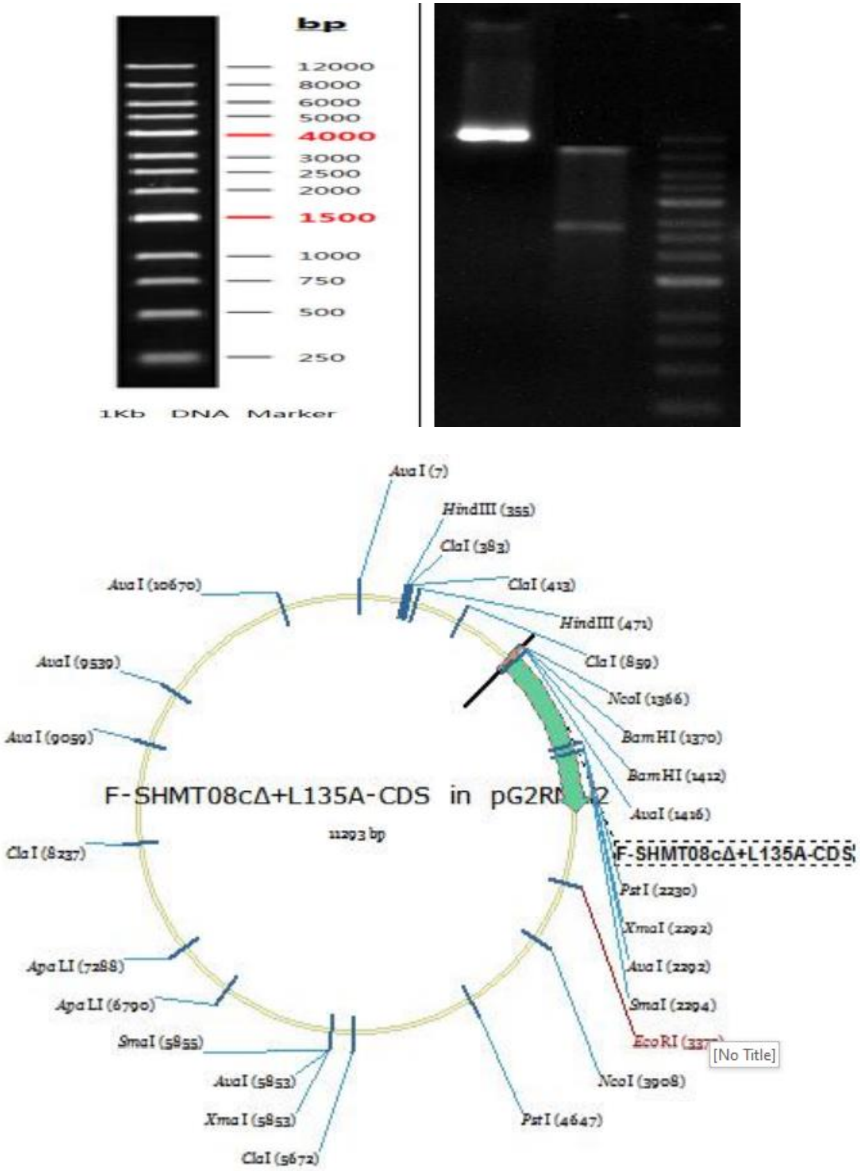

Supplemental Figure S5. F-SHMT08cΔ+L135A-CDS, insert size, vector, restriction enzymes, and sequenced positive clones used for cloning.

Detailed Sequence of the Whole Construct:

```
   1 TTGATCCCGA GGGGAACCCT GTGGTTGGCA TGCACATACA AATGGACGAA CGGATAAACC
  61 TTTTCACGCC CTTTTAAATA TCCGTTATTC TAATAAACGC TCTTTTCTCT TAGGTTTACC
 121 CGCCAATATA TCCTGTCAAA CACTGATAGT TTAAACTGAA GGCGGGAAAC GACAATCTGA
 181 TCCAAGCTCA AGCTCATTCG CCATTCAGGC TGCGCAACTG TTGGGAAGGG CGATCGGTGC
 241 GGGCCTCTTC GCTATTACGC CAGCTGGCGA AAGGGGGATG TGCTGCAAGG CGATTAAGTT
 301 GGGTAACGCC AGGGTTTTCC CAGTCACGAC GTTGTAAAAC GACGGCCAGT GCCAAGCTTA
 361 TTACCCTGTT ATCCCTAGAT ATCGATTTTG GCTACCTTAA GGTAGCCAAA ATCGATCACT
 421 AGTGCGGCCG CGGGCCCAAT ATAACAACGA CGTCGTAACA GATAAAGCGA AGCTTGAAGG
 481 TGCATGTGAC TCCGTCAAGA TTACGAAACC GCCAACTACC ACGCAAATTG CAATTCTCAA
 541 TTTCCTAGAA GGACTCTCCG AAAATGCATC CAATACCAAA TATTACCCGT GTCATAGGCA
 601 CCAAGTGACA CCATACATGA ACACGCGTCA CAATATGACT GGAGAAGGGT TCCACACCTT
 661 ATGCTATAAA ACGCCCCACA CCCCTCCTCC TTCCTTCGCA GTTCAATTCC AATATATTCC
 721 ATTCTCTCTG TGTATTTCCC TACCTCTCCC TTCAAGGTTA GTCGATTTCT TCTGTTTTTC
 781 TTCTTCGTTC TTTCCATGAA TTGTGTATGT TCTTTGATCA ATACGATGTT GATTTGATTG
 841 TGTTTTGTTT GGTTTCATCG ATCTTCAATT TTCATAATCA GATTCAGCTT TTATTATCTT
 901 TACAACAACG TCCTTAATTT GATGATTCTT TAATCGTAGA TTTGCTCTAA TTAGAGCTTT
 961 TTCATGTCAG ATCCCTTTAC AACAAGCCTT AATTGTTGAT TCATTAATCG TAGATTAGGG
1021 CTTTTTTCAT TGATTACTTC AGATCCGTTA AACGTAACCA TAGATCAGGG CTTTTTCATG
1081 AATTACTTCA GATCCGTTAA ACAACAGCCT TATTTTTTAT ACTTCTGTGG TTTTTCAAGA
1141 AATTGTTCAG ATCCGTTGAC AAAAAGCCTT ATTCGTTGAT TCTATATCGT TTTTCGAGAG
1201 ATATTGCTCA GATCTGTTAG CAACTGCCTT GTTTGTTGAT TCTATTGCCG TGGATTAGGG
1261 TTTTTTTTCA CGAGATTGCT TCAGATCCGT ACTTAAGATT ACGTAATGGA TTTTGATTCT
1321 GATTTATCTG TGATTGTTGA CTCGACAGCG GCCGCACCGG CGCGCCATGG ATCCAGTAAG
1381 CGTGTGGGGT AACACGCCCT TGGCGACGGT GGATCCCGAG ATCCATGACC TCATCGAGAA
1441 GGAGAAGCGC CGTCAATGCC GCGGAATCGA GCTCATCGCC TCCGAGAACT TCACCTCCTT
1501 CGCCGTCATC GAGGCCCTCG GCAGCGCTCT CACGAACAAA TACTCCGAGG GCATGCCGGG
```

Supplemental Figure S5. F-SHMT08cΔ+L135A-CDS, insert size, vector, restriction enzymes, and sequenced positive clones used for cloning.

```
1561 CAACCGCTAC TACGGCGGCA ATGAATACAT CGACCAGATC GAAAACCTCT GCCGCTCACG
1621 CGCCCTCCAA GCCTTCCACC TCGACGCCCA ATCCTGGGGC GTCAACGTCC AGCCCTACTC
1681 CGGCTCCCCG GCCAACTTCG CCGCCTACAC CGCCGTCCTC AACCCCCACG ACCGCATCAT
1741 GGGGCTAGAT CTCCGCTCCG GCGGCCACGC TACCCACGGC TACTACACCT CCGGCGGAAA
1801 GAAGATCTCC GCCACCTCCA TTTACTTCGA GAGTCTCCCT TACAAGGTAA ACTCCACCAC
1861 CGGCTACATC GACTACGACC GCTTGGAAGA AAAAGCCCTA GACTTCAGGC CAAAACTCAT
1921 AATCTGCGGT GGCAGCGCGT ACCCTCGCGA TTGGGACTAC AAACGTTTCA GGGAAGTCGC
1981 TGATAAGTGC GGAGCATTGC TTCTCTGCGA CATGGCGCAC ACTAGCGGCC TTGTGGCCGC
2041 GCAGGAAGTG AACAGCCCCT TCGAGTATTG CGACATTGTG ACCACCACGA CTCACAAGAG
2101 CTTGCGGGGC CCACGTGCGG GGATGATCTT TTACCGGAAG GGCCCCAAGC CGCCGAAGAA
2161 GGGGCAGCCG GAGAACGCGG TTTATGATTT CGAGGACAAG ATTAACTTCG CGGTGTTCCC
2221 TTCGCTGCAG GGTGGGCCCC ACAACCACCA GATCGGTGCT CTCGCCGTGG CGCTGAAGCA
2281 GGCCGCGTCG CCCGGGTTTA AGGCCTACGC GAAGCAGGTT AAGGCGAACG CCGTTGCGCT
2341 TGGAAAATAC TTGATGGGGA AAGGGTACAG CCTTGTCACT GGCGGAACGG AGAACCATCT
2401 TGTTTTGTGG GATCTGAGAC CTCTTGGATT GACTGGGTAT AAGGTGGAGA AACTCTGTGA
2461 TCTCTGTAAC ATTACTGTTA ACAAGAACGC TGTTTTTGGT GATAGCAGTG CCTTGGCCCC
2521 TGGTGGAGTG CGAATTGGTG CCCCTGCCAT GACTTCTAGG GGTTTGGTTG AAAAAGACTT
2581 TGAGCAGATT GGTGAGTTCC TTCACCGTGC TGTGACTCTC ACACTGGAGA TCCAGAAGGA
2641 GCATGGCAAA CTTCTCAAGG ATTTCAACAA GGGTCTCGTC AACAACAAGG CTATTGAAGA
2701 TCTCAAAGCT GATGTTGAGA AGTTCTCTGC CTTGTTTGAC ATGCCTGGCT TCCTGGTATC
2761 TGAAATGAAG TACAAGGATT AGCCTAGGTT CGAGTATTAT GGCATTGGGA AAACTGTTTT
2821 TCTTGTACCA TTTGTTGTGC TTGTAATTTA CTGTGTTTTT TATTCGGTTT TCGCTATCGA
2881 ACTGTGAAAT GGAAATGGAT GGAGAAGAGT TAATGAATGA TATGGTCCTT TTGTTCATTC
2941 TCAAATTAAT ATTATTTGTT TTTTCTCTTA TTTGTTGTGT GTTGAATTTG AAATTATAAG
3001 AGATATGCAA ACATTTTGTT TTGAGTAAAA ATGTGTCAAA TCGTGGCCTC TAATGACCGA
3061 AGTTAATATG AGGAGTAAAA CACTTGTAGT TGTACCATTA TGCTTATTCA CTAGGCAACA
3121 AATATATTTT CAGACCTAGA AAAGCTGCAA ATGTTACTGA ATACAAGTAT GTCCTCTTGT
3181 GTTTTAGACA TTTATGAACT TTCCTTTATG TAATTTTCCA GAATCCTTGT CAGATTCTAA
3241 TCATTGCTTT ATAATTATAG TTATACTCAT GGATTTGTAG TTGAGTATGA AAATATTTTT
3301 TAATGCATTT TATGACTTGC CAATTGATTG ACAACATGCA TCAATCCGCG GTTATGACTC
3361 TCTTAAGAGA GTCATGAATT CGAGCTTCCA GAAGGTAATT ATCCAAGATG TAGCATCAAG
3421 AATCCAATGT TTACGGGAAA AACTATGGAA GTATTATGTG AGCTCAGCAA GAAGCAGATC
3481 AATATGCGGC ACATATGCAA CCTATGTTCA AAAATGAAGA ATGTACAGAT ACAAGATCCT
3541 ATACTGCCAG AATACGAAGA AGAATACGTA GAAATTGAAA AAGAAGAACC AGGCGAAGAA
3601 AAGAATCTTG AAGACGTAAG CACTGACGAC AACAATGAAA AGAAGAAGAT AAGGTCGGTG
3661 ATTGTGAAAG AGACATAGAG GACACATGTA AGGTGGAAAA TGTAAGGGCG GAAAGTAACC
3721 TTATCACAAA GGAATCTTAT CCCCCACTAC TTATCCTTTT ATATTTTTCC GTGTCATTTT
3781 TGCCCTTGAG TTTTCCTATA TAAGGAACCA AGTTCGGCAT TTGTGAAAAC AAGAAAAAAT
3841 TTGGTGTAAG CTATTTTCTT TGAAGTACTG AGGATACAAC TTCAGAGAAA TTTGTAAGTT
3901 TGTGATCCAT GGTGAGCAAG GGCGAGGAGC TGTTCACCGG GGTGGTGCCC ATCCTGGTCG
3961 AGCTGGACGG CGACGTAAAC GGCCACAAGT TCAGCGTGTC CGGCGAGGGC GAGGGCGATG
4021 CCACCTACGG CAAGCTGACC CTGAAGTTCA TCTGCACCAC CGGCAAGCTG CCCGTGCCCT
4081 GGCCCACCCT CGTGACCACC TTCACCTACG GCGTGCAGTG CTTCAGCCGC TACCCCGACC
4141 ACATGAAGCA GCACGACTTC TTCAAGTCCG CCATGCCCGA AGGCTACGTC CAGGAGCGCA
4201 CCATCTTCTT CAAGGACGAC GGCAACTACA AGACCCGCGC CGAGGTGAAG TTCGAGGGCG
4261 ACACCCTGGT GAACCGCATC GAGCTGAAGG GCATCGACTT CAAGGAGGAC GGCAACATCC
4321 TGGGGCACAA GCTGGAGTAC AACTACAACA GCCACAACGT CTATATCATG GCCGACAAGC
4381 AGAAGAACGG CATCAAGGTG AACTTCAAGA TCCGCCACAA CATCGAGGAC GGCAGCGTGC
4441 AGCTCGCCGA CCACTACCAG CAGAACACCC CCATCGGCGA CGGCCCCGTG CTGCTGCCCG
4501 ACAACCACTA CCTGAGCACC CAGTCCGCCC TGAGCAAAGA CCCCAACGAG AAGCGCGATC
4561 ACATGGTCCT GCTGGAGTTC GTGACCGCCG CCGGGATCAC TCACGGCATG GACGAGCTGT
4621 ACAAGTAAAG CGGCCGCCCG GCTGCAGATC GTTCAAACAT TTGGCAATAA AGTTTCTTAA
4681 GATTGAATCC TGTTGCCGGT CTTGCGATGA TTATCATATA ATTTCTGTTG AATTACGTTA
4741 AGCATGTAAT AATTAACATG TAATGCATGA CGTTATTTAT GAGATGGGTT TTTATGATTA
```

Supplemental Figure S5. F-SHMT08cΔ+L135A-CDS, insert size, vector, restriction enzymes, and sequenced positive clones used for cloning.

```
4801 GAGTCCCGCA ATTATACATT TAATACGCGA TAGAAAACAA AATATAGCGC GCAAACTAGG
4861 ATAAATTATC GCGCGCGGTG TCATCTATGT TACTAGATCC GATGATAAGC TGTCAAACAT
4921 GAGAATTAAT TCGTAATCAT GTCATAGCTG TTTCCTGTGT GAAATTGTTA TCCGCTCACA
4981 ATTCCACACA ACATACGAGC CGGAAGCATA AAGTGTAAAG CCTGGGGTGC CTAATGAGTG
5041 AGCTAACTCA CATTAATTGC GTTGCGCTCA CTGCCCGCTT TCCAGTCGGG AAACCTGTCG
5101 TGCCAGCTGC ATTAATGAAT CGGCCAACGC GCGGGGAGAG GCGGTTTGCG TATTGGCTAG
5161 AGCAATTCGG CGTTAATTCA GTACATTAAA AACGTCCGCA ATGTGTTATT AAGTTGTCTA
5221 AGCGTCAATT TGTTTACACC ACAATATATC CTGCCACCAG CCAGCCAACA GCTCCCCGAC
5281 CGGCAGCTCG GCACAAAATC ACCACTCGAT ACAGGCAGCC CATCAGTCCG GGACGGCGTC
5341 AGCGGGAGAG CCGTTGTAAG GCGGCAGACT TTGCTCATGT TACCGATGCT ATTCGGAAGA
5401 ACGGCAACTA AGCTGCCGGG TTTGAAACAC GGATGATCTC GCGGAGGGTA GCATGTTGAT
5461 TGTAACGATG ACAGAGCGTT GCTGCCTGTG ATCCAGATCA TGAACAATAA AACTGTCTGC
5521 TTACATAAAC AGTAATACAA GGGGTGTTAT GAGCCATATT CAACGGGAAA CGTCTTGCTC
5581 TAGGCCGCGA TTAAATTCCA ACATGGATGC TGATTTATAT GGGTATAAAT GGGCTCGCGA
5641 TAATGTCGGG CAATCAGGTG CGACAATCTA TCGATTGTAT GGGAAGCCCG ATGCGCCAGA
5701 GTTGTTTCTG AAACATGGCA AAGGTAGCGT TGCCAATGAT GTTACAGATG AGATGGTCAG
5761 ACTAAACTGG CTGACGGAAT TTATGCCTCT TCCGACCATC AAGCATTTTA TCCGTACTCC
5821 TGATGATGCA TGGTTACTCA CCACTGCGAT CCCCGGGAAA ACAGCATTCC AGGTATTAGA
5881 AGAATATCCT GATTCAGGTG AAAATATTGT TGATGCGCTG GCAGTGTTCC TGCGCCGGTT
5941 GCATTCGATT CCTGTTTGTA ATTGTCCTTT TAACAGCGAT CGCGTATTTC GTCTCGCTCA
6001 GGCGCAATCA CGAATGAATA ACGGTTTGGT TGATGCGAGT GATTTTGATG ACGAGCGTAA
6061 TGGCTGGCCT GTTGAACAAG TCTGGAAAGA AATGCATAAA CTTTTGCCAT TCTCACCGGA
6121 TTCAGTCGTC ACTCATGGTG ATTTCTCACT TGATAACCTT ATTTTTGACG AGGGGAAATT
6181 AATAGGTTGT ATTGATGTTG GACGAGTCGG AATCGCAGAC CGATACCAGG ATCTTGCCAT
6241 CCTATGGAAC TGCCTCGGTG AGTTTTCTCC TTCATTACAG AAACGGCTTT TTCAAAAATA
6301 TGGTATTGAT AATCCTGATA TGAATAAATT GCAGTTTCAT TTGATGCTCG ATGAGTTTTT
6361 CTAAGAATTA ATTCATGATC CTGCATGACC AAAATCCCTT AACGTGAGTT TTCGTTCCAC
6421 TGAGCGTCAG ACCCCGTAGA AAAGATCAAA GGATCTTCTT GAGATCCTTT TTTTCTGCGC
6481 GTAATCTGCT GCTTGCAAAC AAAAAAACCA CCGCTACCAG CGGTGGTTTG TTTGCCGGAT
6541 CAAGAGCTAC CAACTCTTTT TCCGAAGGTA ACTGGCTTCA GCAGAGCGCA GATACCAAAT
6601 ACTGTCCTTC TAGTGTAGCC GTAGTTAGGC CACCACTTCA AGAACTCTGT AGCACCGCCT
6661 ACATACCTCG CTCTGCTAAT CCTGTTACCA GTGGCTGCTG CCAGTGGCGA TAAGTCGTGT
6721 CTTACCGGGT TGGACTCAAG ACGATAGTTA CCGGATAAGG CGCAGCGGTC GGGCTGAACG
6781 GGGGGTTCGT GCACACAGCC CAGCTTGGAG CGAACGACCT ACACCGAACT GAGATACCTA
6841 CAGCGTGAGC TATGAGAAAG CGCCACGCTT CCCGAAGGGA GAAAGGCGGA CAGGTATCCG
6901 GTAAGCGGCA GGGTCGGAAC AGGAGAGCGC ACGAGGGAGC TTCCAGGGGG AAACGCCTGG
6961 TATCTTTATA GTCCTGTCGG GTTTCGCCAC CTCTGACTTG AGCGTCGATT TTTGTGATGC
7021 TCGTCAGGGG GGCGGAGCCT ATGGAAAAAC GCCAGCAACG CGGCCTTTTT ACGGTTCCTG
7081 GCCTTTTGCT GGCCTTTTGC TCACATGTTC TTTCCTGCGT TATCCCCTGA TTCTGTGGAT
7141 AACCGTATTA CCGCCTTTGA GTGAGCTGAT ACCGCTCGCC GCAGCCGAAC GACCGAGCGC
7201 AGCGAGTCAG TGAGCGAGGA AGCGGAAGAG CGCCTGATGC GGTATTTTCT CCTTACGCAT
7261 CTGTGCGGTA TTTCACACCG CATATGGTGC ACTCTCAGTA CAATCTGCTC TGATGCCGCA
7321 TAGTTAAGCC AGTATACACT CCGCTATCGC TACGTGACTG GGTCATGGCT GCGCCCCGAC
7381 ACCCGCCAAC ACCCGCTGAC GCGCCCTGAC GGGCTTGTCT GCTCCCGGCA TCCGCTTACA
7441 GACAAGCTGT GACCGTCTCC GGGAGCTGCA TGTGTCAGAG GTTTTCACCG TCATCACCGA
7501 AACGCGCGAG GCAGGGTGCC TTGATGTGGG CGCCGGCGGT CGAGTGGCGA CGGCGCGGCT
7561 TGTCCGCGCC CTGGTAGATT GCCTGGCCGT AGGCCAGCCA TTTTTGAGCG GCCAGCGGCC
7621 GCGATAGGCC GACGCGAAGC GGCGGGGCGT AGGGAGCGCA GCGACCGAAG GGTAGGCGCT
7681 TTTTGCAGCT CTTCGGCTGT GCGCTGGCCA GACAGTTATG CACAGGCCAG GCGGGTTTTA
7741 AGAGTTTTAA TAAGTTTTAA AGAGTTTTAG GCGGAAAAAT CGCCTTTTTT CTCTTTTATA
7801 TCAGTCACTT ACATGTGTGA CCGGTTCCCA ATGTACGGCT TTGGGTTCCC AATGTACGGG
7861 TTCCGGTTCC CAATGTACGG CTTTGGGTTC CCAATGTACG TGCTATCCAC AGGAAAGAGA
7921 CCTTTTCGAC CTTTTTCCCC TGCTAGGGCA ATTTGCCCTA GCATCTGCTC CGTACATTAG
7981 GAACCGGCGG ATGCTTCGCC CTCGATCAGG TTGCGGTAGC GCATGACTAG GATCGGGCCA
8041 GCCTGCCCCG CCTCCTCCTT CAAATCGTAC TCCGGCAGGT CATTTGACCC GATCAGCTTG
8101 CGCACGGTGA AACAGAACTT CTTGAACTCT CCGGCGCTGC CACTGCGTTC GTAGATCGTC
8161 TTGAACAACC ATCTGGCTTC TGCCTTGCCT GCGGCGCGGC GTGCCAGGCG GTAGAGAAAA
8221 CGGCCGATGC CGGGATCGAT CAAAAAGTAA TCGGGGTGAA CCGTCAGCAC GTCCGGGTTC
8281 TTGCCTTCTG TGATCTCGCG GTACATCCAA TCAGCTAGCT CGATCTCGAT GTACTCCGGC
8341 CGCCCGGTTT CGCTCTTTAC GATCTTGTAG CGGCTAATCA AGGCTTCACC CTCGGATACC
8401 GTCACCAGGC GGCCGTTCTT GGCCTTCTTC GTACGCTGCA TGGCAACGTG CGTGGTGTTT
8461 AACCGAATGC AGGTTTCTAC CAGGTCGTCT TTCTGCTTTC CGCCATCGGC TCGCCGGCAG
8521 AACTTGAGTA CGTCCGCAAC GTGTGGACGG AACACGCGGC CGGGCTTGTC TCCCTTCCCT
8581 TCCCGGTATC GGTTCATGGA TTCGGTTAGA TGGGAAACCG CCATCAGTAC CAGGTCGTAA
8641 TCCCACACAC TGGCCATGCC GGCCGGCCCT GCGGAAACCT CTACGTGCCC GTCTGGAAGC
8701 TCGTAGCGGA TCACCTCGCC AGCTCGTCGG TCACGCTTCG ACAGACGGAA AACGGCCACG
8761 TCCATGATGC TGCGACTATC GCGGGTGCCC ACGTCATAGA GCATCGGAAC GAAAAAATCT
8821 GGTTGCTCGT CGCCCTTGGG CGGCTTCCTA ATCGACGGCG CACCGGCTGC CGGCGGTTGC
8881 CGGGATTCTT TGCGGATTCG ATCAGCGGCC GCTTGCCACG ATTCACCGGG GCGTGCTTCT
8941 GCCTCGATGC GTTGCCGCTG GGCGGCCTGC GCGGCCTTCA ACTTCTCCAC CAGGTCATCA
9001 CCCAGCGCCG CGCCGATTTG TACCGGGCCG GATGGTTTGC GACCGTCACG CCGATTCCTC
9061 GGGCTTGGGG GTTCCAGTGC CATTGCAGGG CCGGCAGACA ACCCAGCCGC TTACGCCTGG
9121 CCAACCGCCC GTTCCTCCAC ACATGGGGCA TTCCACGGCG TCGGTGCCTG GTTGTTCTTG
9181 ATTTTCCATG CCGCCTCCTT TAGCCGCTAA AATTCATCTA CTCATTTATT CATTTGCTCA
9241 TTTACTCTGG TAGCTGCGCG ATGTATTCAG ATAGCAGCTC GGTAATGGTC TTGCCTTGGC
9301 GTACCGCGTA CATCTTCAGC TTGGTGTGAT CCTCCGCCGG CAACTGAAAG TTGACCCGCT
9361 TCATGGCTGG CGTGTCTGCC AGGCTGGCCA ACGTTGCAGC CTTGCTGCTG CGTGCGCTCG
9421 GACGGCCGGC ACTTAGCGTG TTTGTGCTTT TGCTCATTTT CTCTTTACCT CATTAACTCA
9481 AATGAGTTTT GATTTAATTT CAGCGGCCAG CGCCTGGACC TCGCGGGCAG CGTCGCCCTC
9541 GGGTTCTGAT TCAAGAACGG TTGTGCCGGC GGCGGCAGTG CCTGGGTAGC TCACGCGCTG
9601 CGTGATACGG GACTCAAGAA TGGGCAGCTC GTACCCGGCC AGCGCCTCGG CAACCTCACC
9661 GCCGATGCGC GTGCCTTTGA TCGCCCGCGA CACGACAAAG GCCGCTTGTA GCCTTCCATC
9721 CGTGACCTCA ATGCGCTGCT TAACCAGCTC CACCAGGTCG GCGGTGGCCC ATATGTCGTA
9781 AGGGCTTGGC TGCACCGGAA TCAGCACGAA GTCGGCTGCC TTGATCGCGG ACACAGCCAA
9841 GTCCGCCGCC TGGGGCGCTC CGTCGATCAC TACGAAGTCG CGCCGGCCGA TGGCCTTCAC
9901 GTCGCGGTCA ATCGTCGGGC GGTCGATGCC GACAACGGTT AGCGGTTGAT CTTCCCGCAC
9961 GGCCGCCCAA TCGCGGGCAC TGCCCTGGGG ATCGGAATCG ACTAACAGAA CATCGGCCCC
10021 GGCGAGTTGC AGGGCGCGGG CTAGATGGGT TGCGATGGTC GTCTTGCCTG ACCCGCCTTT
10081 CTGGTTAAGT ACAGCGATAA CCTTCATGCG TTCCCCTTGC GTATTTGTTT ATTTACTCAT
10141 CGCATCATAT ACGCAGCGAC CGCATGACGC AAGCTGTTTT ACTCAAATAC ACATCACCTT
10201 TTTAGACGGC GGCGCTCGGT TTCTTCAGCG GCCAAGCTGG CCGGCCAGGC CGCCAGCTTG
10261 GCATCAGACA AACCGGCCAG GATTTCATGC AGCCGCACGG TTGAGACGTG CGCGGGCGGC
10321 TCGAACACGT ACCCGGCCGC GATCATCTCC GCCTCGATCT CTTCGGTAAT GAAAAACGGT
10381 TCGTCCTGGC CGTCCTGGTG CGGTTTCATG CTTGTTCCTC TTGGCGTTCA TTCTCGGCGG
10441 CCGCCAGGGC GTCGGCCTCG GTCAATGCGT CCTCACGGAA GGCACCGCGC CGCCTGGCCT
10501 CGGTGGGCGT CACTTCCTCG CTGCGCTCAA GTGCGCGGTA CAGGGTCGAG CGATGCACGC
10561 CAAGCAGTGC AGCCGCCTCT TTCACGGTGC GGCCTTCCTG GTCGATCAGC TCGCGGGCGT
10621 GCGCGATCTG TGCCGGGGTG AGGGTAGGGC GGGGGCCAAA CTTCACGCCT CGGGCCTTGG
10681 CGGCCTCGCG CCCGCTCCGG GTGCGGTCGA TGATTAGGGA ACGCTCGAAC TCGGCAATGC
10741 CGGCGAACAC GGTCAACACC ATGCGGCCGG CCGGCGTGGT GGTGTCGGCC CACGGCTCTG
10801 CCAGGCTACG CAGGCCCGCG CCGGCCTCCT GGATGCGCTC GGCAATGTCC AGTAGGTCGC
10861 GGGTGCTGCG GGCCAGGCGG TCTAGCCTGG TCACTGTCAC AACGTCGCCA GGGCGTAGGT
10921 GGTCAAGCAT CCTGGCCAGC TCCGGGCGGT CGCGCCTGGT GCCGGTGATC TTCTCGGAAA
10981 ACAGCTTGGT GCAGCCGGCC GCGTGCAGTT CGGCCCGTTG GTTGGTCAAG TCCTGGTCGT
11041 CGGTGCTGAC GCGGGCATAG CCCAGCAGGC CAGCGGCGGC GCTCTTGTTC ATGGCGTAAT
11101 GTCTCCGGTT CTAGTCGCAA GTATTCTACT TTATGCGACT AAAACACGCG ACAAGAAAAC
11161 GCCAGGAAAA GGGCAGGGCG GCAGCCTGTC GCGTAACTTA GGACTTGTGC GACATGTCGT
11221 TTTCAGAAGA CGGCTGCACT GAACGTCAGA AGCCGACTGC ACTATAGCAG CGGAGGGGTT
11281 GGATCAAAGT ACT
```

Supplemental Figure S5. F-SHMT08cΔ+L135A-CDS, insert size, vector, restriction enzymes, and sequenced positive clones used for cloning.

Gene Name F-SHMT08cΔ+S190A-CDS
Cloning Vector pG2RNAi2
Length (bp) 1430
Cloning Sites AscI-AvrII

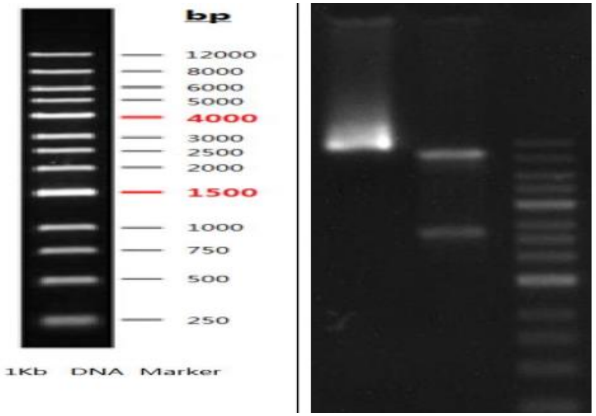

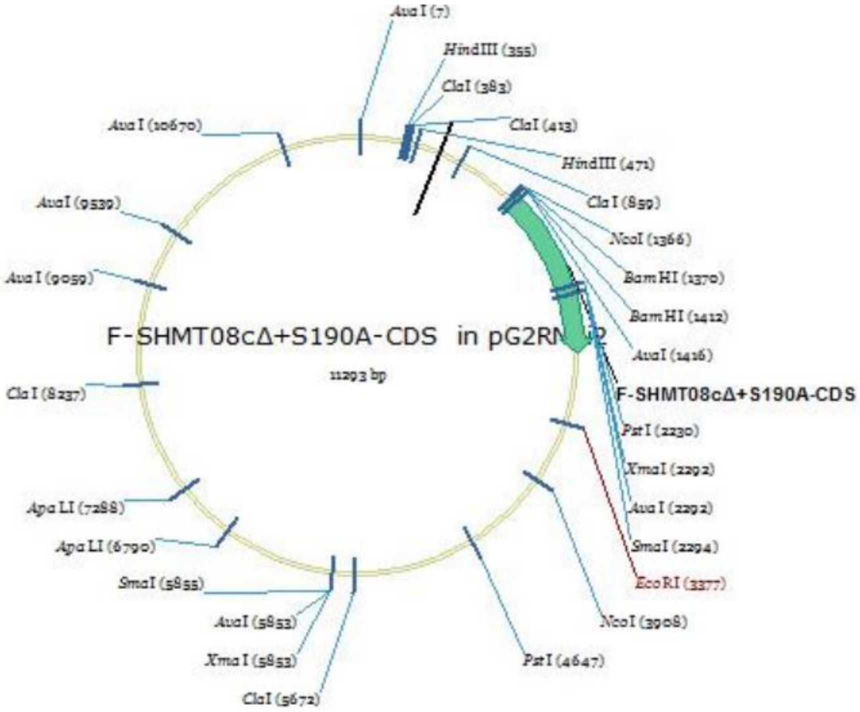

Supplemental Figure S6. F-SHMT08cΔ+S190A-CDS, insert size, vector, restriction enzymes, and sequenced positive clones used for cloning.

Detailed Sequence of the Whole Construct:

```
   1 TTGATCCCGA GGGGAACCCT GTGGTTGGCA TGCACATACA AATGGACGAA CGGATAAACC
  61 TTTTCACGCC CTTTTAAATA TCCGTTATTC TAATAAACGC TCTTTTCTCT TAGGTTTACC
 121 CGCCAATATA TCCTGTCAAA CACTGATAGT TTAAACTGAA GGCGGGAAAC GACAATCTGA
 181 TCCAAGCTCA AGCTCATTCG CCATTCAGGC TGCGCAACTG TTGGGAAGGG CGATCGGTGC
 241 GGGCCTCTTC GCTATTACGC CAGCTGGCGA AAGGGGGATG TGCTGCAAGG CGATTAAGTT
 301 GGGTAACGCC AGGGTTTTCC CAGTCACGAC GTTGTAAAAC GACGGCCAGT GCCAAGCTTA
 361 TTACCCTGTT ATCCCTAGAT ATCGATTTTG GCTACCTTAA GGTAGCCAAA ATCGATCACT
 421 AGTGCGGCCG CGGGCCCAAT ATAACAACGA CGTCGTAACA GATAAAGCGA AGCTTGAAGG
 481 TGCATGTGAC TCCGTCAAGA TTACGAAACC GCCAACTACC ACGCAAATTG CAATTCTCAA
 541 TTTCCTAGAA GGACTCTCCG AAAATGCATC CAATACCAAA TATTACCCGT GTCATAGGCA
 601 CCAAGTGACA CCATACATGA ACACGCGTCA CAATATGACT GGAGAAGGGT TCCACACCTT
 661 ATGCTATAAA ACGCCCCACA CCCCTCCTCC TTCCTTCGCA GTTCAATTCC AATATATTCC
 721 ATTCTCTCTG TGTATTTCCC TACCTCTCCC TTCAAGGTTA GTCGATTTCT TCTGTTTTTC
 781 TTCTTCGTTC TTTCCATGAA TTGTGTATGT TCTTTGATCA ATACGATGTT GATTTGATTG
 841 TGTTTTGTTT GGTTTCATCG ATCTTCAATT TTCATAATCA GATTCAGCTT TTATTATCTT
 901 TACAACAACG TCCTTAATTT GATGATTCTT TAATCGTAGA TTTGCTCTAA TTAGAGCTTT
 961 TTCATGTCAG ATCCCTTTAC AACAAGCCTT AATTGTTGAT TCATTAATCG TAGATTAGGG
1021 CTTTTTTCAT TGATTACTTC AGATCCGTTA AACGTAACCA TAGATCAGGG CTTTTTCATG
1081 AATTACTTCA GATCCGTTAA ACAACAGCCT TATTTTTTAT ACTTCTGTGG TTTTTCAAGA
1141 AATTGTTCAG ATCCGTTGAC AAAAAGCCTT ATTCGTTGAT TCTATATCGT TTTTCGAGAG
1201 ATATTGCTCA GATCTGTTAG CAACTGCCTT GTTTGTTGAT TCTATTGCCG TGGATTAGGG
1261 TTTTTTTTCA CGAGATTGCT TCAGATCCGT ACTTAAGATT ACGTAATGGA TTTTGATTCT
1321 GATTTATCTG TGATTGTTGA CTCGACAGCG GCCGCACCGG CGCGCCATGG ATCCAGTAAG
1381 CGTGTGGGGT AACACGCCCT TGGCGACGGT GGATCCCGAG ATCCATGACC TCATCGAGAA
1441 GGAGAAGCGC CGTCAATGCC GCGGAATCGA GCTCATCGCC TCCGAGAACT TCACCTCCTT
```

Supplemental Figure S6. F-SHMT08cΔ+S190A-CDS, insert size, vector, restriction enzymes, and sequenced positive clones used for cloning.

```
1501 CGCCGTCATC GAGGCCCTCG GCAGCGCTCT CACGAACAAA TACTCCGAGG GCATGCCGGG
1561 CAACCGCTAC TACGGCGGCA ATGAATACAT CGACCAGATC GAAAACCTCT GCCGCTCACG
1621 CGCCCTCCAA GCCTTCCACC TCGACGCCCA ATCCTGGGGC GTCAACGTCC AGCCCTACTC
1681 CGGCTCCCCG GCCAACTTCG CCGCCTACAC CGCCGTCCTC AACCCCCACG ACCGCATCAT
1741 GGGGCTAGAT CTCCGCTCCG GCGGCCACCT CACCCACGGC TACTACACCT CCGGCGGAAA
1801 GAAGATCTCC GCCACCTCCA TTTACTTCGA GAGTCTCCCT TACAAGGTAA ACTCCACCAC
1861 CGGCTACATC GACTACGACC GCTTGGAAGA AAAAGCCCTA GACTTCAGGC CAAAACTCAT
1921 AATCTGCGGT GGCGCTGCGT ACCCTCGCGA TTGGGACTAC AAACGTTTCA GGGAAGTCGC
1981 TGATAAGTGC GGAGCATTGC TTCTCTGCGA CATGGCGCAC ACTAGCGGCC TTGTGGCCGC
2041 GCAGGAAGTG AACAGCCCCT TCGAGTATTG CGACATTGTG ACCACCACGA CTCACAAGAG
2101 CTTGCGGGGC CCACGTGCGG GGATGATCTT TTACCGGAAG GGCCCCAAGC CGCCGAAGAA
2161 GGGGCAGCCG GAGAACGCGG TTTATGATTT CGAGGACAAG ATTAACTTCG CGGTGTTCCC
2221 TTCGCTGCAG GGTGGGCCCC ACAACCACCA GATCGGTGCT CTCGCCGTGG CGCTGAAGCA
2281 GGCCGCGTCG CCCGGGTTTA AGGCCTACGC GAAGCAGGTT AAGGCGAACG CCGTTGCGCT
2341 TGGAAAATAC TTGATGGGGA AAGGGTACAG CCTTGTCACT GGCGGAACGG AGAACCATCT
2401 TGTTTTGTGG GATCTGAGAC CTCTTGGATT GACTGGGTAT AAGGTGGAGA AACTCTGTGA
2461 TCTCTGTAAC ATTACTGTTA ACAAGAACGC TGTTTTTGGT GATAGCAGTG CCTTGGCCCC
2521 TGGTGGAGTG CGAATTGGTG CCCCTGCCAT GACTTCTAGG GGTTTGGTTG AAAAAGACTT
2581 TGAGCAGATT GGTGAGTTCC TTCACCGTGC TGTGACTCTC ACACTGGAGA TCCAGAAGGA
2641 GCATGGCAAA CTTCTCAAGG ATTTCAACAA GGGTCTCGTC AACAACAAGG CTATTGAAGA
2701 TCTCAAAGCT GATGTTGAGA AGTTCTCTGC CTTGTTTGAC ATGCCTGGCT TCCTGGTATC
2761 TGAAATGAAG TACAAGGATT AGCCTAGGTT CGAGTATTAT GGCATTGGGA AAACTGTTTT
2821 TCTTGTACCA TTTGTTGTGC TTGTAATTTA CTGTGTTTTT TATTCGGTTT TCGCTATCGA
2881 ACTGTGAAAT GGAAATGGAT GGAGAAGAGT TAATGAATGA TATGGTCCTT TTGTTCATTC
2941 TCAAATTAAT ATTATTTGTT TTTTCTCTTA TTTGTTGTGT GTTGAATTTG AAATTATAAG
3001 AGATATGCAA ACATTTTGTT TTGAGTAAAA ATGTGTCAAA TCGTGGCCTC TAATGACCGA
3061 AGTTAATATG AGGAGTAAAA CACTTGTAGT TGTACCATTA TGCTTATTCA CTAGGCAACA
3121 AATATATTTT CAGACCTAGA AAAGCTGCAA ATGTTACTGA ATACAAGTAT GTCCTCTTGT
3181 GTTTTAGACA TTTATGAACT TTCCTTTATG TAATTTTCCA GAATCCTTGT CAGATTCTAA
3241 TCATTGCTTT ATAATTATAG TTATACTCAT GGATTTGTAG TTGAGTATGA AAATATTTTT
3301 TAATGCATTT TATGACTTGC CAATTGATTG ACAACATGCA TCAATCCGCG GTTATGACTC
3361 TCTTAAGAGA GTCATGAATT CGAGCTTCCA GAAGGTAATT ATCCAAGATG TAGCATCAAG
3421 AATCCAATGT TTACGGGAAA AACTATGGAA GTATTATGTG AGCTCAGCAA GAAGCAGATC
3481 AATATGCGGC ACATATGCAA CCTATGTTCA AAAATGAAGA ATGTACAGAT ACAAGATCCT
3541 ATACTGCCAG AATACGAAGA AGAATACGTA GAAATTGAAA AAGAAGAACC AGGCGAAGAA
3601 AAGAATCTTG AAGACGTAAG CACTGACGAC AACAATGAAA AGAAGAAGAT AAGGTCGGTG
3661 ATTGTGAAAG AGACATAGAG GACACATGTA AGGTGGAAAA TGTAAGGGCG GAAAGTAACC
3721 TTATCACAAA GGAATCTTAT CCCCCACTAC TTATCCTTTT ATATTTTTCC GTGTCATTTT
3781 TGCCCTTGAG TTTTCCTATA TAAGGAACCA AGTTCGGCAT TTGTGAAAAC AAGAAAAAAT
3841 TTGGTGTAAG CTATTTTCTT TGAAGTACTG AGGATACAAC TTCAGAGAAA TTTGTAAGTT
3901 TGTGATCCAT GGTGAGCAAG GGCGAGGAGC TGTTCACCGG GGTGGTGCCC ATCCTGGTCG
3961 AGCTGGACGG CGACGTAAAC GGCCACAAGT TCAGCGTGTC CGGCGAGGGC GAGGGCGATG
4021 CCACCTACGG CAAGCTGACC CTGAAGTTCA TCTGCACCAC CGGCAAGCTG CCCGTGCCCT
4081 GGCCCACCCT CGTGACCACC TTCACCTACG GCGTGCAGTG CTTCAGCCGC TACCCCGACC
4141 ACATGAAGCA GCACGACTTC TTCAAGTCCG CCATGCCCGA AGGCTACGTC CAGGAGCGCA
4201 CCATCTTCTT CAAGGACGAC GGCAACTACA AGACCCGCGC CGAGGTGAAG TTCGAGGGCG
4261 ACACCCTGGT GAACCGCATC GAGCTGAAGG GCATCGACTT CAAGGAGGAC GGCAACATCC
4321 TGGGGCACAA GCTGGAGTAC AACTACAACA GCCACAACGT CTATATCATG GCCGACAAGC
4381 AGAAGAACGG CATCAAGGTG AACTTCAAGA TCCGCCACAA CATCGAGGAC GGCAGCGTGC
4441 AGCTCGCCGA CCACTACCAG CAGAACACCC CCATCGGCGA CGGCCCCGTG CTGCTGCCCG
4501 ACAACCACTA CCTGAGCACC CAGTCCGCCC TGAGCAAAGA CCCCAACGAG AAGCGCGATC
4561 ACATGGTCCT GCTGGAGTTC GTGACCGCCG CCGGGATCAC TCACGGCATG GACGAGCTGT
4621 ACAAGTAAAG CGGCCGCCCG GCTGCAGATC GTTCAAACAT TTGGCAATAA AGTTTCTTAA
4681 GATTGAATCC TGTTGCCGGT CTTGCGATGA TTATCATATA ATTTCTGTTG AATTACGTTA
```

Supplemental Figure S6. F-SHMT08cΔ+S190A-CDS, insert size, vector, restriction enzymes, and sequenced positive clones used for cloning.

```
4801 GAGTCCCGCA ATTATACATT TAATACGCGA TAGAAAACAA AATATAGCGC GCAAACTAGG
4861 ATAAATTATC GCGCGCGGTG TCATCTATGT TACTAGATCC GATGATAAGC TGTCAAACAT
4921 GAGAATTAAT TCGTAATCAT GTCATAGCTG TTTCCTGTGT GAAATTGTTA TCCGCTCACA
4981 ATTCCACACA ACATACGAGC CGGAAGCATA AAGTGTAAAG CCTGGGGTGC CTAATGAGTG
5041 AGCTAACTCA CATTAATTGC GTTGCGCTCA CTGCCCGCTT TCCAGTCGGG AAACCTGTCG
5101 TGCCAGCTGC ATTAATGAAT CGGCCAACGC GCGGGGAGAG GCGGTTTGCG TATTGGCTAG
5161 AGCAATTCGG CGTTAATTCA GTACATTAAA AACGTCCGCA ATGTGTTATT AAGTTGTCTA
5221 AGCGTCAATT TGTTTACACC ACAATATATC CTGCCACCAG CCAGCCAACA GCTCCCCGAC
5281 CGGCAGCTCG GCACAAAATC ACCACTCGAT ACAGGCAGCC CATCAGTCCG GGACGGCGTC
5341 AGCGGGAGAG CCGTTGTAAG GCGGCAGACT TTGCTCATGT TACCGATGCT ATTCGGAAGA
5401 ACGGCAACTA AGCTGCCGGG TTTGAAACAC GGATGATCTC GCGGAGGGTA GCATGTTGAT
5461 TGTAACGATG ACAGAGCGTT GCTGCCTGTG ATCCAGATCA TGAACAATAA AACTGTCTGC
5521 TTACATAAAC AGTAATACAA GGGGTGTTAT GAGCCATATT CAACGGGAAA CGTCTTGCTC
5581 TAGGCCGCGA TTAAATTCCA ACATGGATGC TGATTTATAT GGGTATAAAT GGGCTCGCGA
5641 TAATGTCGGG CAATCAGGTG CGACAATCTA TCGATTGTAT GGGAAGCCCG ATGCGCCAGA
5701 GTTGTTTCTG AAACATGGCA AAGGTAGCGT TGCCAATGAT GTTACAGATG AGATGGTCAG
5761 ACTAAACTGG CTGACGGAAT TTATGCCTCT TCCGACCATC AAGCATTTTA TCCGTACTCC
5821 TGATGATGCA TGGTTACTCA CCACTGCGAT CCCCGGGAAA ACAGCATTCC AGGTATTAGA
5881 AGAATATCCT GATTCAGGTG AAAATATTGT TGATGCGCTG GCAGTGTTCC TGCGCCGGTT
5941 GCATTCGATT CCTGTTTGTA ATTGTCCTTT TAACAGCGAT CGCGTATTTC GTCTCGCTCA
6001 GGCGCAATCA CGAATGAATA ACGGTTTGGT TGATGCGAGT GATTTTGATG ACGAGCGTAA
6061 TGGCTGGCCT GTTGAACAAG TCTGGAAAGA AATGCATAAA CTTTTGCCAT TCTCACCGGA
6121 TTCAGTCGTC ACTCATGGTG ATTTCTCACT TGATAACCTT ATTTTTGACG AGGGGAAATT
6181 AATAGGTTGT ATTGATGTTG GACGAGTCGG AATCGCAGAC CGATACCAGG ATCTTGCCAT
6241 CCTATGGAAC TGCCTCGGTG AGTTTTCTCC TTCATTACAG AAACGGCTTT TTCAAAAATA
6301 TGGTATTGAT AATCCTGATA TGAATAAATT GCAGTTTCAT TTGATGCTCG ATGAGTTTTT
6361 CTAAGAATTA ATTCATGATC CTGCATGACC AAAATCCCTT AACGTGAGTT TTCGTTCCAC
6421 TGAGCGTCAG ACCCCGTAGA AAAGATCAAA GGATCTTCTT GAGATCCTTT TTTTCTGCGC
6481 GTAATCTGCT GCTTGCAAAC AAAAAAACCA CCGCTACCAG CGGTGGTTTG TTTGCCGGAT
6541 CAAGAGCTAC CAACTCTTTT TCCGAAGGTA ACTGGCTTCA GCAGAGCGCA GATACCAAAT
6601 ACTGTCCTTC TAGTGTAGCC GTAGTTAGGC CACCACTTCA AGAACTCTGT AGCACCGCCT
6661 ACATACCTCG CTCTGCTAAT CCTGTTACCA GTGGCTGCTG CCAGTGGCGA TAAGTCGTGT
6721 CTTACCGGGT TGGACTCAAG ACGATAGTTA CCGGATAAGG CGCAGCGGTC GGGCTGAACG
6781 GGGGGTTCGT GCACACAGCC CAGCTTGGAG CGAACGACCT ACACCGAACT GAGATACCTA
6841 CAGCGTGAGC TATGAGAAAG CGCCACGCTT CCCGAAGGGA GAAAGGCGGA CAGGTATCCG
6901 GTAAGCGGCA GGGTCGGAAC AGGAGAGCGC ACGAGGGAGC TTCCAGGGGG AAACGCCTGG
6961 TATCTTTATA GTCCTGTCGG GTTTCGCCAC CTCTGACTTG AGCGTCGATT TTTGTGATGC
7021 TCGTCAGGGG GGCGGAGCCT ATGGAAAAAC GCCAGCAACG CGGCCTTTTT ACGGTTCCTG
7081 GCCTTTTGCT GGCCTTTTGC TCACATGTTC TTTCCTGCGT TATCCCCTGA TTCTGTGGAT
7141 AACCGTATTA CCGCCTTTGA GTGAGCTGAT ACCGCTCGCC GCAGCCGAAC GACCGAGCGC
7201 AGCGAGTCAG TGAGCGAGGA AGCGGAAGAG CGCCTGATGC GGTATTTTCT CCTTACGCAT
7261 CTGTGCGGTA TTTCACACCG CATATGGTGC ACTCTCAGTA CAATCTGCTC TGATGCCGCA
7321 TAGTTAAGCC AGTATACACT CCGCTATCGC TACGTGACTG GGTCATGGCT GCGCCCCGAC
7381 ACCCGCCAAC ACCCGCTGAC GCGCCCTGAC GGGCTTGTCT GCTCCCGGCA TCCGCTTACA
7441 GACAAGCTGT GACCGTCTCC GGGAGCTGCA TGTGTCAGAG GTTTTCACCG TCATCACCGA
7501 AACGCGCGAG GCAGGGTGCC TTGATGTGGG CGCCGGCGGT CGAGTGGCGA CGGCGCGGCT
7561 TGTCCGCGCC CTGGTAGATT GCCTGGCCGT AGGCCAGCCA TTTTTGAGCG GCCAGCGGCC
7621 GCGATAGGCC GACGCGAAGC GGCGGGGCGT AGGGAGCGCA GCGACCGAAG GGTAGGCGCT
7681 TTTTGCAGCT CTTCGGCTGT GCGCTGGCCA GACAGTTATG CACAGGCCAG GCGGGTTTTA
7741 AGAGTTTTAA TAAGTTTTAA AGAGTTTTAG GCGGAAAAAT CGCCTTTTTT CTCTTTTATA
7801 TCAGTCACTT ACATGTGTGA CCGGTTCCCA ATGTACGGCT TTGGGTTCCC AATGTACGGG
7861 TTCCGGTTCC CAATGTACGG CTTTGGGTTC CCAATGTACG TGCTATCCAC AGGAAAGAGA
7921 CCTTTTCGAC CTTTTTCCCC TGCTAGGGCA ATTTGCCCTA GCATCTGCTC CGTACATTAG
7981 GAACCGGCGG ATGCTTCGCC CTCGATCAGG TTGCGGTAGC GCATGACTAG GATCGGGCCA
8041 GCCTGCCCCG CCTCCTCCTT CAAATCGTAC TCCGGCAGGT CATTTGACCC GATCAGCTTG
8101 CGCACGGTGA AACAGAACTT CTTGAACTCT CCGGCGCTGC CACTGCGTTC GTAGATCGTC
8161 TTGAACAACC ATCTGGCTTC TGCCTTGCCT GCGGCGCGGC GTGCCAGGCG GTAGAGAAAA
8221 CGGCCGATGC CGGGATCGAT CAAAAAGTAA TCGGGGTGAA CCGTCAGCAC GTCCGGGTTC
8281 TTGCCTTCTG TGATCTCGCG GTACATCCAA TCAGCTAGCT CGATCTCGAT GTACTCCGGC
8341 CGCCCGGTTT CGCTCTTTAC GATCTTGTAG CGGCTAATCA AGGCTTCACC CTCGGATACC
8401 GTCACCAGGC GGCCGTTCTT GGCCTTCTTC GTACGCTGCA TGGCAACGTG CGTGGTGTTT
8461 AACCGAATGC AGGTTTCTAC CAGGTCGTCT TTCTGCTTTC CGCCATCGGC TCGCCGGCAG
8521 AACTTGAGTA CGTCCGCAAC GTGTGGACGG AACACGCGGC CGGGCTTGTC TCCCTTCCCT
8581 TCCCGGTATC GGTTCATGGA TTCGGTTAGA TGGGAAACCG CCATCAGTAC CAGGTCGTAA
8641 TCCCACACAC TGGCCATGCC GGCCGGCCCT GCGGAAACCT CTACGTGCCC GTCTGGAAGC
8701 TCGTAGCGGA TCACCTCGCC AGCTCGTCGG TCACGCTTCG ACAGACGGAA AACGGCCACG
8761 TCCATGATGC TGCGACTATC GCGGGTGCCC ACGTCATAGA GCATCGGAAC GAAAAAATCT
8821 GGTTGCTCGT CGCCCTTGGG CGGCTTCCTA ATCGACGGCG CACCGGCTGC CGGCGGTTGC
8881 CGGGATTCTT TGCGGATTCG ATCAGCGGCC GCTTGCCACG ATTCACCGGG GCGTGCTTCT
8941 GCCTCGATGC GTTGCCGCTG GGCGGCCTGC GCGGCCTTCA ACTTCTCCAC CAGGTCATCA
9001 CCCAGCGCCG CGCCGATTTG TACCGGGCCG GATGGTTTGC GACCGTCACG CCGATTCCTC
9061 GGGCTTGGGG GTTCCAGTGC CATTGCAGGG CCGGCAGACA ACCCAGCCGC TTACGCCTGG
9121 CCAACCGCCC GTTCCTCCAC ACATGGGGCA TTCCACGGCG TCGGTGCCTG GTTGTTCTTG
9181 ATTTTCCATG CCGCCTCCTT TAGCCGCTAA AATTCATCTA CTCATTTATT CATTTGCTCA
9241 TTTACTCTGG TAGCTGCGCG ATGTATTCAG ATAGCAGCTC GGTAATGGTC TTGCCTTGGC
9301 GTACCGCGTA CATCTTCAGC TTGGTGTGAT CCTCCGCCGG CAACTGAAAG TTGACCCGCT
9361 TCATGGCTGG CGTGTCTGCC AGGCTGGCCA ACGTTGCAGC CTTGCTGCTG CGTGCGCTCG
9421 GACGGCCGGC ACTTAGCGTG TTTGTGCTTT TGCTCATTTT CTCTTTACCT CATTAACTCA
9481 AATGAGTTTT GATTTAATTT CAGCGGCCAG CGCCTGGACC TCGCGGGCAG CGTCGCCCTC
9541 GGGTTCTGAT TCAAGAACGG TTGTGCCGGC GGCGGCAGTG CCTGGGTAGC TCACGCGCTG
9601 CGTGATACGG GACTCAAGAA TGGGCAGCTC GTACCCGGCC AGCGCCTCGG CAACCTCACC
9661 GCCGATGCGC GTGCCTTTGA TCGCCCGCGA CACGACAAAG GCCGCTTGTA GCCTTCCATC
9721 CGTGACCTCA ATGCGCTGCT TAACCAGCTC CACCAGGTCG GCGGTGGCCC ATATGTCGTA
9781 AGGGCTTGGC TGCACCGGAA TCAGCACGAA GTCGGCTGCC TTGATCGCGG ACACAGCCAA
9841 GTCCGCCGCC TGGGGCGCTC CGTCGATCAC TACGAAGTCG CGCCGGCCGA TGGCCTTCAC
9901 GTCGCGGTCA ATCGTCGGGC GGTCGATGCC GACAACGGTT AGCGGTTGAT CTTCCCGCAC
9961 GGCCGCCCAA TCGCGGGCAC TGCCCTGGGG ATCGGAATCG ACTAACAGAA CATCGGCCCC
10021 GGCGAGTTGC AGGGCGCGGG CTAGATGGGT TGCGATGGTC GTCTTGCCTG ACCCGCCTTT
10081 CTGGTTAAGT ACAGCGATAA CCTTCATGCG TTCCCCTTGC GTATTTGTTT ATTTACTCAT
10141 CGCATCATAT ACGCAGCGAC CGCATGACGC AAGCTGTTTT ACTCAAATAC ACATCACCTT
10201 TTTAGACGGC GGCGCTCGGT TTCTTCAGCG GCCAAGCTGG CCGGCCAGGC CGCCAGCTTG
10261 GCATCAGACA AACCGGCCAG GATTTCATGC AGCCGCACGG TTGAGACGTG CGCGGGCGGC
10321 TCGAACACGT ACCCGGCCGC GATCATCTCC GCCTCGATCT CTTCGGTAAT GAAAAACGGT
10381 TCGTCCTGGC CGTCCTGGTG CGGTTTCATG CTTGTTCCTC TTGGCGTTCA TTCTCGGCGG
10441 CCGCCAGGGC GTCGGCCTCG GTCAATGCGT CCTCACGGAA GGCACCGCGC CGCCTGGCCT
10501 CGGTGGGCGT CACTTCCTCG CTGCGCTCAA GTGCGCGGTA CAGGGTCGAG CGATGCACGC
10561 CAAGCAGTGC AGCCGCCTCT TTCACGGTGC GGCCTTCCTG GTCGATCAGC TCGCGGGCGT
10621 GCGCGATCTG TGCCGGGGTG AGGGTAGGGC GGGGGCCAAA CTTCACGCCT CGGGCCTTGG
10681 CGGCCTCGCG CCCGCTCCGG GTGCGGTCGA TGATTAGGGA ACGCTCGAAC TCGGCAATGC
10741 CGGCGAACAC GGTCAACACC ATGCGGCCGG CCGGCGTGGT GGTGTCGGCC CACGGCTCTG
10801 CCAGGCTACG CAGGCCCGCG CCGGCCTCCT GGATGCGCTC GGCAATGTCC AGTAGGTCGC
10861 GGGTGCTGCG GGCCAGGCGG TCTAGCCTGG TCACTGTCAC AACGTCGCCA GGGCGTAGGT
10921 GGTCAAGCAT CCTGGCCAGC TCCGGGCGGT CGCGCCTGGT GCCGGTGATC TTCTCGGAAA
10981 ACAGCTTGGT GCAGCCGGCC GCGTGCAGTT CGGCCCGTTG GTTGGTCAAG TCCTGGTCGT
11041 CGGTGCTGAC GCGGGCATAG CCCAGCAGGC CAGCGGCGGC GCTCTTGTTC ATGGCGTAAT
11101 GTCTCCGGTT CTAGTCGCAA GTATTCTACT TTATGCGACT AAAACACGCG ACAAGAAAAC
11161 GCCAGGAAAA GGGCAGGGCG GCAGCCTGTC GCGTAACTTA GGACTTGTGC GACATGTCGT
11221 TTTCAGAAGA CGGCTGCACT GAACGTCAGA AGCCGACTGC ACTATAGCAG CGGAGGGGTT
11281 GGATCAAAGT ACT
```

Supplemental Figure S6. F-SHMT08cΔ+S190A-CDS, insert size, vector, restriction enzymes, and sequenced positive clones used for cloning.

Gene Name F-SHMT08cΔ+H218A-CDS
Cloning Vector pG2RNAi2
Length (bp) 1430
Cloning Sites AscI-AvrII

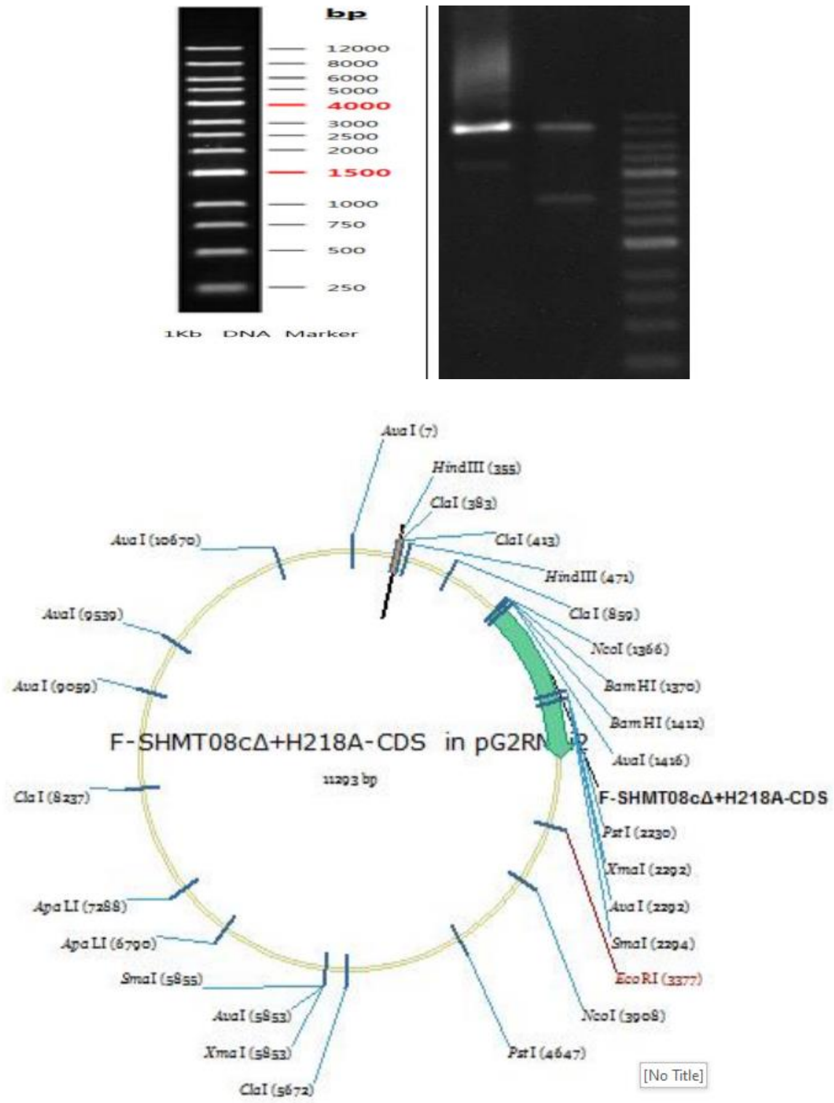

Supplemental Figure S7. F-SHMT08cΔ+H218A-CDS, insert size, vector, restriction enzymes, and sequenced positive clones used for cloning.

Detailed Sequence of the Whole Construct:

```
   1 TTGATCCCGA GGGGAACCCT GTGGTTGGCA TGCACATACA AATGGACGAA CGGATAAACC
  61 TTTTCACGCC CTTTTAAATA TCCGTTATTC TAATAAACGC TCTTTTCTCT TAGGTTTACC
 121 CGCCAATATA TCCTGTCAAA CACTGATAGT TTAAACTGAA GGCGGGAAAC GACAATCTGA
 181 TCCAAGCTCA AGCTCATTCG CCATTCAGGC TGCGCAACTG TTGGGAAGGG CGATCGGTGC
 241 GGGCCTCTTC GCTATTACGC CAGCTGGCGA AAGGGGGATG TGCTGCAAGG CGATTAAGTT
 301 GGGTAACGCC AGGGTTTTCC CAGTCACGAC GTTGTAAAAC GACGGCCAGT GCCAAGCTTA
 361 TTACCCTGTT ATCCCTAGAT ATCGATTTTG GCTACCTTAA GGTAGCCAAA ATCGATCACT
 421 AGTGCGGCCG CGGGCCCAAT ATAACAACGA CGTCGTAACA GATAAAGCGA AGCTTGAAGG
 481 TGCATGTGAC TCCGTCAAGA TTACGAAACC GCCAACTACC ACGCAAATTG CAATTCTCAA
 541 TTTCCTAGAA GGACTCTCCG AAAATGCATC CAATACCAAA TATTACCCGT GTCATAGGCA
 601 CCAAGTGACA CCATACATGA ACACGCGTCA CAATATGACT GGAGAAGGGT TCCACACCTT
 661 ATGCTATAAA ACGCCCCACA CCCCTCCTCC TTCCTTCGCA GTTCAATTCC AATATATTCC
 721 ATTCTCTCTG TGTATTTCCC TACCTCTCCC TTCAAGGTTA GTCGATTTCT TCTGTTTTTC
 781 TTCTTCGTTC TTTCCATGAA TTGTGTATGT TCTTTGATCA ATACGATGTT GATTTGATTG
 841 TGTTTTGTTT GGTTTCATCG ATCTTCAATT TTCATAATCA GATTCAGCTT TTATTATCTT
 901 TACAACAACG TCCTTAATTT GATGATTCTT TAATCGTAGA TTTGCTCTAA TTAGAGCTTT
 961 TTCATGTCAG ATCCCTTTAC AACAAGCCTT AATTGTTGAT TCATTAATCG TAGATTAGGG
1021 CTTTTTTCAT TGATTACTTC AGATCCGTTA AACGTAACCA TAGATCAGGG CTTTTTCATG
1081 AATTACTTCA GATCCGTTAA ACAACAGCCT TATTTTTTAT ACTTCTGTGG TTTTTCAAGA
1141 AATTGTTCAG ATCCGTTGAC AAAAAGCCTT ATTCGTTGAT TCTATATCGT TTTTCGAGAG
1201 ATATTGCTCA GATCTGTTAG CAACTGCCTT GTTTGTTGAT TCTATTGCCG TGGATTAGGG
1261 TTTTTTTTCA CGAGATTGCT TCAGATCCGT ACTTAAGATT ACGTAATGGA TTTTGATTCT
1321 GATTTATCTG TGATTGTTGA CTCGACAGCG GCCGCACCGG CGCGCCATGG ATCCAGTAAG
1381 CGTGTGGGGT AACACGCCCT TGGCGACGGT GGATCCCGAG ATCCATGACC TCATCGAGAA
1441 GGAGAAGCGC CGTCAATGCC GCGGAATCGA GCTCATCGCC TCCGAGAACT TCACCTCCTT
```

Supplemental Figure S7. F-SHMT08cΔ+H218A-CDS, insert size, vector, restriction enzymes, and sequenced positive clones used for cloning.

```
1501 CGCCGTCATC GAGGCCCTCG GCAGCGCTCT CACGAACAAA TACTCCGAGG GCATGCCGGG
1561 CAACCGCTAC TACGGCGGCA ATGAATACAT CGACCAGATC GAAAACCTCT GCCGCTCACG
1621 CGCCCTCCAA GCCTTCCACC TCGACGCCCA ATCCTGGGGC GTCAACGTCC AGCCCTACTC
1681 CGGCTCCCCG GCCAACTTCG CCGCCTACAC CGCCGTCCTC AACCCCCACG ACCGCATCAT
1741 GGGGCTAGAT CTCCGCTCCG GCGGCCACCT CACCCACGGC TACTACACCT CCGGCGGAAA
1801 GAAGATCTCC GCCACCTCCA TTTACTTCGA GAGTCTCCCT TACAAGGTAA ACTCCACCAC
1861 CGGCTACATC GACTACGACC GCTTGGAAGA AAAAGCCCTA GACTTCAGGC CAAAACTCAT
1921 AATCTGCGGT GGCAGCGCGT ACCCTCGCGA TTGGGACTAC AAACGTTTCA GGGAAGTCGC
1981 TGATAAGTGC GGAGCATTGC TTCTCTGCGA CATGGCGGCT ACTAGCGGCC TTGTGGCCGC
2041 GCAGGAAGTG AACAGCCCCT TCGAGTATTG CGACATTGTG ACCACCACGA CTCACAAGAG
2101 CTTGCGGGGC CCACGTGCGG GGATGATCTT TTACCGGAAG GGCCCCAAGC CGCCGAAGAA
2161 GGGGCAGCCG GAGAACGCGG TTTATGATTT CGAGGACAAG ATTAACTTCG CGGTGTTCCC
2221 TTCGCTGCAG GGTGGGCCCC ACAACCACCA GATCGGTGCT CTCGCCGTGG CGCTGAAGCA
2281 GGCCGCGTCG CCCGGGTTTA AGGCCTACGC GAAGCAGGTT AAGGCGAACG CCGTTGCGCT
2341 TGGAAAATAC TTGATGGGGA AAGGGTACAG CCTTGTCACT GGCGGAACGG AGAACCATCT
2401 TGTTTTGTGG GATCTGAGAC CTCTTGGATT GACTGGGTAT AAGGTGGAGA AACTCTGTGA
2461 TCTCTGTAAC ATTACTGTTA ACAAGAACGC TGTTTTTGGT GATAGCAGTG CCTTGGCCCC
2521 TGGTGGAGTG CGAATTGGTG CCCCTGCCAT GACTTCTAGG GGTTTGGTTG AAAAAGACTT
2581 TGAGCAGATT GGTGAGTTCC TTCACCGTGC TGTGACTCTC ACACTGGAGA TCCAGAAGGA
2641 GCATGGCAAA CTTCTCAAGG ATTTCAACAA GGGTCTCGTC AACAACAAGG CTATTGAAGA
2701 TCTCAAAGCT GATGTTGAGA AGTTCTCTGC CTTGTTTGAC ATGCCTGGCT TCCTGGTATC
2761 TGAAATGAAG TACAAGGATT AGCCTAGGTT CGAGTATTAT GGCATTGGGA AAACTGTTTT
2821 TCTTGTACCA TTTGTTGTGC TTGTAATTTA CTGTGTTTTT TATTCGGTTT TCGCTATCGA
2881 ACTGTGAAAT GGAAATGGAT GGAGAAGAGT TAATGAATGA TATGGTCCTT TTGTTCATTC
2941 TCAAATTAAT ATTATTTGTT TTTTCTCTTA TTTGTTGTGT GTTGAATTTG AAATTATAAG
3001 AGATATGCAA ACATTTTGTT TTGAGTAAAA ATGTGTCAAA TCGTGGCCTC TAATGACCGA
3061 AGTTAATATG AGGAGTAAAA CACTTGTAGT TGTACCATTA TGCTTATTCA CTAGGCAACA
3121 AATATATTTT CAGACCTAGA AAAGCTGCAA ATGTTACTGA ATACAAGTAT GTCCTCTTGT
3181 GTTTTAGACA TTTATGAACT TTCCTTTATG TAATTTTCCA GAATCCTTGT CAGATTCTAA
3241 TCATTGCTTT ATAATTATAG TTATACTCAT GGATTTGTAG TTGAGTATGA AAATATTTTT
3301 TAATGCATTT TATGACTTGC CAATTGATTG ACAACATGCA TCAATCCGCG GTTATGACTC
3361 TCTTAAGAGA GTCATGAATT CGAGCTTCCA GAAGGTAATT ATCCAAGATG TAGCATCAAG
3421 AATCCAATGT TTACGGGAAA AACTATGGAA GTATTATGTG AGCTCAGCAA GAAGCAGATC
3481 AATATGCGGC ACATATGCAA CCTATGTTCA AAAATGAAGA ATGTACAGAT ACAAGATCCT
3541 ATACTGCCAG AATACGAAGA AGAATACGTA GAAATTGAAA AAGAAGAACC AGGCGAAGAA
3601 AAGAATCTTG AAGACGTAAG CACTGACGAC AACAATGAAA AGAAGAAGAT AAGGTCGGTG
3661 ATTGTGAAAG AGACATAGAG GACACATGTA AGGTGGAAAA TGTAAGGGCG GAAAGTAACC
3721 TTATCACAAA GGAATCTTAT CCCCCACTAC TTATCCTTTT ATATTTTTCC GTGTCATTTT
3781 TGCCCTTGAG TTTTCCTATA TAAGGAACCA AGTTCGGCAT TTGTGAAAAC AAGAAAAAAT
3841 TTGGTGTAAG CTATTTTCTT TGAAGTACTG AGGATACAAC TTCAGAGAAA TTTGTAAGTT
3901 TGTGATCCAT GGTGAGCAAG GGCGAGGAGC TGTTCACCGG GGTGGTGCCC ATCCTGGTCG
3961 AGCTGGACGG CGACGTAAAC GGCCACAAGT TCAGCGTGTC CGGCGAGGGC GAGGGCGATG
4021 CCACCTACGG CAAGCTGACC CTGAAGTTCA TCTGCACCAC CGGCAAGCTG CCCGTGCCCT
4081 GGCCCACCCT CGTGACCACC TTCACCTACG GCGTGCAGTG CTTCAGCCGC TACCCCGACC
4141 ACATGAAGCA GCACGACTTC TTCAAGTCCG CCATGCCCGA AGGCTACGTC CAGGAGCGCA
4201 CCATCTTCTT CAAGGACGAC GGCAACTACA AGACCCGCGC CGAGGTGAAG TTCGAGGGCG
4261 ACACCCTGGT GAACCGCATC GAGCTGAAGG GCATCGACTT CAAGGAGGAC GGCAACATCC
4321 TGGGGCACAA GCTGGAGTAC AACTACAACA GCCACAACGT CTATATCATG GCCGACAAGC
4381 AGAAGAACGG CATCAAGGTG AACTTCAAGA TCCGCCACAA CATCGAGGAC GGCAGCGTGC
4441 AGCTCGCCGA CCACTACCAG CAGAACACCC CCATCGGCGA CGGCCCCGTG CTGCTGCCCG
4501 ACAACCACTA CCTGAGCACC CAGTCCGCCC TGAGCAAAGA CCCCAACGAG AAGCGCGATC
4561 ACATGGTCCT GCTGGAGTTC GTGACCGCCG CCGGGATCAC TCACGGCATG GACGAGCTGT
4621 ACAAGTAAAG CGGCCGCCCG GCTGCAGATC GTTCAAACAT TTGGCAATAA AGTTTCTTAA
4681 GATTGAATCC TGTTGCCGGT CTTGCGATGA TTATCATATA ATTTCTGTTG AATTACGTTA
```

Supplemental Figure S7. F-SHMT08cΔ+H218A-CDS, insert size, vector, restriction enzymes, and sequenced positive clones used for cloning.

```
4801 GAGTCCCGCA ATTATACATT TAATACGCGA TAGAAAACAA AATATAGCGC GCAAACTAGG
4861 ATAAATTATC GCGCGCGGTG TCATCTATGT TACTAGATCC GATGATAAGC TGTCAAACAT
4921 GAGAATTAAT TCGTAATCAT GTCATAGCTG TTTCCTGTGT GAAATTGTTA TCCGCTCACA
4981 ATTCCACACA ACATACGAGC CGGAAGCATA AAGTGTAAAG CCTGGGGTGC CTAATGAGTG
5041 AGCTAACTCA CATTAATTGC GTTGCGCTCA CTGCCCGCTT TCCAGTCGGG AAACCTGTCG
5101 TGCCAGCTGC ATTAATGAAT CGGCCAACGC GCGGGGAGAG GCGGTTTGCG TATTGGCTAG
5161 AGCAATTCGG CGTTAATTCA GTACATTAAA AACGTCCGCA ATGTGTTATT AAGTTGTCTA
5221 AGCGTCAATT TGTTTACACC ACAATATATC CTGCCACCAG CCAGCCAACA GCTCCCCGAC
5281 CGGCAGCTCG GCACAAAATC ACCACTCGAT ACAGGCAGCC CATCAGTCCG GGACGGCGTC
5341 AGCGGGAGAG CCGTTGTAAG GCGGCAGACT TTGCTCATGT TACCGATGCT ATTCGGAAGA
5401 ACGGCAACTA AGCTGCCGGG TTTGAAACAC GGATGATCTC GCGGAGGGTA GCATGTTGAT
5461 TGTAACGATG ACAGAGCGTT GCTGCCTGTG ATCCAGATCA TGAACAATAA AACTGTCTGC
5521 TTACATAAAC AGTAATACAA GGGGTGTTAT GAGCCATATT CAACGGGAAA CGTCTTGCTC
5581 TAGGCCGCGA TTAAATTCCA ACATGGATGC TGATTTATAT GGGTATAAAT GGGCTCGCGA
5641 TAATGTCGGG CAATCAGGTG CGACAATCTA TCGATTGTAT GGGAAGCCCG ATGCGCCAGA
5701 GTTGTTTCTG AAACATGGCA AAGGTAGCGT TGCCAATGAT GTTACAGATG AGATGGTCAG
5761 ACTAAACTGG CTGACGGAAT TTATGCCTCT TCCGACCATC AAGCATTTTA TCCGTACTCC
5821 TGATGATGCA TGGTTACTCA CCACTGCGAT CCCCGGGAAA ACAGCATTCC AGGTATTAGA
5881 AGAATATCCT GATTCAGGTG AAAATATTGT TGATGCGCTG GCAGTGTTCC TGCGCCGGTT
5941 GCATTCGATT CCTGTTTGTA ATTGTCCTTT TAACAGCGAT CGCGTATTTC GTCTCGCTCA
6001 GGCGCAATCA CGAATGAATA ACGGTTTGGT TGATGCGAGT GATTTTGATG ACGAGCGTAA
6061 TGGCTGGCCT GTTGAACAAG TCTGGAAAGA AATGCATAAA CTTTTGCCAT TCTCACCGGA
6121 TTCAGTCGTC ACTCATGGTG ATTTCTCACT TGATAACCTT ATTTTTGACG AGGGGAAATT
6181 AATAGGTTGT ATTGATGTTG GACGAGTCGG AATCGCAGAC CGATACCAGG ATCTTGCCAT
6241 CCTATGGAAC TGCCTCGGTG AGTTTTCTCC TTCATTACAG AAACGGCTTT TTCAAAAATA
6301 TGGTATTGAT AATCCTGATA TGAATAAATT GCAGTTTCAT TTGATGCTCG ATGAGTTTTT
6361 CTAAGAATTA ATTCATGATC CTGCATGACC AAAATCCCTT AACGTGAGTT TTCGTTCCAC
6421 TGAGCGTCAG ACCCCGTAGA AAAGATCAAA GGATCTTCTT GAGATCCTTT TTTTCTGCGC
6481 GTAATCTGCT GCTTGCAAAC AAAAAAACCA CCGCTACCAG CGGTGGTTTG TTTGCCGGAT
6541 CAAGAGCTAC CAACTCTTTT TCCGAAGGTA ACTGGCTTCA GCAGAGCGCA GATACCAAAT
6601 ACTGTCCTTC TAGTGTAGCC GTAGTTAGGC CACCACTTCA AGAACTCTGT AGCACCGCCT
6661 ACATACCTCG CTCTGCTAAT CCTGTTACCA GTGGCTGCTG CCAGTGGCGA TAAGTCGTGT
6721 CTTACCGGGT TGGACTCAAG ACGATAGTTA CCGGATAAGG CGCAGCGGTC GGGCTGAACG
6781 GGGGGTTCGT GCACACAGCC CAGCTTGGAG CGAACGACCT ACACCGAACT GAGATACCTA
6841 CAGCGTGAGC TATGAGAAAG CGCCACGCTT CCCGAAGGGA GAAAGGCGGA CAGGTATCCG
6901 GTAAGCGGCA GGGTCGGAAC AGGAGAGCGC ACGAGGGAGC TTCCAGGGGG AAACGCCTGG
6961 TATCTTTATA GTCCTGTCGG GTTTCGCCAC CTCTGACTTG AGCGTCGATT TTTGTGATGC
7021 TCGTCAGGGG GGCGGAGCCT ATGGAAAAAC GCCAGCAACG CGGCCTTTTT ACGGTTCCTG
7081 GCCTTTTGCT GGCCTTTTGC TCACATGTTC TTTCCTGCGT TATCCCCTGA TTCTGTGGAT
7141 AACCGTATTA CCGCCTTTGA GTGAGCTGAT ACCGCTCGCC GCAGCCGAAC GACCGAGCGC
7201 AGCGAGTCAG TGAGCGAGGA AGCGGAAGAG CGCCTGATGC GGTATTTTCT CCTTACGCAT
7261 CTGTGCGGTA TTTCACACCG CATATGGTGC ACTCTCAGTA CAATCTGCTC TGATGCCGCA
7321 TAGTTAAGCC AGTATACACT CCGCTATCGC TACGTGACTG GGTCATGGCT GCGCCCCGAC
7381 ACCCGCCAAC ACCCGCTGAC GCGCCCTGAC GGGCTTGTCT GCTCCCGGCA TCCGCTTACA
7441 GACAAGCTGT GACCGTCTCC GGGAGCTGCA TGTGTCAGAG GTTTTCACCG TCATCACCGA
7501 AACGCGCGAG GCAGGGTGCC TTGATGTGGG CGCCGGCGGT CGAGTGGCGA CGGCGCGGCT
7561 TGTCCGCGCC CTGGTAGATT GCCTGGCCGT AGGCCAGCCA TTTTTGAGCG GCCAGCGGCC
7621 GCGATAGGCC GACGCGAAGC GGCGGGGCGT AGGGAGCGCA GCGACCGAAG GGTAGGCGCT
7681 TTTTGCAGCT CTTCGGCTGT GCGCTGGCCA GACAGTTATG CACAGGCCAG GCGGGTTTTA
7741 AGAGTTTTAA TAAGTTTTAA AGAGTTTTAG GCGGAAAAAT CGCCTTTTTT CTCTTTTATA
7801 TCAGTCACTT ACATGTGTGA CCGGTTCCCA ATGTACGGCT TTGGGTTCCC AATGTACGGG
7861 TTCCGGTTCC CAATGTACGG CTTTGGGTTC CCAATGTACG TGCTATCCAC AGGAAAGAGA
7921 CCTTTTCGAC CTTTTTCCCC TGCTAGGGCA ATTTGCCCTA GCATCTGCTC CGTACATTAG
7981 GAACCGGCGG ATGCTTCGCC CTCGATCAGG TTGCGGTAGC GCATGACTAG GATCGGGCCA
8041 GCCTGCCCCG CCTCCTCCTT CAAATCGTAC TCCGGCAGGT CATTTGACCC GATCAGCTTG
8101 CGCACGGTGA AACAGAACTT CTTGAACTCT CCGGCGCTGC CACTGCGTTC GTAGATCGTC
8161 TTGAACAACC ATCTGGCTTC TGCCTTGCCT GCGGCGCGGC GTGCCAGGCG GTAGAGAAAA
8221 CGGCCGATGC CGGGATCGAT CAAAAAGTAA TCGGGGTGAA CCGTCAGCAC GTCCGGGTTC
8281 TTGCCTTCTG TGATCTCGCG GTACATCCAA TCAGCTAGCT CGATCTCGAT GTACTCCGGC
8341 CGCCCGGTTT CGCTCTTTAC GATCTTGTAG CGGCTAATCA AGGCTTCACC CTCGGATACC
8401 GTCACCAGGC GGCCGTTCTT GGCCTTCTTC GTACGCTGCA TGGCAACGTG CGTGGTGTTT
8461 AACCGAATGC AGGTTTCTAC CAGGTCGTCT TTCTGCTTTC CGCCATCGGC TCGCCGGCAG
8521 AACTTGAGTA CGTCCGCAAC GTGTGGACGG AACACGCGGC CGGGCTTGTC TCCCTTCCCT
8581 TCCCGGTATC GGTTCATGGA TTCGGTTAGA TGGGAAACCG CCATCAGTAC CAGGTCGTAA
8641 TCCCACACAC TGGCCATGCC GGCCGGCCCT GCGGAAACCT CTACGTGCCC GTCTGGAAGC
8701 TCGTAGCGGA TCACCTCGCC AGCTCGTCGG TCACGCTTCG ACAGACGGAA AACGGCCACG
8761 TCCATGATGC TGCGACTATC GCGGGTGCCC ACGTCATAGA GCATCGGAAC GAAAAAATCT
8821 GGTTGCTCGT CGCCCTTGGG CGGCTTCCTA ATCGACGGCG CACCGGCTGC CGGCGGTTGC
8881 CGGGATTCTT TGCGGATTCG ATCAGCGGCC GCTTGCCACG ATTCACCGGG GCGTGCTTCT
8941 GCCTCGATGC GTTGCCGCTG GGCGGCCTGC GCGGCCTTCA ACTTCTCCAC CAGGTCATCA
9001 CCCAGCGCCG CGCCGATTTG TACCGGGCCG GATGGTTTGC GACCGTCACG CCGATTCCTC
9061 GGGCTTGGGG GTTCCAGTGC CATTGCAGGG CCGGCAGACA ACCCAGCCGC TTACGCCTGG
9121 CCAACCGCCC GTTCCTCCAC ACATGGGGCA TTCCACGGCG TCGGTGCCTG GTTGTTCTTG
9181 ATTTTCCATG CCGCCTCCTT TAGCCGCTAA AATTCATCTA CTCATTTATT CATTTGCTCA
9241 TTTACTCTGG TAGCTGCGCG ATGTATTCAG ATAGCAGCTC GGTAATGGTC TTGCCTTGGC
9301 GTACCGCGTA CATCTTCAGC TTGGTGTGAT CCTCCGCCGG CAACTGAAAG TTGACCCGCT
9361 TCATGGCTGG CGTGTCTGCC AGGCTGGCCA ACGTTGCAGC CTTGCTGCTG CGTGCGCTCG
9421 GACGGCCGGC ACTTAGCGTG TTTGTGCTTT TGCTCATTTT CTCTTTACCT CATTAACTCA
9481 AATGAGTTTT GATTTAATTT CAGCGGCCAG CGCCTGGACC TCGCGGGCAG CGTCGCCCTC
9541 GGGTTCTGAT TCAAGAACGG TTGTGCCGGC GGCGGCAGTG CCTGGGTAGC TCACGCGCTG
9601 CGTGATACGG GACTCAAGAA TGGGCAGCTC GTACCCGGCC AGCGCCTCGG CAACCTCACC
9661 GCCGATGCGC GTGCCTTTGA TCGCCCGCGA CACGACAAAG GCCGCTTGTA GCCTTCCATC
9721 CGTGACCTCA ATGCGCTGCT TAACCAGCTC CACCAGGTCG GCGGTGGCCC ATATGTCGTA
9781 AGGGCTTGGC TGCACCGGAA TCAGCACGAA GTCGGCTGCC TTGATCGCGG ACACAGCCAA
9841 GTCCGCCGCC TGGGGCGCTC CGTCGATCAC TACGAAGTCG CGCCGGCCGA TGGCCTTCAC
9901 GTCGCGGTCA ATCGTCGGGC GGTCGATGCC GACAACGGTT AGCGGTTGAT CTTCCCGCAC
9961 GGCCGCCCAA TCGCGGGCAC TGCCCTGGGG ATCGGAATCG ACTAACAGAA CATCGGCCCC
10021 GGCGAGTTGC AGGGCGCGGG CTAGATGGGT TGCGATGGTC GTCTTGCCTG ACCCGCCTTT
10081 CTGGTTAAGT ACAGCGATAA CCTTCATGCG TTCCCCTTGC GTATTTGTTT ATTTACTCAT
10141 CGCATCATAT ACGCAGCGAC CGCATGACGC AAGCTGTTTT ACTCAAATAC ACATCACCTT
10201 TTTAGACGGC GGCGCTCGGT TTCTTCAGCG GCCAAGCTGG CCGGCCAGGC CGCCAGCTTG
10261 GCATCAGACA AACCGGCCAG GATTTCATGC AGCCGCACGG TTGAGACGTG CGCGGGCGGC
10321 TCGAACACGT ACCCGGCCGC GATCATCTCC GCCTCGATCT CTTCGGTAAT GAAAAACGGT
10381 TCGTCCTGGC CGTCCTGGTG CGGTTTCATG CTTGTTCCTC TTGGCGTTCA TTCTCGGCGG
10441 CCGCCAGGGC GTCGGCCTCG GTCAATGCGT CCTCACGGAA GGCACCGCGC CGCCTGGCCT
10501 CGGTGGGCGT CACTTCCTCG CTGCGCTCAA GTGCGCGGTA CAGGGTCGAG CGATGCACGC
10561 CAAGCAGTGC AGCCGCCTCT TTCACGGTGC GGCCTTCCTG GTCGATCAGC TCGCGGGCGT
10621 GCGCGATCTG TGCCGGGGTG AGGGTAGGGC GGGGGCCAAA CTTCACGCCT CGGGCCTTGG
10681 CGGCCTCGCG CCCGCTCCGG GTGCGGTCGA TGATTAGGGA ACGCTCGAAC TCGGCAATGC
10741 CGGCGAACAC GGTCAACACC ATGCGGCCGG CCGGCGTGGT GGTGTCGGCC CACGGCTCTG
10801 CCAGGCTACG CAGGCCCGCG CCGGCCTCCT GGATGCGCTC GGCAATGTCC AGTAGGTCGC
10861 GGGTGCTGCG GGCCAGGCGG TCTAGCCTGG TCACTGTCAC AACGTCGCCA GGGCGTAGGT
10921 GGTCAAGCAT CCTGGCCAGC TCCGGGCGGT CGCGCCTGGT GCCGGTGATC TTCTCGGAAA
10981 ACAGCTTGGT GCAGCCGGCC GCGTGCAGTT CGGCCCGTTG GTTGGTCAAG TCCTGGTCGT
11041 CGGTGCTGAC GCGGGCATAG CCCAGCAGGC CAGCGGCGGC GCTCTTGTTC ATGGCGTAAT
11101 GTCTCCGGTT CTAGTCGCAA GTATTCTACT TTATGCGACT AAAACACGCG ACAAGAAAAC
11161 GCCAGGAAAA GGGCAGGGCG GCAGCCTGTC GCGTAACTTA GGACTTGTGC GACATGTCGT
11221 TTTCAGAAGA CGGCTGCACT GAACGTCAGA AGCCGACTGC ACTATAGCAG CGGAGGGGTT
11281 GGATCAAAGT ACT
```

Supplemental Figure S7. F-SHMT08cΔ+H218A-CDS, insert size, vector, restriction enzymes, and sequenced positive clones used for cloning.

Gene Name F-SHMT08cΔ+G106A/S107A-CDS
Cloning Vector pG2RNAi2
Length (bp) 1430
Cloning Sites AscI-AvrII

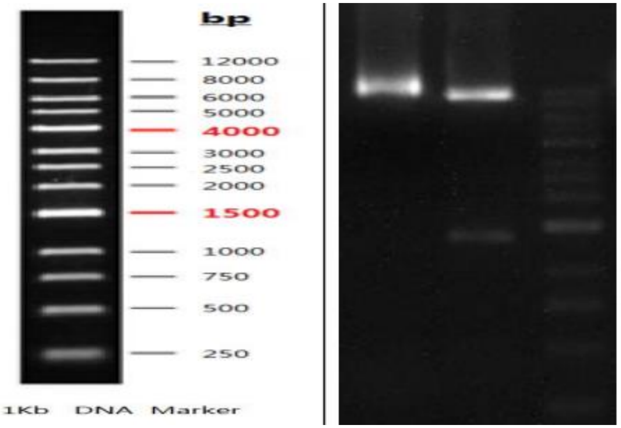

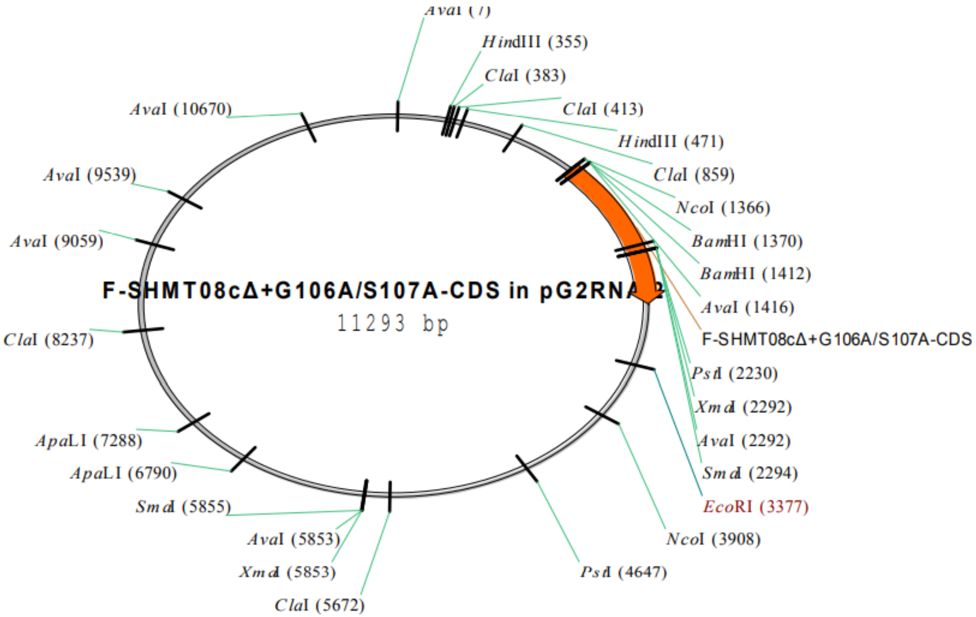

Supplemental Figure S8. F-SHMT08cΔ+G106A/S107A-CDS, insert size, vector, restriction enzymes, and sequenced positive clones used for cloning.

Detailed Sequence of the Whole Construct:

```
   1 TTGATCCCGA GGGGAACCCT GTGGTTGGCA TGCACATACA AATGGACGAA CGGATAAACC
  61 TTTTCACGCC CTTTTAAATA TCCGTTATTC TAATAAACGC TCTTTTCTCT TAGGTTTACC
 121 CGCCAATATA TCCTGTCAAA CACTGATAGT TTAAACTGAA GGCGGGAAAC GACAATCTGA
 181 TCCAAGCTCA AGCTCATTCG CCATTCAGGC TGCGCAACTG TTGGGAAGGG CGATCGGTGC
 241 GGGCCTCTTC GCTATTACGC CAGCTGGCGA AAGGGGGATG TGCTGCAAGG CGATTAAGTT
 301 CCCTAACCCC ACCCTTTTCC CACTCACCAC CTTCTAAAAC CACCCCCACT CCCAACCTTA
 361 TTACCCTGTT ATCCCTAGAT ATCGATTTTG GCTACCTTAA GGTAGCCAAA ATCGATCACT
 421 AGTGCGGCCG CGGGCCCAAT ATAACAACGA CGTCGTAACA GATAAAGCGA AGCTTGAAGG
 481 TGCATGTGAC TCCGTCAAGA TTACGAAACC GCCAACTACC ACGCAAATTG CAATTCTCAA
 541 TTTCCTAGAA GGACTCTCCG AAAATGCATC CAATACCAAA TATTACCCGT GTCATAGGCA
 601 CCAAGTGACA CCATACATGA ACACGCGTCA CAATATGACT GGAGAAGGGT TCCACACCTT
 661 ATGCTATAAA ACGCCCCACA CCCCTCCTCC TTCCTTCGCA GTTCAATTCC AATATATTCC
 721 ATTCTCTCTG TGTATTTCCC TACCTCTCCC TTCAAGGTTA GTCGATTTCT TCTGTTTTTC
 781 TTCTTCGTTC TTTCCATGAA TTGTGTATGT TCTTTGATCA ATACGATGTT GATTTGATTG
 841 TGTTTTGTTT GGTTTCATCG ATCTTCAATT TTCATAATCA GATTCAGCTT TTATTATCTT
 901 TACAACAACG TCCTTAATTT GATGATTCTT TAATCGTAGA TTTGCTCTAA TTAGAGCTTT
 961 TTCATGTCAG ATCCCTTTAC AACAAGCCTT AATTGTTGAT TCATTAATCG TAGATTAGGG
1021 CTTTTTTCAT TGATTACTTC AGATCCGTTA AACGTAACCA TAGATCAGGG CTTTTTCATG
1081 AATTACTTCA GATCCGTTAA ACAACAGCCT TATTTTTTAT ACTTCTGTGG TTTTTCAAGA
1141 AATTGTTCAG ATCCGTTGAC AAAAAGCCTT ATTCGTTGAT TCTATATCGT TTTTCGAGAG
1201 ATATTGCTCA GATCTGTTAG CAACTGCCTT GTTTGTTGAT TCTATTGCCG TGGATTAGGG
1261 TTTTTTTTCA CGAGATTGCT TCAGATCCGT ACTTAAGATT ACGTAATGGA TTTTGATTCT
1321 GATTTATCTG TGATTGTTGA CTCGACAGCG GCCGCACCGG CGCGCCATGG ATCCAGTAAG
1381 CGTGTGGGGT AACACGCCCT TGGCGACGGT GGATCCCGAG ATCCATGACC TCATCGAGAA
1441 GGAGAAGCGC CGTCAATGCC GCGGAATCGA GCTCATCGCC TCCGAGAACT TCACCTCCTT
1501 CGCCGTCATC GAGGCCCTCG GCAGCGCTCT CACGAACAAA TACTCCGAGG GCATGCCGGG
1561 CAACCGCTAC TACGGCGGCA ATGAATACAT CGACCAGATC GAAAACCTCT GCCGCTCACG
1621 CGCCCTCCAA GCCTTCCACC TCGACGCCCA ATCCTGGGGC GTCAACGTCC AGCCCTACTC
1681 CGCTGCTCCG GCCAACTTCG CCGCCTACAC CGCCGTCCTC AACCCCCACG ACCGCATCAT
1741 GGGGCTAGAT CTCCGCTCCG GCGGCCACCT CACCCACGGC TACTACACCT CCGGCGGAAA
```

Supplemental Figure S8. F-SHMT08cΔ+G106A/S107A-CDS, insert size, vector, restriction enzymes, and sequenced positive clones used for cloning.

```
1801 GAAGATCTCC GCCACCTCCA TTTACTTCGA GAGTCTCCCT TACAAGGTAA ACTCCACCAC
1861 CGGCTACATC GACTACGACC GCTTGGAAGA AAAAGCCCTA GACTTCAGGC CAAAACTCAT
1921 AATCTGCGGT GGCAGCGCGT ACCCTCGCGA TTGGGACTAC AAACGTTTCA GGGAAGTCGC
1981 TGATAAGTGC GGAGCATTGC TTCTCTGCGA CATGGCGCAC ACTAGCGGCC TTGTGGCCGC
2041 GCAGGAAGTG AACAGCCCCT TCGAGTATTG CGACATTGTG ACCACCACGA CTCACAAGAG
2101 CTTGCGGGGC CCACGTGCGG GGATGATCTT TTACCGGAAG GGCCCCAAGC CGCCGAAGAA
2161 GGGGCAGCCG GAGAACGCGG TTTATGATTT CGAGGACAAG ATTAACTTCG CGGTGTTCCC
2221 TTCGCTGCAG GGTGGGCCCC ACAACCACCA GATCGGTGCT CTCGCCGTGG CGCTGAAGCA
2281 GGCCGCGTCG CCCGGGTTTA AGGCCTACGC GAAGCAGGTT AAGGCGAACG CCGTTGCGCT
2341 TGGAAAATAC TTGATGGGGA AAGGGTACAG CCTTGTCACT GGCGGAACGG AGAACCATCT
2401 TGTTTTGTGG GATCTGAGAC CTCTTGGATT GACTGGGTAT AAGGTGGAGA AACTCTGTGA
2461 TCTCTGTAAC ATTACTGTTA ACAAGAACGC TGTTTTTGGT GATAGCAGTG CCTTGGCCCC
2521 TGGTGGAGTG CGAATTGGTG CCCCTGCCAT GACTTCTAGG GGTTTGGTTG AAAAAGACTT
2581 TGAGCAGATT GGTGAGTTCC TTCACCGTGC TGTGACTCTC ACACTGGAGA TCCAGAAGGA
2641 GCATGGCAAA CTTCTCAAGG ATTTCAACAA GGGTCTCGTC AACAACAAGG CTATTGAAGA
2701 TCTCAAAGCT GATGTTGAGA AGTTCTCTGC CTTGTTTGAC ATGCCTGGCT TCCTGGTATC
2761 TGAAATGAAG TACAAGGATT AGCCTAGGTT CGAGTATTAT GGCATTGGGA AAACTGTTTT
2821 TCTTGTACCA TTTGTTGTGC TTGTAATTTA CTGTGTTTTT TATTCGGTTT TCGCTATCGA
2881 ACTGTGAAAT GGAAATGGAT GGAGAAGAGT TAATGAATGA TATGGTCCTT TTGTTCATTC
2941 TCAAATTAAT ATTATTTGTT TTTTCTCTTA TTTGTTGTGT GTTGAATTTG AAATTATAAG
3001 AGATATGCAA ACATTTTGTT TTGAGTAAAA ATGTGTCAAA TCGTGGCCTC TAATGACCGA
3061 AGTTAATATG AGGAGTAAAA CACTTGTAGT TGTACCATTA TGCTTATTCA CTAGGCAACA
3121 AATATATTTT CAGACCTAGA AAAGCTGCAA ATGTTACTGA ATACAAGTAT GTCCTCTTGT
3181 GTTTTAGACA TTTATGAACT TTCCTTTATG TAATTTTCCA GAATCCTTGT CAGATTCTAA
3241 TCATTGCTTT ATAATTATAG TTATACTCAT GGATTTGTAG TTGAGTATGA AAATATTTTT
3301 TAATGCATTT TATGACTTGC CAATTGATTG ACAACATGCA TCAATCCGCG GTTATGACTC
3361 TCTTAAGAGA GTCATGAATT CGAGCTTCCA GAAGGTAATT ATCCAAGATG TAGCATCAAG
3421 AATCCAATGT TTACGGGAAA AACTATGGAA GTATTATGTG AGCTCAGCAA GAAGCAGATC
3481 AATATGCGGC ACATATGCAA CCTATGTTCA AAAATGAAGA ATGTACAGAT ACAAGATCCT
3541 ATACTGCCAG AATACGAAGA AGAATACGTA GAAATTGAAA AAGAAGAACC AGGCGAAGAA
3601 AAGAATCTTG AAGACGTAAG CACTGACGAC AACAATGAAA AGAAGAAGAT AAGGTCGGTG
3661 ATTGTGAAAG AGACATAGAG GACACATGTA AGGTGGAAAA TGTAAGGGCG GAAAGTAACC
3721 TTATCACAAA GGAATCTTAT CCCCCACTAC TTATCCTTTT ATATTTTTCC GTGTCATTTT
3781 TGCCCTTGAG TTTTCCTATA TAAGGAACCA AGTTCGGCAT TTGTGAAAAC AAGAAAAAAT
3841 TTGGTGTAAG CTATTTTCTT TGAAGTACTG AGGATACAAC TTCAGAGAAA TTTGTAAGTT
3901 TGTGATCCAT GGTGAGCAAG GGCGAGGAGC TGTTCACCGG GGTGGTGCCC ATCCTGGTCG
3961 AGCTGGACGG CGACGTAAAC GGCCACAAGT TCAGCGTGTC CGGCGAGGGC GAGGGCGATG
4021 CCACCTACGG CAAGCTGACC CTGAAGTTCA TCTGCACCAC CGGCAAGCTG CCCGTGCCCT
4081 GGCCCACCCT CGTGACCACC TTCACCTACG GCGTGCAGTG CTTCAGCCGC TACCCCGACC
4141 ACATGAAGCA GCACGACTTC TTCAAGTCCG CCATGCCCGA AGGCTACGTC CAGGAGCGCA
4201 CCATCTTCTT CAAGGACGAC GGCAACTACA AGACCCGCGC CGAGGTGAAG TTCGAGGGCG
4261 ACACCCTGGT GAACCGCATC GAGCTGAAGG GCATCGACTT CAAGGAGGAC GGCAACATCC
4321 TGGGGCACAA GCTGGAGTAC AACTACAACA GCCACAACGT CTATATCATG GCCGACAAGC
4381 AGAAGAACGG CATCAAGGTG AACTTCAAGA TCCGCCACAA CATCGAGGAC GGCAGCGTGC
4441 AGCTCGCCGA CCACTACCAG CAGAACACCC CCATCGGCGA CGGCCCCGTG CTGCTGCCCG
4501 ACAACCACTA CCTGAGCACC CAGTCCGCCC TGAGCAAAGA CCCCAACGAG AAGCGCGATC
4561 ACATGGTCCT GCTGGAGTTC GTGACCGCCG CCGGGATCAC TCACGGCATG GACGAGCTGT
4621 ACAAGTAAAG CGGCCGCCCG GCTGCAGATC GTTCAAACAT TTGGCAATAA AGTTTCTTAA
4681 GATTGAATCC TGTTGCCGGT CTTGCGATGA TTATCATATA ATTTCTGTTG AATTACGTTA
4741 AGCATGTAAT AATTAACATG TAATGCATGA CGTTATTTAT GAGATGGGTT TTTATGATTA
4801 GAGTCCCGCA ATTATACATT TAATACGCGA TAGAAAACAA AATATAGCGC GCAAACTAGG
4861 ATAAATTATC GCGCGCGGTG TCATCTATGT TACTAGATCC GATGATAAGC TGTCAAACAT
4921 GAGAATTAAT TCGTAATCAT GTCATAGCTG TTTCCTGTGT GAAATTGTTA TCCGCTCACA
4981 ATTCCACACA ACATACGAGC CGGAAGCATA AAGTGTAAAG CCTGGGGTGC CTAATGAGTG
```

Supplemental Figure S8. F-SHMT08cΔ+G106A/S107A-CDS, insert size, vector, restriction enzymes, and sequenced positive clones used for cloning.

```
5041 AGCTAACTCA CATTAATTGC GTTGCGCTCA CTGCCCGCTT TCCAGTCGGG AAACCTGTCG
5101 TGCCAGCTGC ATTAATGAAT CGGCCAACGC GCGGGGAGAG GCGGTTTGCG TATTGGCTAG
5161 AGCAATTCGG CGTTAATTCA GTACATTAAA AACGTCCGCA ATGTGTTATT AAGTTGTCTA
5221 AGCGTCAATT TGTTTACACC ACAATATATC CTGCCACCAG CCAGCCAACA GCTCCCCGAC
5281 CGGCAGCTCG GCACAAAATC ACCACTCGAT ACAGGCAGCC CATCAGTCCG GGACGGCGTC
5341 AGCGGGAGAG CCGTTGTAAG GCGGCAGACT TTGCTCATGT TACCGATGCT ATTCGGAAGA
5401 ACGGCAACTA AGCTGCCGGG TTTGAAACAC GGATGATCTC GCGGAGGGTA GCATGTTGAT
5461 TGTAACGATG ACAGAGCGTT GCTGCCTGTG ATCCAGATCA TGAACAATAA AACTGTCTGC
5521 TTACATAAAC AGTAATACAA GGGGTGTTAT GAGCCATATT CAACGGGAAA CGTCTTGCTC
5581 TAGGCCGCGA TTAAATTCCA ACATGGATGC TGATTTATAT GGGTATAAAT GGGCTCGCGA
5641 TAATGTCGGG CAATCAGGTG CGACAATCTA TCGATTGTAT GGGAAGCCCG ATGCGCCAGA
5701 GTTGTTTCTG AAACATGGCA AAGGTAGCGT TGCCAATGAT GTTACAGATG AGATGGTCAG
5761 ACTAAACTGG CTGACGGAAT TTATGCCTCT TCCGACCATC AAGCATTTTA TCCGTACTCC
5821 TGATGATGCA TGGTTACTCA CCACTGCGAT CCCCGGGAAA ACAGCATTCC AGGTATTAGA
5881 AGAATATCCT GATTCAGGTG AAAATATTGT TGATGCGCTG GCAGTGTTCC TGCGCCGGTT
5941 GCATTCGATT CCTGTTTGTA ATTGTCCTTT TAACAGCGAT CGCGTATTTC GTCTCGCTCA
6001 GGCGCAATCA CGAATGAATA ACGGTTTGGT TGATGCGAGT GATTTTGATG ACGAGCGTAA
6061 TGGCTGGCCT GTTGAACAAG TCTGGAAAGA AATGCATAAA CTTTTGCCAT TCTCACCGGA
6121 TTCAGTCGTC ACTCATGGTG ATTTCTCACT TGATAACCTT ATTTTTGACG AGGGGAAATT
6181 AATAGGTTGT ATTGATGTTG GACGAGTCGG AATCGCAGAC CGATACCAGG ATCTTGCCAT
6241 CCTATGGAAC TGCCTCGGTG AGTTTTCTCC TTCATTACAG AAACGGCTTT TTCAAAAATA
6301 TGGTATTGAT AATCCTGATA TGAATAAATT GCAGTTTCAT TTGATGCTCG ATGAGTTTTT
6361 CTAAGAATTA ATTCATGATC CTGCATGACC AAAATCCCTT AACGTGAGTT TTCGTTCCAC
6421 TGAGCGTCAG ACCCCGTAGA AAAGATCAAA GGATCTTCTT GAGATCCTTT TTTTCTGCGC
6481 GTAATCTGCT GCTTGCAAAC AAAAAAACCA CCGCTACCAG CGGTGGTTTG TTTGCCGGAT
6541 CAAGAGCTAC CAACTCTTTT TCCGAAGGTA ACTGGCTTCA GCAGAGCGCA GATACCAAAT
6601 ACTGTCCTTC TAGTGTAGCC GTAGTTAGGC CACCACTTCA AGAACTCTGT AGCACCGCCT
6661 ACATACCTCG CTCTGCTAAT CCTGTTACCA GTGGCTGCTG CCAGTGGCGA TAAGTCGTGT
6721 CTTACCGGGT TGGACTCAAG ACGATAGTTA CCGGATAAGG CGCAGCGGTC GGGCTGAACG
6781 GGGGGTTCGT GCACACAGCC CAGCTTGGAG CGAACGACCT ACACCGAACT GAGATACCTA
6841 CAGCGTGAGC TATGAGAAAG CGCCACGCTT CCCGAAGGGA GAAAGGCGGA CAGGTATCCG
6901 GTAAGCGGCA GGGTCGGAAC AGGAGAGCGC ACGAGGGAGC TTCCAGGGGG AAACGCCTGG
6961 TATCTTTATA GTCCTGTCGG GTTTCGCCAC CTCTGACTTG AGCGTCGATT TTTGTGATGC
7021 TCGTCAGGGG GGCGGAGCCT ATGGAAAAAC GCCAGCAACG CGGCCTTTTT ACGGTTCCTG
7081 GCCTTTTGCT GGCCTTTTGC TCACATGTTC TTTCCTGCGT TATCCCCTGA TTCTGTGGAT
7141 AACCGTATTA CCGCCTTTGA GTGAGCTGAT ACCGCTCGCC GCAGCCGAAC GACCGAGCGC
7201 AGCGAGTCAG TGAGCGAGGA AGCGGAAGAG CGCCTGATGC GGTATTTTCT CCTTACGCAT
7261 CTGTGCGGTA TTTCACACCG CATATGGTGC ACTCTCAGTA CAATCTGCTC TGATGCCGCA
7321 TAGTTAAGCC AGTATACACT CCGCTATCGC TACGTGACTG GGTCATGGCT GCGCCCCGAC
7381 ACCCGCCAAC ACCCGCTGAC GCGCCCTGAC GGGCTTGTCT GCTCCCGGCA TCCGCTTACA
7441 GACAAGCTGT GACCGTCTCC GGGAGCTGCA TGTGTCAGAG GTTTTCACCG TCATCACCGA
7501 AACGCGCGAG GCAGGGTGCC TTGATGTGGG CGCCGGCGGT CGAGTGGCGA CGGCGCGGCT
7561 TGTCCGCGCC CTGGTAGATT GCCTGGCCGT AGGCCAGCCA TTTTTGAGCG GCCAGCGGCC
7621 GCGATAGGCC GACGCGAAGC GGCGGGGCGT AGGGAGCGCA GCGACCGAAG GGTAGGCGCT
7681 TTTTGCAGCT CTTCGGCTGT GCGCTGGCCA GACAGTTATG CACAGGCCAG GCGGGTTTTA
7741 AGAGTTTTAA TAAGTTTTAA AGAGTTTTAG GCGGAAAAAT CGCCTTTTTT CTCTTTTATA
7801 TCAGTCACTT ACATGTGTGA CCGGTTCCCA ATGTACGGCT TTGGGTTCCC AATGTACGGG
7861 TTCCGGTTCC CAATGTACGG CTTTGGGTTC CCAATGTACG TGCTATCCAC AGGAAAGAGA
7921 CCTTTTCGAC CTTTTTCCCC TGCTAGGGCA ATTTGCCCTA GCATCTGCTC CGTACATTAG
7981 GAACCGGCGG ATGCTTCGCC CTCGATCAGG TTGCGGTAGC GCATGACTAG GATCGGGCCA
8041 GCCTGCCCCG CCTCCTCCTT CAAATCGTAC TCCGGCAGGT CATTTGACCC GATCAGCTTG
8101 CGCACGGTGA AACAGAACTT CTTGAACTCT CCGGCGCTGC CACTGCGTTC GTAGATCGTC
8161 TTGAACAACC ATCTGGCTTC TGCCTTGCCT GCGGCGCGGC GTGCCAGGCG GTAGAGAAAA
8221 CGGCCGATGC CGGGATCGAT CAAAAAGTAA TCGGGGTGAA CCGTCAGCAC GTCCGGGTTC
8281 TTGCCTTCTG TGATCTCGCG GTACATCCAA TCAGCTAGCT CGATCTCGAT GTACTCCGGC
8341 CGCCCGGTTT CGCTCTTTAC GATCTTGTAG CGGCTAATCA AGGCTTCACC CTCGGATACC
8401 GTCACCAGGC GGCCGTTCTT GGCCTTCTTC GTACGCTGCA TGGCAACGTG CGTGGTGTTT
8461 AACCGAATGC AGGTTTCTAC CAGGTCGTCT TTCTGCTTTC CGCCATCGGC TCGCCGGCAG
8521 AACTTGAGTA CGTCCGCAAC GTGTGGACGG AACACGCGGC CGGGCTTGTC TCCCTTCCCT
8581 TCCCGGTATC GGTTCATGGA TTCGGTTAGA TGGGAAACCG CCATCAGTAC CAGGTCGTAA
8641 TCCCACACAC TGGCCATGCC GGCCGGCCCT GCGGAAACCT CTACGTGCCC GTCTGGAAGC
8701 TCGTAGCGGA TCACCTCGCC AGCTCGTCGG TCACGCTTCG ACAGACGGAA AACGGCCACG
8761 TCCATGATGC TGCGACTATC GCGGGTGCCC ACGTCATAGA GCATCGGAAC GAAAAAATCT
8821 GGTTGCTCGT CGCCCTTGGG CGGCTTCCTA ATCGACGGCG CACCGGCTGC CGGCGGTTGC
8881 CGGGATTCTT TGCGGATTCG ATCAGCGGCC GCTTGCCACG ATTCACCGGG GCGTGCTTCT
8941 GCCTCGATGC GTTGCCGCTG GGCGGCCTGC GCGGCCTTCA ACTTCTCCAC CAGGTCATCA
9001 CCCAGCGCCG CGCCGATTTG TACCGGGCCG GATGGTTTGC GACCGTCACG CCGATTCCTC
9061 GGGCTTGGGG GTTCCAGTGC CATTGCAGGG CCGGCAGACA ACCCAGCCGC TTACGCCTGG
9121 CCAACCGCCC GTTCCTCCAC ACATGGGGCA TTCCACGGCG TCGGTGCCTG GTTGTTCTTG
9181 ATTTTCCATG CCGCCTCCTT TAGCCGCTAA AATTCATCTA CTCATTTATT CATTTGCTCA
9241 TTTACTCTGG TAGCTGCGCG ATGTATTCAG ATAGCAGCTC GGTAATGGTC TTGCCTTGGC
9301 GTACCGCGTA CATCTTCAGC TTGGTGTGAT CCTCCGCCGG CAACTGAAAG TTGACCCGCT
9361 TCATGGCTGG CGTGTCTGCC AGGCTGGCCA ACGTTGCAGC CTTGCTGCTG CGTGCGCTCG
9421 GACGGCCGGC ACTTAGCGTG TTTGTGCTTT TGCTCATTTT CTCTTTACCT CATTAACTCA
9481 AATGAGTTTT GATTTAATTT CAGCGGCCAG CGCCTGGACC TCGCGGGCAG CGTCGCCCTC
9541 GGGTTCTGAT TCAAGAACGG TTGTGCCGGC GGCGGCAGTG CCTGGGTAGC TCACGCGCTG
9601 CGTGATACGG GACTCAAGAA TGGGCAGCTC GTACCCGGCC AGCGCCTCGG CAACCTCACC
9661 GCCGATGCGC GTGCCTTTGA TCGCCCGCGA CACGACAAAG GCCGCTTGTA GCCTTCCATC
9721 CGTGACCTCA ATGCGCTGCT TAACCAGCTC CACCAGGTCG GCGGTGGCCC ATATGTCGTA
9781 AGGGCTTGGC TGCACCGGAA TCAGCACGAA GTCGGCTGCC TTGATCGCGG ACACAGCCAA
9841 GTCCGCCGCC TGGGGCGCTC CGTCGATCAC TACGAAGTCG CGCCGGCCGA TGGCCTTCAC
9901 GTCGCGGTCA ATCGTCGGGC GGTCGATGCC GACAACGGTT AGCGGTTGAT CTTCCCGCAC
9961 GGCCGCCCAA TCGCGGGCAC TGCCCTGGGG ATCGGAATCG ACTAACAGAA CATCGGCCCC
10021 GGCGAGTTGC AGGGCGCGGG CTAGATGGGT TGCGATGGTC GTCTTGCCTG ACCCGCCTTT
10081 CTGGTTAAGT ACAGCGATAA CCTTCATGCG TTCCCCTTGC GTATTTGTTT ATTTACTCAT
10141 CGCATCATAT ACGCAGCGAC CGCATGACGC AAGCTGTTTT ACTCAAATAC ACATCACCTT
10201 TTTAGACGGC GGCGCTCGGT TTCTTCAGCG GCCAAGCTGG CCGGCCAGGC CGCCAGCTTG
10261 GCATCAGACA AACCGGCCAG GATTTCATGC AGCCGCACGG TTGAGACGTG CGCGGGCGGC
10321 TCGAACACGT ACCCGGCCGC GATCATCTCC GCCTCGATCT CTTCGGTAAT GAAAAACGGT
10381 TCGTCCTGGC CGTCCTGGTG CGGTTTCATG CTTGTTCCTC TTGGCGTTCA TTCTCGGCGG
10441 CCGCCAGGGC GTCGGCCTCG GTCAATGCGT CCTCACGGAA GGCACCGCGC CGCCTGGCCT
10501 CGGTGGGCGT CACTTCCTCG CTGCGCTCAA GTGCGCGGTA CAGGGTCGAG CGATGCACGC
10561 CAAGCAGTGC AGCCGCCTCT TTCACGGTGC GGCCTTCCTG GTCGATCAGC TCGCGGGCGT
10621 GCGCGATCTG TGCCGGGGTG AGGGTAGGGC GGGGGCCAAA CTTCACGCCT CGGGCCTTGG
10681 CGGCCTCGCG CCCGCTCCGG GTGCGGTCGA TGATTAGGGA ACGCTCGAAC TCGGCAATGC
10741 CGGCGAACAC GGTCAACACC ATGCGGCCGG CCGGCGTGGT GGTGTCGGCC CACGGCTCTG
10801 CCAGGCTACG CAGGCCCGCG CCGGCCTCCT GGATGCGCTC GGCAATGTCC AGTAGGTCGC
10861 GGGTGCTGCG GGCCAGGCGG TCTAGCCTGG TCACTGTCAC AACGTCGCCA GGGCGTAGGT
10921 GGTCAAGCAT CCTGGCCAGC TCCGGGCGGT CGCGCCTGGT GCCGGTGATC TTCTCGGAAA
10981 ACAGCTTGGT GCAGCCGGCC GCGTGCAGTT CGGCCCGTTG GTTGGTCAAG TCCTGGTCGT
11041 CGGTGCTGAC GCGGGCATAG CCCAGCAGGC CAGCGGCGGC GCTCTTGTTC ATGGCGTAAT
11101 GTCTCCGGTT CTAGTCGCAA GTATTCTACT TTATGCGACT AAAACACGCG ACAAGAAAAC
11161 GCCAGGAAAA GGGCAGGGCG GCAGCCTGTC GCGTAACTTA GGACTTGTGC GACATGTCGT
11221 TTTCAGAAGA CGGCTGCACT GAACGTCAGA AGCCGACTGC ACTATAGCAG CGGAGGGGTT
11281 GGATCAAAGT ACT
```

Supplemental Figure S8. F-SHMT08cΔ+G106A/S107A-CDS, insert size, vector, restriction enzymes, and sequenced positive clones used for cloning.

Gene Name F-SHMT08cΔ+H134A-CDS
Cloning Vector pG2RNAi2
Length (bp) 1430
Cloning Sites AscI-AvrII

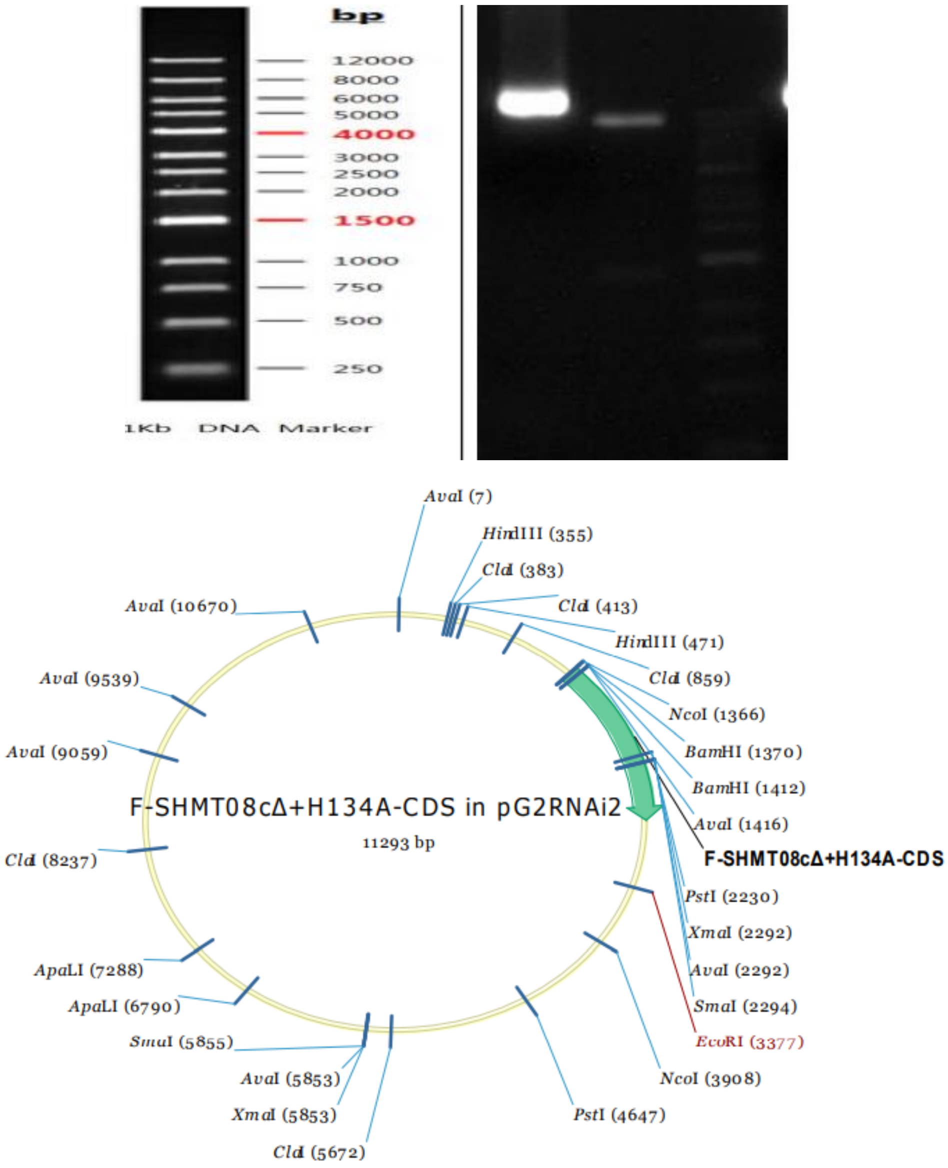

Supplemental Figure S9. F-SHMT08cΔ+H134A-CDS, insert size, vector, restriction enzymes, and sequenced positive clones used for cloning.

Detailed Sequence of the Whole Construct:

```
  1 TTGATCCCGA GGGGAACCCT GTGGTTGGCA TGCACATACA AATGGACGAA CGGATAAACC

 61 TTTTCACGCC CTTTTAAATA TCCGTTATTC TAATAAACGC TCTTTTCTCT TAGGTTTACC

121 CGCCAATATA TCCTGTCAAA CACTGATAGT TTAAACTGAA GGCGGGAAAC GACAATCTGA

181 TCCAAGCTCA AGCTCATTCG CCATTCAGGC TGCGCAACTG TTGGGAAGGG CGATCGGTGC

241 GGGCCTCTTC GCTATTACGC CAGCTGGCGA AAGGGGGATG TGCTGCAAGG CGATTAAGTT

301 GGGTAACGCC AGGGTTTTCC CAGTCACGAC GTTGTAAAAC GACGGCCAGT GCCAAGCTTA

361 TTACCCTGTT ATCCCTAGAT ATCGATTTTG GCTACCTTAA GGTAGCCAAA ATCGATCACT

421 AGTGCGGCCG CGGGCCCAAT ATAACAACGA CGTCGTAACA GATAAAGCGA AGCTTGAAGG

481 TGCATGTGAC TCCGTCAAGA TTACGAAACC GCCAACTACC ACGCAAATTG CAATTCTCAA

541 TTTCCTAGAA GGACTCTCCG AAAATGCATC CAATACCAAA TATTACCCGT GTCATAGGCA

601 CCAAGTGACA CCATACATGA ACACGCGTCA CAATATGACT GGAGAAGGGT TCCACACCTT

661 ATGCTATAAA ACGCCCCACA CCCCTCCTCC TTCCTTCGCA GTTCAATTCC AATATATTCC

721 ATTCTCTCTG TGTATTTCCC TACCTCTCCC TTCAAGGTTA GTCGATTTCT TCTGTTTTTC

781 TTCTTCGTTC TTTCCATGAA TTGTGTATGT TCTTTGATCA ATACGATGTT GATTTGATTG

841 TGTTTTGTTT GGTTTCATCG ATCTTCAATT TTCATAATCA GATTCAGCTT TTATTATCTT
```

Supplemental Figure S9. F-SHMT08cΔ+H134A-CDS, insert size, vector, restriction enzymes, and sequenced positive clones used for cloning.

901 TACAACAACG TCCTTAATTT GATGATTCTT TAATCGTAGA TTTGCTCTAA TTAGAGCTTT
961 TTCATGTCAG ATCCCTTTAC AACAAGCCTT AATTGTTGAT TCATTAATCG TAGATTAGGG
1021 CTTTTTTCAT TGATTACTTC AGATCCGTTA AACGTAACCA TAGATCAGGG CTTTTTCATG
1081 AATTACTTCA GATCCGTTAA ACAACAGCCT TATTTTTTAT ACTTCTGTGG TTTTTCAAGA
1141 AATTGTTCAG ATCCGTTGAC AAAAAGCCTT ATTCGTTGAT TCTATATCGT TTTTCGAGAG
1201 ATATTGCTCA GATCTGTTAG CAACTGCCTT GTTTGTTGAT TCTATTGCCG TGGATTAGGG
1261 TTTTTTTTCA CGAGATTGCT TCAGATCCGT ACTTAAGATT ACGTAATGGA TTTTGATTCT
1321 GATTTATCTG TGATTGTTGA CTCGACAGCG GCCGCACCGG CGCGCCATGG ATCCAGTAAG
1381 CGTGTGGGGT AACACGCCCT TGGCGACGGT GGATCCCGAG ATCCATGACC TCATCGAGAA
1441 GGAGAAGCGC CGTCAATGCC GCGGAATCGA GCTCATCGCC TCCGAGAACT TCACCTCCTT
1501 CGCCGTCATC GAGGCCCTCG GCAGCGCTCT CACGAACAAA TACTCCGAGG GCATGCCGGG
1561 CAACCGCTAC TACGGCGGCA ATGAATACAT CGACCAGATC GAAAACCTCT GCCGCTCACG
1621 CGCCCTCCAA GCCTTCCACC TCGACGCCCA ATCCTGGGGC GTCAACGTCC AGCCCTACTC
1681 CGGCTCCCCG GCCAACTTCG CCGCCTACAC CGCCGTCCTC AACCCCCACG ACCGCATCAT
1741 GGGGCTAGAT CTCCGCTCCG GCGGCGCTCT CACCCACGGC TACTACACCT CCGGCGGAAA
1801 GAAGATCTCC GCCACCTCCA TTTACTTCGA GAGTCTCCCT TACAAGGTAA ACTCCACCAC
1861 CGGCTACATC GACTACGACC GCTTGGAAGA AAAAGCCCTA GACTTCAGGC CAAAACTCAT
1921 AATCTGCGGT GGCAGCGCGT ACCCTCGCGA TTGGGACTAC AAACGTTTCA GGGAAGTCGC
1981 TGATAAGTGC GGAGCATTGC TTCTCTGCGA CATGGCGCAC ACTAGCGGCC TTGTGGCCGC
2041 GCAGGAAGTG AACAGCCCCT TCGAGTATTG CGACATTGTG ACCACCACGA CTCACAAGAG
2101 CTTGCGGGGC CCACGTGCGG GGATGATCTT TTACCGGAAG GGCCCCAAGC CGCCGAAGAA
2161 GGGGCAGCCG GAGAACGCGG TTTATGATTT CGAGGACAAG ATTAACTTCG CGGTGTTCCC
2221 TTCGCTGCAG GGTGGGCCCC ACAACCACCA GATCGGTGCT CTCGCCGTGG CGCTGAAGCA
2281 GGCCGCGTCG CCCGGGTTTA AGGCCTACGC GAAGCAGGTT AAGGCGAACG CCGTTGCGCT
2341 TGGAAAATAC TTGATGGGGA AAGGGTACAG CCTTGTCACT GGCGGAACGG AGAACCATCT
2401 TGTTTTGTGG GATCTGAGAC CTCTTGGATT GACTGGGTAT AAGGTGGAGA AACTCTGTGA
2461 TCTCTGTAAC ATTACTGTTA ACAAGAACGC TGTTTTTGGT GATAGCAGTG CCTTGGCCCC
2521 TGGTGGAGTG CGAATTGGTG CCCCTGCCAT GACTTCTAGG GGTTTGGTTG AAAAAGACTT
2581 TGAGCAGATT GGTGAGTTCC TTCACCGTGC TGTGACTCTC ACACTGGAGA TCCAGAAGGA
2641 GCATGGCAAA CTTCTCAAGG ATTTCAACAA GGGTCTCGTC AACAACAAGG CTATTGAAGA
2701 TCTCAAAGCT GATGTTGAGA AGTTCTCTGC CTTGTTTGAC ATGCCTGGCT TCCTGGTATC
2761 TGAAATGAAG TACAAGGATT AGCCTAGGTT CGAGTATTAT GGCATTGGGA AAACTGTTTT
2821 TCTTGTACCA TTTGTTGTGC TTGTAATTTA CTGTGTTTTT TATTCGGTTT TCGCTATCGA
2881 ACTGTGAAAT GGAAATGGAT GGAGAAGAGT TAATGAATGA TATGGTCCTT TTGTTCATTC
2941 TCAAATTAAT ATTATTTGTT TTTTCTCTTA TTTGTTGTGT GTTGAATTTG AAATTATAAG
3001 AGATATGCAA ACATTTTGTT TTGAGTAAAA ATGTGTCAAA TCGTGGCCTC TAATGACCGA
3061 AGTTAATATG AGGAGTAAAA CACTTGTAGT TGTACCATTA TGCTTATTCA CTAGGCAACA
3121 AATATATTTT CAGACCTAGA AAAGCTGCAA ATGTTACTGA ATACAAGTAT GTCCTCTTGT
3181 GTTTTAGACA TTTATGAACT TTCCTTTATG TAATTTTCCA GAATCCTTGT CAGATTCTAA
3241 TCATTGCTTT ATAATTATAG TTATACTCAT GGATTTGTAG TTGAGTATGA AAATATTTTT
3301 TAATGCATTT TATGACTTGC CAATTGATTG ACAACATGCA TCAATCCGCG GTTATGACTC
3361 TCTTAAGAGA GTCATGAATT CGAGCTTCCA GAAGGTAATT ATCCAAGATG TAGCATCAAG
3421 AATCCAATGT TTACGGGAAA AACTATGGAA GTATTATGTG AGCTCAGCAA GAAGCAGATC
3481 AATATGCGGC ACATATGCAA CCTATGTTCA AAAATGAAGA ATGTACAGAT ACAAGATCCT
3541 ATACTGCCAG AATACGAAGA AGAATACGTA GAAATTGAAA AAGAAGAACC AGGCGAAGAA
3601 AAGAATCTTG AAGACGTAAG CACTGACGAC AACAATGAAA AGAAGAAGAT AAGGTCGGTG
3661 ATTGTGAAAG AGACATAGAG GACACATGTA AGGTGGAAAA TGTAAGGGCG GAAAGTAACC
3721 TTATCACAAA GGAATCTTAT CCCCCACTAC TTATCCTTTT ATATTTTTCC GTGTCATTTT
3781 TGCCCTTGAG TTTTCCTATA TAAGGAACCA AGTTCGGCAT TTGTGAAAAC AAGAAAAAAT
3841 TTGGTGTAAG CTATTTTCTT TGAAGTACTG AGGATACAAC TTCAGAGAAA TTTGTAAGTT
3901 TGTGATCCAT GGTGAGCAAG GGCGAGGAGC TGTTCACCGG GGTGGTGCCC ATCCTGGTCG
3961 AGCTGGACGG CGACGTAAAC GGCCACAAGT TCAGCGTGTC CGGCGAGGGC GAGGGCGATG

Supplemental Figure S9. F-SHMT08cΔ+H134A-CDS, insert size, vector, restriction enzymes, and sequenced positive clones used for cloning.

4021 CCACCTACGG CAAGCTGACC CTGAAGTTCA TCTGCACCAC CGGCAAGCTG CCCGTGCCCT
4081 GGCCCACCCT CGTGACCACC TTCACCTACG GCGTGCAGTG CTTCAGCCGC TACCCCGACC
4141 ACATGAAGCA GCACGACTTC TTCAAGTCCG CCATGCCCGA AGGCTACGTC CAGGAGCGCA
4201 CCATCTTCTT CAAGGACGAC GGCAACTACA AGACCCGCGC CGAGGTGAAG TTCGAGGGCG
4261 ACACCCTGGT GAACCGCATC GAGCTGAAGG GCATCGACTT CAAGGAGGAC GGCAACATCC
4321 TGGGGCACAA GCTGGAGTAC AACTACAACA GCCACAACGT CTATATCATG GCCGACAAGC
4381 AGAAGAACGG CATCAAGGTG AACTTCAAGA TCCGCCACAA CATCGAGGAC GGCAGCGTGC
4441 AGCTCGCCGA CCACTACCAG CAGAACACCC CCATCGGCGA CGGCCCCGTG CTGCTGCCCG
4501 ACAACCACTA CCTGAGCACC CAGTCCGCCC TGAGCAAAGA CCCCAACGAG AAGCGCGATC
4561 ACATGGTCCT GCTGGAGTTC GTGACCGCCG CCGGGATCAC TCACGGCATG GACGAGCTGT
4621 ACAAGTAAAG CGGCCGCCCG GCTGCAGATC GTTCAAACAT TTGGCAATAA AGTTTCTTAA
4681 GATTGAATCC TGTTGCCGGT CTTGCGATGA TTATCATATA ATTTCTGTTG AATTACGTTA
4741 AGCATGTAAT AATTAACATG TAATGCATGA CGTTATTTAT GAGATGGGTT TTTATGATTA
4801 GAGTCCCGCA ATTATACATT TAATACGCGA TAGAAAACAA AATATAGCGC GCAAACTAGG
4861 ATAAATTATC GCGCGCGGTG TCATCTATGT TACTAGATCC GATGATAAGC TGTCAAACAT
4921 GAGAATTAAT TCGTAATCAT GTCATAGCTG TTTCCTGTGT GAAATTGTTA TCCGCTCACA
4981 ATTCCACACA ACATACGAGC CGGAAGCATA AAGTGTAAAG CCTGGGGTGC CTAATGAGTG
5041 AGCTAACTCA CATTAATTGC GTTGCGCTCA CTGCCCGCTT TCCAGTCGGG AAACCTGTCG
5101 TGCCAGCTGC ATTAATGAAT CGGCCAACGC GCGGGGAGAG GCGGTTTGCG TATTGGCTAG
5161 AGCAATTCGG CGTTAATTCA GTACATTAAA AACGTCCGCA ATGTGTTATT AAGTTGTCTA
5221 AGCGTCAATT TGTTTACACC ACAATATATC CTGCCACCAG CCAGCCAACA GCTCCCCGAC
5281 CGGCAGCTCG GCACAAAATC ACCACTCGAT ACAGGCAGCC CATCAGTCCG GGACGGCGTC
5341 AGCGGGAGAG CCGTTGTAAG GCGGCAGACT TTGCTCATGT TACCGATGCT ATTCGGAAGA
5401 ACGGCAACTA AGCTGCCGGG TTTGAAACAC GGATGATCTC GCGGAGGGTA GCATGTTGAT
5461 TGTAACGATG ACAGAGCGTT GCTGCCTGTG ATCCAGATCA TGAACAATAA AACTGTCTGC
5521 TTACATAAAC AGTAATACAA GGGGTGTTAT GAGCCATATT CAACGGGAAA CGTCTTGCTC

5581 TAGGCCGCGA TTAAATTCCA ACATGGATGC TGATTTATAT GGGTATAAAT GGGCTCGCGA
5641 TAATGTCGGG CAATCAGGTG CGACAATCTA TCGATTGTAT GGGAAGCCCG ATGCGCCAGA
5701 GTTGTTTCTG AAACATGGCA AAGGTAGCGT TGCCAATGAT GTTACAGATG AGATGGTCAG
5761 ACTAAACTGG CTGACGGAAT TTATGCCTCT TCCGACCATC AAGCATTTTA TCCGTACTCC
5821 TGATGATGCA TGGTTACTCA CCACTGCGAT CCCCGGGAAA ACAGCATTCC AGGTATTAGA
5881 AGAATATCCT GATTCAGGTG AAAATATTGT TGATGCGCTG GCAGTGTTCC TGCGCCGGTT
5941 GCATTCGATT CCTGTTTGTA ATTGTCCTTT TAACAGCGAT CGCGTATTTC GTCTCGCTCA
6001 GGCGCAATCA CGAATGAATA ACGGTTTGGT TGATGCGAGT GATTTTGATG ACGAGCGTAA
6061 TGGCTGGCCT GTTGAACAAG TCTGGAAAGA AATGCATAAA CTTTTGCCAT TCTCACCGGA
6121 TTCAGTCGTC ACTCATGGTG ATTTCTCACT TGATAACCTT ATTTTTGACG AGGGGAAATT
6181 AATAGGTTGT ATTGATGTTG GACGAGTCGG AATCGCAGAC CGATACCAGG ATCTTGCCAT
6241 CCTATGGAAC TGCCTCGGTG AGTTTTCTCC TTCATTACAG AAACGGCTTT TTCAAAAATA
6301 TGGTATTGAT AATCCTGATA TGAATAAATT GCAGTTTCAT TTGATGCTCG ATGAGTTTTT
6361 CTAAGAATTA ATTCATGATC CTGCATGACC AAAATCCCTT AACGTGAGTT TTCGTTCCAC
6421 TGAGCGTCAG ACCCCGTAGA AAAGATCAAA GGATCTTCTT GAGATCCTTT TTTTCTGCGC
6481 GTAATCTGCT GCTTGCAAAC AAAAAAACCA CCGCTACCAG CGGTGGTTTG TTTGCCGGAT
6541 CAAGAGCTAC CAACTCTTTT TCCGAAGGTA ACTGGCTTCA GCAGAGCGCA GATACCAAAT
6601 ACTGTCCTTC TAGTGTAGCC GTAGTTAGGC CACCACTTCA AGAACTCTGT AGCACCGCCT
6661 ACATACCTCG CTCTGCTAAT CCTGTTACCA GTGGCTGCTG CCAGTGGCGA TAAGTCGTGT
6721 CTTACCGGGT TGGACTCAAG ACGATAGTTA CCGGATAAGG CGCAGCGGTC GGGCTGAACG
6781 GGGGGTTCGT GCACACAGCC CAGCTTGGAG CGAACGACCT ACACCGAACT GAGATACCTA
6841 CAGCGTGAGC TATGAGAAAG CGCCACGCTT CCCGAAGGGA GAAAGGCGGA CAGGTATCCG
6901 GTAAGCGGCA GGGTCGGAAC AGGAGAGCGC ACGAGGGAGC TTCCAGGGGG AAACGCCTGG
6961 TATCTTTATA GTCCTGTCGG GTTTCGCCAC CTCTGACTTG AGCGTCGATT TTTGTGATGC
7021 TCGTCAGGGG GGCGGAGCCT ATGGAAAAAC GCCAGCAACG CGGCCTTTTT ACGGTTCCTG
7081 GCCTTTTGCT GGCCTTTTGC TCACATGTTC TTTCCTGCGT TATCCCCTGA TTCTGTGGAT

Supplemental Figure S9. F-SHMT08cΔ+H134A-CDS, insert size, vector, restriction enzymes, and sequenced positive clones used for cloning.

```
7141 AACCGTATTA CCGCCTTTGA GTGAGCTGAT ACCGCTCGCC GCAGCCGAAC GACCGAGCGC
7201 AGCGAGTCAG TGAGCGAGGA AGCGGAAGAG CGCCTGATGC GGTATTTTCT CCTTACGCAT
7261 CTGTGCGGTA TTTCACACCG CATATGGTGC ACTCTCAGTA CAATCTGCTC TGATGCCGCA
7321 TAGTTAAGCC AGTATACACT CCGCTATCGC TACGTGACTG GGTCATGGCT GCGCCCCGAC
7381 ACCCGCCAAC ACCCGCTGAC GCGCCCTGAC GGGCTTGTCT GCTCCCGGCA TCCGCTTACA
7441 GACAAGCTGT GACCGTCTCC GGGAGCTGCA TGTGTCAGAG GTTTTCACCG TCATCACCGA
7501 AACGCGCGAG GCAGGGTGCC TTGATGTGGG CGCCGGCGGT CGAGTGGCGA CGGCGCGGCT
7561 TGTCCGCGCC CTGGTAGATT GCCTGGCCGT AGGCCAGCCA TTTTTGAGCG GCCAGCGGCC
7621 GCGATAGGCC GACGCGAAGC GGCGGGGCGT AGGGAGCGCA GCGACCGAAG GGTAGGCGCT
7681 TTTTGCAGCT CTTCGGCTGT GCGCTGGCCA GACAGTTATG CACAGGCCAG GCGGGTTTTA
7741 AGAGTTTTAA TAAGTTTTAA AGAGTTTTAG GCGGAAAAAT CGCCTTTTTT CTCTTTTATA
7801 TCAGTCACTT ACATGTGTGA CCGGTTCCCA ATGTACGGCT TTGGGTTCCC AATGTACGGG
7861 TTCCGGTTCC CAATGTACGG CTTTGGGTTC CCAATGTACG TGCTATCCAC AGGAAAGAGA
7921 CCTTTTCGAC CTTTTTCCCC TGCTAGGGCA ATTTGCCCTA GCATCTGCTC CGTACATTAG
7981 GAACCGGCGG ATGCTTCGCC CTCGATCAGG TTGCGGTAGC GCATGACTAG GATCGGGCCA
8041 GCCTGCCCCG CCTCCTCCTT CAAATCGTAC TCCGGCAGGT CATTTGACCC GATCAGCTTG
8101 CGCACGGTGA AACAGAACTT CTTGAACTCT CCGGCGCTGC CACTGCGTTC GTAGATCGTC
8161 TTGAACAACC ATCTGGCTTC TGCCTTGCCT GCGGCGCGGC GTGCCAGGCG GTAGAGAAAA
8221 CGGCCGATGC CGGGATCGAT CAAAAAGTAA TCGGGGTGAA CCGTCAGCAC GTCCGGGTTC
8281 TTGCCTTCTG TGATCTCGCG GTACATCCAA TCAGCTAGCT CGATCTCGAT GTACTCCGGC
8341 CGCCCGGTTT CGCTCTTTAC GATCTTGTAG CGGCTAATCA AGGCTTCACC CTCGGATACC
8401 GTCACCAGGC GGCCGTTCTT GGCCTTCTTC GTACGCTGCA TGGCAACGTG CGTGGTGTTT
8461 AACCGAATGC AGGTTTCTAC CAGGTCGTCT TTCTGCTTTC CGCCATCGGC TCGCCGGCAG
8521 AACTTGAGTA CGTCCGCAAC GTGTGGACGG AACACGCGGC CGGGCTTGTC TCCCTTCCCT
8581 TCCCGGTATC GGTTCATGGA TTCGGTTAGA TGGGAAACCG CCATCAGTAC CAGGTCGTAA
8641 TCCCACACAC TGGCCATGCC GGCCGGCCCT GCGGAAACCT CTACGTGCCC GTCTGGAAGC
8701 TCGTAGCGGA TCACCTCGCC AGCTCGTCGG TCACGCTTCG ACAGACGGAA AACGGCCACG
8761 TCCATGATGC TGCGACTATC GCGGGTGCCC ACGTCATAGA GCATCGGAAC GAAAAAATCT
8821 GGTTGCTCGT CGCCCTTGGG CGGCTTCCTA ATCGACGGCG CACCGGCTGC CGGCGGTTGC
8881 CGGGATTCTT TGCGGATTCG ATCAGCGGCC GCTTGCCACG ATTCACCGGG GCGTGCTTCT
8941 GCCTCGATGC GTTGCCGCTG GGCGGCCTGC GCGGCCTTCA ACTTCTCCAC CAGGTCATCA
9001 CCCAGCGCCG CGCCGATTTG TACCGGGCCG GATGGTTTGC GACCGTCACG CCGATTCCTC
9061 GGGCTTGGGG GTTCCAGTGC CATTGCAGGG CCGGCAGACA ACCCAGCCGC TTACGCCTGG
9121 CCAACCGCCC GTTCCTCCAC ACATGGGGCA TTCCACGGCG TCGGTGCCTG GTTGTTCTTG
9181 ATTTTCCATG CCGCCTCCTT TAGCCGCTAA AATTCATCTA CTCATTTATT CATTTGCTCA
9241 TTTACTCTGG TAGCTGCGCG ATGTATTCAG ATAGCAGCTC GGTAATGGTC TTGCCTTGGC
9301 GTACCGCGTA CATCTTCAGC TTGGTGTGAT CCTCCGCCGG CAACTGAAAG TTGACCCGCT
9361 TCATGGCTGG CGTGTCTGCC AGGCTGGCCA ACGTTGCAGC CTTGCTGCTG CGTGCGCTCG
9421 GACGGCCGGC ACTTAGCGTG TTTGTGCTTT TGCTCATTTT CTCTTTACCT CATTAACTCA
9481 AATGAGTTTT GATTTAATTT CAGCGGCCAG CGCCTGGACC TCGCGGGCAG CGTCGCCCTC
9541 GGGTTCTGAT TCAAGAACGG TTGTGCCGGC GGCGGCAGTG CCTGGGTAGC TCACGCGCTG
9601 CGTGATACGG GACTCAAGAA TGGGCAGCTC GTACCCGGCC AGCGCCTCGG CAACCTCACC
9661 GCCGATGCGC GTGCCTTTGA TCGCCCGCGA CACGACAAAG GCCGCTTGTA GCCTTCCATC
9721 CGTGACCTCA ATGCGCTGCT TAACCAGCTC CACCAGGTCG GCGGTGGCCC ATATGTCGTA
9781 AGGGCTTGGC TGCACCGGAA TCAGCACGAA GTCGGCTGCC TTGATCGCGG ACACAGCCAA
9841 GTCCGCCGCC TGGGGCGCTC CGTCGATCAC TACGAAGTCG CGCCGGCCGA TGGCCTTCAC
9901 GTCGCGGTCA ATCGTCGGGC GGTCGATGCC GACAACGGTT AGCGGTTGAT CTTCCCGCAC
9961 GGCCGCCCAA TCGCGGGCAC TGCCCTGGGG ATCGGAATCG ACTAACAGAA CATCGGCCCC
10021 GGCGAGTTGC AGGGCGCGGG CTAGATGGGT TGCGATGGTC GTCTTGCCTG ACCCGCCTTT
10081 CTGGTTAAGT ACAGCGATAA CCTTCATGCG TTCCCCTTGC GTATTTGTTT ATTTACTCAT
10141 CGCATCATAT ACGCAGCGAC CGCATGACGC AAGCTGTTTT ACTCAAATAC ACATCACCTT
10201 TTTAGACGGC GGCGCTCGGT TTCTTCAGCG GCCAAGCTGG CCGGCCAGGC CGCCAGCTTG
```

Supplemental Figure S9. F-SHMT08cΔ+H134A-CDS, insert size, vector, restriction enzymes, and sequenced positive clones used for cloning.

```
10261 GCATCAGACA AACCGGCCAG GATTTCATGC AGCCGCACGG TTGAGACGTG CGCGGGCGGC
10321 TCGAACACGT ACCCGGCCGC GATCATCTCC GCCTCGATCT CTTCGGTAAT GAAAAACGGT
10381 TCGTCCTGGC CGTCCTGGTG CGGTTTCATG CTTGTTCCTC TTGGCGTTCA TTCTCGGCGG
10441 CCGCCAGGGC GTCGGCCTCG GTCAATGCGT CCTCACGGAA GGCACCGCGC CGCCTGGCCT
10501 CGGTGGGCGT CACTTCCTCG CTGCGCTCAA GTGCGCGGTA CAGGGTCGAG CGATGCACGC
10561 CAAGCAGTGC AGCCGCCTCT TTCACGGTGC GGCCTTCCTG GTCGATCAGC TCGCGGGCGT
10621 GCGCGATCTG TGCCGGGGTG AGGGTAGGGC GGGGGCCAAA CTTCACGCCT CGGGCCTTGG
10681 CGGCCTCGCG CCCGCTCCGG GTGCGGTCGA TGATTAGGGA ACGCTCGAAC TCGGCAATGC
10741 CGGCGAACAC GGTCAACACC ATGCGGCCGG CCGGCGTGGT GGTGTCGGCC CACGGCTCTG
10801 CCAGGCTACG CAGGCCCGCG CCGGCCTCCT GGATGCGCTC GGCAATGTCC AGTAGGTCGC
10861 GGGTGCTGCG GGCCAGGCGG TCTAGCCTGG TCACTGTCAC AACGTCGCCA GGGCGTAGGT
10921 GGTCAAGCAT CCTGGCCAGC TCCGGGCGGT CGCGCCTGGT GCCGGTGATC TTCTCGGAAA
10981 ACAGCTTGGT GCAGCCGGCC GCGTGCAGTT CGGCCCGTTG GTTGGTCAAG TCCTGGTCGT
11041 CGGTGCTGAC GCGGGCATAG CCCAGCAGGC CAGCGGCGGC GCTCTTGTTC ATGGCGTAAT
11101 GTCTCCGGTT CTAGTCGCAA GTATTCTACT TTATGCGACT AAAACACGCG ACAAGAAAAC
11161 GCCAGGAAAA GGGCAGGGCG GCAGCCTGTC GCGTAACTTA GGACTTGTGC GACATGTCGT
11221 TTTCAGAAGA CGGCTGCACT GAACGTCAGA AGCCGACTGC ACTATAGCAG CGGAGGGGTT
11281 GGATCAAAGT ACT
```

Supplemental Figure S9. F-SHMT08cΔ+H134A-CDS, insert size, vector, restriction enzymes, and sequenced positive clones used for cloning.

Gene Name F-SHMT08cΔ+F284A-CDS
Cloning Vector pG2RNAi2
Length (bp) 1430
Cloning Sites AscI-AvrII

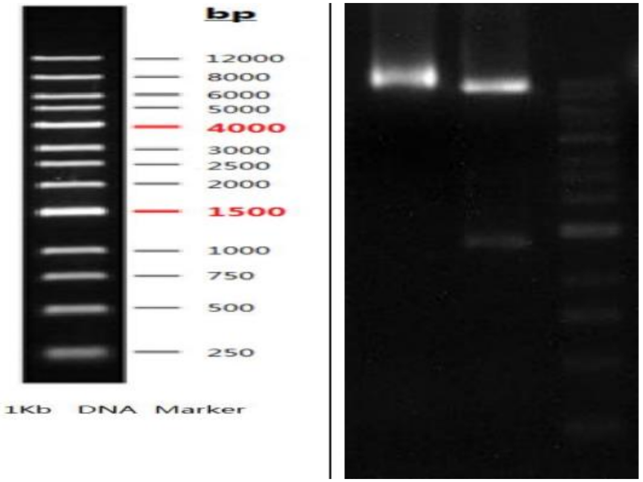

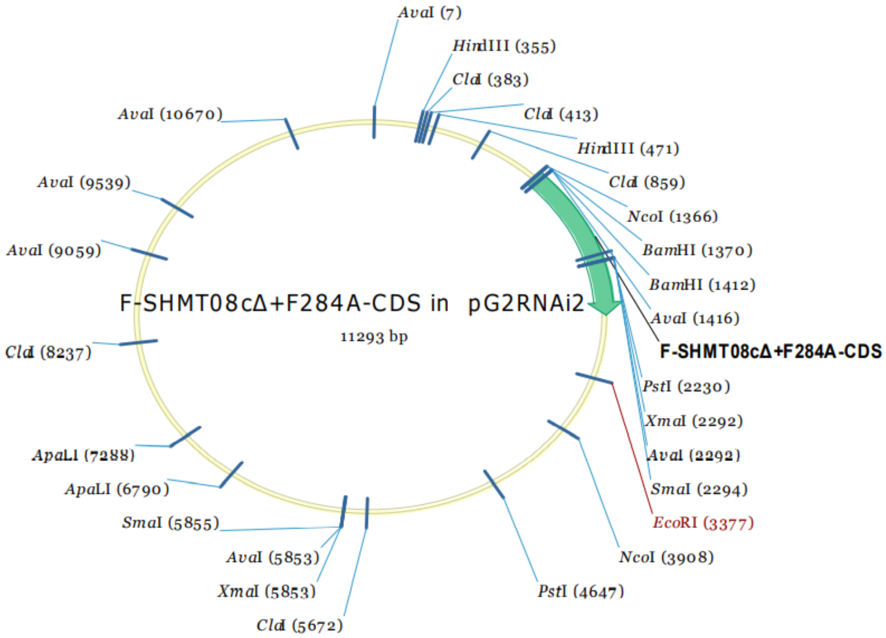

Supplemental Figure S10. F-SHMT08cΔ+F284A-CDS, insert size, vector, restriction enzymes, and sequenced positive clones used for cloning.

Detailed Sequence of the Whole Construct:

1 TTGATCCCGA GGGGAACCCT GTGGTTGGCA TGCACATACA AATGGACGAA CGGATAAACC

61 TTTTCACGCC CTTTTAAATA TCCGTTATTC TAATAAACGC TCTTTTCTCT TAGGTTTACC

121 CGCCAATATA TCCTGTCAAA CACTGATAGT TTAAACTGAA GGCGGGAAAC GACAATCTGA

181 TCCAAGCTCA AGCTCATTCG CCATTCAGGC TGCGCAACTG TTGGGAAGGG CGATCGGTGC

241 GGGCCTCTTC GCTATTACGC CAGCTGGCGA AAGGGGGATG TGCTGCAAGG CGATTAAGTT

301 GGGTAACGCC AGGGTTTTCC CAGTCACGAC GTTGTAAAAC GACGGCCAGT GCCAAGCTTA

361 TTACCCTGTT ATCCCTAGAT ATCGATTTTG GCTACCTTAA GGTAGCCAAA ATCGATCACT

421 AGTGCGGCCG CGGGCCCAAT ATAACAACGA CGTCGTAACA GATAAAGCGA AGCTTGAAGG

481 TGCATGTGAC TCCGTCAAGA TTACGAAACC GCCAACTACC ACGCAAATTG CAATTCTCAA

541 TTTCCTAGAA GGACTCTCCG AAAATGCATC CAATACCAAA TATTACCCGT GTCATAGGCA

601 CCAAGTGACA CCATACATGA ACACGCGTCA CAATATGACT GGAGAAGGGT TCCACACCTT

661 ATGCTATAAA ACGCCCCACA CCCCTCCTCC TTCCTTCGCA GTTCAATTCC AATATATTCC

721 ATTCTCTCTG TGTATTTCCC TACCTCTCCC TTCAAGGTTA GTCGATTTCT TCTGTTTTTC

781 TTCTTCGTTC TTTCCATGAA TTGTGTATGT TCTTTGATCA ATACGATGTT GATTTGATTG

841 TGTTTTGTTT GGTTTCATCG ATCTTCAATT TTCATAATCA GATTCAGCTT TTATTATCTT

Supplemental Figure S10. F-SHMT08cΔ+F284A-CDS, insert size, vector, restriction enzymes, and sequenced positive clones used for cloning.

```
 901 TACAACAACG TCCTTAATTT GATGATTCTT TAATCGTAGA TTTGCTCTAA TTAGAGCTTT
 961 TTCATGTCAG ATCCCTTTAC AACAAGCCTT AATTGTTGAT TCATTAATCG TAGATTAGGG
1021 CTTTTTTCAT TGATTACTTC AGATCCGTTA AACGTAACCA TAGATCAGGG CTTTTTCATG
1081 AATTACTTCA GATCCGTTAA ACAACAGCCT TATTTTTTAT ACTTCTGTGG TTTTTCAAGA
1141 AATTGTTCAG ATCCGTTGAC AAAAAGCCTT ATTCGTTGAT TCTATATCGT TTTTCGAGAG
1201 ATATTGCTCA GATCTGTTAG CAACTGCCTT GTTTGTTGAT TCTATTGCCG TGGATTAGGG
1261 TTTTTTTTCA CGAGATTGCT TCAGATCCGT ACTTAAGATT ACGTAATGGA TTTTGATTCT
1321 GATTTATCTG TGATTGTTGA CTCGACAGCG GCCGCACCGG CGCGCCATGG ATCCAGTAAG
1381 CGTGTGGGGT AACACGCCCT TGGCGACGGT GGATCCCGAG ATCCATGACC TCATCGAGAA
1441 GGAGAAGCGC CGTCAATGCC GCGGAATCGA GCTCATCGCC TCCGAGAACT TCACCTCCTT
1501 CGCCGTCATC GAGGCCCTCG GCAGCGCTCT CACGAACAAA TACTCCGAGG GCATGCCGGG
1561 CAACCGCTAC TACGGCGGCA ATGAATACAT CGACCAGATC GAAAACCTCT GCCGCTCACG
1621 CGCCCTCCAA GCCTTCCACC TCGACGCCCA ATCCTGGGGC GTCAACGTCC AGCCCTACTC
1681 CGGCTCCCCG GCCAACTTCG CCGCCTACAC CGCCGTCCTC AACCCCCACG ACCGCATCAT
1741 GGGGCTAGAT CTCCGCTCCG GCGGCCACCT CACCCACGGC TACTACACCT CCGGCGGAAA
1801 GAAGATCTCC GCCACCTCCA TTTACTTCGA GAGTCTCCCT TACAAGGTAA ACTCCACCAC
1861 CGGCTACATC GACTACGACC GCTTGGAAGA AAAAGCCCTA GACTTCAGGC CAAAACTCAT
1921 AATCTGCGGT GGCAGCGCGT ACCCTCGCGA TTGGGACTAC AAACGTTTCA GGGAAGTCGC
1981 TGATAAGTGC GGAGCATTGC TTCTCTGCGA CATGGCGCAC ACTAGCGGCC TTGTGGCCGC
2041 GCAGGAAGTG AACAGCCCCT TCGAGTATTG CGACATTGTG ACCACCACGA CTCACAAGAG
2101 CTTGCGGGGC CCACGTGCGG GGATGATCTT TTACCGGAAG GGCCCCAAGC CGCCGAAGAA
2161 GGGGCAGCCG GAGAACGCGG TTTATGATTT CGAGGACAAG ATTAACTTCG CGGTGGCTCC
2221 TTCGCTGCAG GGTGGGCCCC ACAACCACCA GATCGGTGCT CTCGCCGTGG CGCTGAAGCA
2281 GGCCGCGTCG CCCGGGTTTA AGGCCTACGC GAAGCAGGTT AAGGCGAACG CCGTTGCGCT
2341 TGGAAAATAC TTGATGGGGA AAGGGTACAG CCTTGTCACT GGCGGAACGG AGAACCATCT
2401 TGTTTTGTGG GATCTGAGAC CTCTTGGATT GACTGGGTAT AAGGTGGAGA AACTCTGTGA
2461 TCTCTGTAAC ATTACTGTTA ACAAGAACGC TGTTTTTGGT GATAGCAGTG CCTTGGCCCC
2521 TGGTGGAGTG CGAATTGGTG CCCCTGCCAT GACTTCTAGG GGTTTGGTTG AAAAAGACTT
2581 TGAGCAGATT GGTGAGTTCC TTCACCGTGC TGTGACTCTC ACACTGGAGA TCCAGAAGGA
2641 GCATGGCAAA CTTCTCAAGG ATTTCAACAA GGGTCTCGTC AACAACAAGG CTATTGAAGA
2701 TCTCAAAGCT GATGTTGAGA AGTTCTCTGC CTTGTTTGAC ATGCCTGGCT TCCTGGTATC
2761 TGAAATGAAG TACAAGGATT AGCCTAGGTT CGAGTATTAT GGCATTGGGA AAACTGTTTT
2821 TCTTGTACCA TTTGTTGTGC TTGTAATTTA CTGTGTTTTT TATTCGGTTT TCGCTATCGA
2881 ACTGTGAAAT GGAAATGGAT GGAGAAGAGT TAATGAATGA TATGGTCCTT TTGTTCATTC
2941 TCAAATTAAT ATTATTTGTT TTTTCTCTTA TTTGTTGTGT GTTGAATTTG AAATTATAAG
3001 AGATATGCAA ACATTTTGTT TTGAGTAAAA ATGTGTCAAA TCGTGGCCTC TAATGACCGA
3061 AGTTAATATG AGGAGTAAAA CACTTGTAGT TGTACCATTA TGCTTATTCA CTAGGCAACA
3121 AATATATTTT CAGACCTAGA AAAGCTGCAA ATGTTACTGA ATACAAGTAT GTCCTCTTGT
3181 GTTTTAGACA TTTATGAACT TTCCTTTATG TAATTTTCCA GAATCCTTGT CAGATTCTAA
3241 TCATTGCTTT ATAATTATAG TTATACTCAT GGATTTGTAG TTGAGTATGA AAATATTTTT
3301 TAATGCATTT TATGACTTGC CAATTGATTG ACAACATGCA TCAATCCGCG GTTATGACTC
3361 TCTTAAGAGA GTCATGAATT CGAGCTTCCA GAAGGTAATT ATCCAAGATG TAGCATCAAG
3421 AATCCAATGT TTACGGGAAA AACTATGGAA GTATTATGTG AGCTCAGCAA GAAGCAGATC
3481 AATATGCGGC ACATATGCAA CCTATGTTCA AAAATGAAGA ATGTACAGAT ACAAGATCCT
3541 ATACTGCCAG AATACGAAGA AGAATACGTA GAAATTGAAA AAGAAGAACC AGGCGAAGAA
3601 AAGAATCTTG AAGACGTAAG CACTGACGAC AACAATGAAA AGAAGAAGAT AAGGTCGGTG
3661 ATTGTGAAAG AGACATAGAG GACACATGTA AGGTGGAAAA TGTAAGGGCG GAAAGTAACC
3721 TTATCACAAA GGAATCTTAT CCCCCACTAC TTATCCTTTT ATATTTTTCC GTGTCATTTT
3781 TGCCCTTGAG TTTTCCTATA TAAGGAACCA AGTTCGGCAT TTGTGAAAAC AAGAAAAAAT
3841 TTGGTGTAAG CTATTTTCTT TGAAGTACTG AGGATACAAC TTCAGAGAAA TTTGTAAGTT
3901 TGTGATCCAT GGTGAGCAAG GGCGAGGAGC TGTTCACCGG GGTGGTGCCC ATCCTGGTCG
3961 AGCTGGACGG CGACGTAAAC GGCCACAAGT TCAGCGTGTC CGGCGAGGGC GAGGGCGATG
```

Supplemental Figure S10. F-SHMT08cΔ+F284A-CDS, insert size, vector, restriction enzymes, and sequenced positive clones used for cloning.

```
4021 CCACCTACGG CAAGCTGACC CTGAAGTTCA TCTGCACCAC CGGCAAGCTG CCCGTGCCCT
4081 GGCCCACCCT CGTGACCACC TTCACCTACG GCGTGCAGTG CTTCAGCCGC TACCCCGACC
4141 ACATGAAGCA GCACGACTTC TTCAAGTCCG CCATGCCCGA AGGCTACGTC CAGGAGCGCA
4201 CCATCTTCTT CAAGGACGAC GGCAACTACA AGACCCGCGC CGAGGTGAAG TTCGAGGGCG
4261 ACACCCTGGT GAACCGCATC GAGCTGAAGG GCATCGACTT CAAGGAGGAC GGCAACATCC
4321 TGGGGCACAA GCTGGAGTAC AACTACAACA GCCACAACGT CTATATCATG GCCGACAAGC
4381 AGAAGAACGG CATCAAGGTG AACTTCAAGA TCCGCCACAA CATCGAGGAC GGCAGCGTGC
4441 AGCTCGCCGA CCACTACCAG CAGAACACCC CCATCGGCGA CGGCCCCGTG CTGCTGCCCG
4501 ACAACCACTA CCTGAGCACC CAGTCCGCCC TGAGCAAAGA CCCCAACGAG AAGCGCGATC
4561 ACATGGTCCT GCTGGAGTTC GTGACCGCCG CCGGGATCAC TCACGGCATG GACGAGCTGT
4621 ACAAGTAAAG CGGCCGCCCG GCTGCAGATC GTTCAAACAT TTGGCAATAA AGTTTCTTAA
4681 GATTGAATCC TGTTGCCGGT CTTGCGATGA TTATCATATA ATTTCTGTTG AATTACGTTA
4741 AGCATGTAAT AATTAACATG TAATGCATGA CGTTATTTAT GAGATGGGTT TTTATGATTA
4801 GAGTCCCGCA ATTATACATT TAATACGCGA TAGAAAACAA AATATAGCGC GCAAACTAGG
4861 ATAAATTATC GCGCGCGGTG TCATCTATGT TACTAGATCC GATGATAAGC TGTCAAACAT
4921 GAGAATTAAT TCGTAATCAT GTCATAGCTG TTTCCTGTGT GAAATTGTTA TCCGCTCACA
4981 ATTCCACACA ACATACGAGC CGGAAGCATA AAGTGTAAAG CCTGGGGTGC CTAATGAGTG
5041 AGCTAACTCA CATTAATTGC GTTGCGCTCA CTGCCCGCTT TCCAGTCGGG AAACCTGTCG
5101 TGCCAGCTGC ATTAATGAAT CGGCCAACGC GCGGGGAGAG GCGGTTTGCG TATTGGCTAG
5161 AGCAATTCGG CGTTAATTCA GTACATTAAA AACGTCCGCA ATGTGTTATT AAGTTGTCTA
5221 AGCGTCAATT TGTTTACACC ACAATATATC CTGCCACCAG CCAGCCAACA GCTCCCCGAC
5281 CGGCAGCTCG GCACAAAATC ACCACTCGAT ACAGGCAGCC CATCAGTCCG GGACGGCGTC
5341 AGCGGGAGAG CCGTTGTAAG GCGGCAGACT TTGCTCATGT TACCGATGCT ATTCGGAAGA
5401 ACGGCAACTA AGCTGCCGGG TTTGAAACAC GGATGATCTC GCGGAGGGTA GCATGTTGAT
5461 TGTAACGATG ACAGAGCGTT GCTGCCTGTG ATCCAGATCA TGAACAATAA AACTGTCTGC
5521 TTACATAAAC AGTAATACAA GGGGTGTTAT GAGCCATATT CAACGGGAAA CGTCTTGCTC
5581 TAGGCCGCGA TTAAATTCCA ACATGGATGC TGATTTATAT GGGTATAAAT GGGCTCGCGA
5641 TAATGTCGGG CAATCAGGTG CGACAATCTA TCGATTGTAT GGGAAGCCCG ATGCGCCAGA
5701 GTTGTTTCTG AAACATGGCA AAGGTAGCGT TGCCAATGAT GTTACAGATG AGATGGTCAG
5761 ACTAAACTGG CTGACGGAAT TTATGCCTCT TCCGACCATC AAGCATTTTA TCCGTACTCC
5821 TGATGATGCA TGGTTACTCA CCACTGCGAT CCCCGGGAAA ACAGCATTCC AGGTATTAGA
5881 AGAATATCCT GATTCAGGTG AAAATATTGT TGATGCGCTG GCAGTGTTCC TGCGCCGGTT
5941 GCATTCGATT CCTGTTTGTA ATTGTCCTTT TAACAGCGAT CGCGTATTTC GTCTCGCTCA
6001 GGCGCAATCA CGAATGAATA ACGGTTTGGT TGATGCGAGT GATTTTGATG ACGAGCGTAA
6061 TGGCTGGCCT GTTGAACAAG TCTGGAAAGA AATGCATAAA CTTTTGCCAT TCTCACCGGA
6121 TTCAGTCGTC ACTCATGGTG ATTTCTCACT TGATAACCTT ATTTTTGACG AGGGGAAATT
6181 AATAGGTTGT ATTGATGTTG GACGAGTCGG AATCGCAGAC CGATACCAGG ATCTTGCCAT
6241 CCTATGGAAC TGCCTCGGTG AGTTTTCTCC TTCATTACAG AAACGGCTTT TTCAAAAATA
6301 TGGTATTGAT AATCCTGATA TGAATAAATT GCAGTTTCAT TTGATGCTCG ATGAGTTTTT
6361 CTAAGAATTA ATTCATGATC CTGCATGACC AAAATCCCTT AACGTGAGTT TTCGTTCCAC
6421 TGAGCGTCAG ACCCCGTAGA AAAGATCAAA GGATCTTCTT GAGATCCTTT TTTTCTGCGC
6481 GTAATCTGCT GCTTGCAAAC AAAAAAACCA CCGCTACCAG CGGTGGTTTG TTTGCCGGAT
6541 CAAGAGCTAC CAACTCTTTT TCCGAAGGTA ACTGGCTTCA GCAGAGCGCA GATACCAAAT
6601 ACTGTCCTTC TAGTGTAGCC GTAGTTAGGC CACCACTTCA AGAACTCTGT AGCACCGCCT
6661 ACATACCTCG CTCTGCTAAT CCTGTTACCA GTGGCTGCTG CCAGTGGCGA TAAGTCGTGT
6721 CTTACCGGGT TGGACTCAAG ACGATAGTTA CCGGATAAGG CGCAGCGGTC GGGCTGAACG
6781 GGGGGTTCGT GCACACAGCC CAGCTTGGAG CGAACGACCT ACACCGAACT GAGATACCTA
6841 CAGCGTGAGC TATGAGAAAG CGCCACGCTT CCCGAAGGGA GAAAGGCGGA CAGGTATCCG
6901 GTAAGCGGCA GGGTCGGAAC AGGAGAGCGC ACGAGGGAGC TTCCAGGGGG AAACGCCTGG
6961 TATCTTTATA GTCCTGTCGG GTTTCGCCAC CTCTGACTTG AGCGTCGATT TTTGTGATGC
7021 TCGTCAGGGG GGCGGAGCCT ATGGAAAAAC GCCAGCAACG CGGCCTTTTT ACGGTTCCTG
7081 GCCTTTTGCT GGCCTTTTGC TCACATGTTC TTTCCTGCGT TATCCCCTGA TTCTGTGGAT
```

Supplemental Figure S10. F-SHMT08cΔ+F284A-CDS, insert size, vector, restriction enzymes, and sequenced positive clones used for cloning.

7141 AACCGTATTA CCGCCTTTGA GTGAGCTGAT ACCGCTCGCC GCAGCCGAAC GACCGAGCGC

7201 AGCGAGTCAG TGAGCGAGGA AGCGGAAGAG CGCCTGATGC GGTATTTTCT CCTTACGCAT

7261 CTGTGCGGTA TTTCACACCG CATATGGTGC ACTCTCAGTA CAATCTGCTC TGATGCCGCA

7321 TAGTTAAGCC AGTATACACT CCGCTATCGC TACGTGACTG GGTCATGGCT GCGCCCCGAC

7381 ACCCGCCAAC ACCCGCTGAC GCGCCCTGAC GGGCTTGTCT GCTCCCGGCA TCCGCTTACA

7441 GACAAGCTGT GACCGTCTCC GGGAGCTGCA TGTGTCAGAG GTTTTCACCG TCATCACCGA

7501 AACGCGCGAG GCAGGGTGCC TTGATGTGGG CGCCGGCGGT CGAGTGGCGA CGGCGCGGCT

7561 TGTCCGCGCC CTGGTAGATT GCCTGGCCGT AGGCCAGCCA TTTTTGAGCG GCCAGCGGCC

7621 GCGATAGGCC GACGCGAAGC GGCGGGGCGT AGGGAGCGCA GCGACCGAAG GGTAGGCGCT

7681 TTTTGCAGCT CTTCGGCTGT GCGCTGGCCA GACAGTTATG CACAGGCCAG GCGGGTTTTA

7741 AGAGTTTTAA TAAGTTTTAA AGAGTTTTAG GCGGAAAAAT CGCCTTTTTT CTCTTTTATA

7801 TCAGTCACTT ACATGTGTGA CCGGTTCCCA ATGTACGGCT TTGGGTTCCC AATGTACGGG

7861 TTCCGGTTCC CAATGTACGG CTTTGGGTTC CCAATGTACG TGCTATCCAC AGGAAAGAGA

7921 CCTTTTCGAC CTTTTTCCCC TGCTAGGGCA ATTTGCCCTA GCATCTGCTC CGTACATTAG

7981 GAACCGGCGG ATGCTTCGCC CTCGATCAGG TTGCGGTAGC GCATGACTAG GATCGGGCCA

8041 GCCTGCCCCG CCTCCTCCTT CAAATCGTAC TCCGGCAGGT CATTTGACCC GATCAGCTTG

8101 CGCACGGTGA AACAGAACTT CTTGAACTCT CCGGCGCTGC CACTGCGTTC GTAGATCGTC

8161 TTGAACAACC ATCTGGCTTC TGCCTTGCCT GCGGCGCGGC GTGCCAGGCG GTAGAGAAAA

8221 CGGCCGATGC CGGGATCGAT CAAAAAGTAA TCGGGGTGAA CCGTCAGCAC GTCCGGGTTC

8281 TTGCCTTCTG TGATCTCGCG GTACATCCAA TCAGCTAGCT CGATCTCGAT GTACTCCGGC

8341 CGCCCGGTTT CGCTCTTTAC GATCTTGTAG CGGCTAATCA AGGCTTCACC CTCGGATACC

8401 GTCACCAGGC GGCCGTTCTT GGCCTTCTTC GTACGCTGCA TGGCAACGTG CGTGGTGTTT

8461 AACCGAATGC AGGTTTCTAC CAGGTCGTCT TTCTGCTTTC CGCCATCGGC TCGCCGGCAG

8521 AACTTGAGTA CGTCCGCAAC GTGTGGACGG AACACGCGGC CGGGCTTGTC TCCCTTCCCT

8581 TCCCGGTATC GGTTCATGGA TTCGGTTAGA TGGGAAACCG CCATCAGTAC CAGGTCGTAA

8641 TCCCACACAC TGGCCATGCC GGCCGGCCCT GCGGAAACCT CTACGTGCCC GTCTGGAAGC

8701 TCGTAGCGGA TCACCTCGCC AGCTCGTCGG TCACGCTTCG ACAGACGGAA AACGGCCACG

8761 TCCATGATGC TGCGACTATC GCGGGTGCCC ACGTCATAGA GCATCGGAAC GAAAAAATCT

8821 GGTTGCTCGT CGCCCTTGGG CGGCTTCCTA ATCGACGGCG CACCGGCTGC CGGCGGTTGC

8881 CGGGATTCTT TGCGGATTCG ATCAGCGGCC GCTTGCCACG ATTCACCGGG GCGTGCTTCT

8941 GCCTCGATGC GTTGCCGCTG GGCGGCCTGC GCGGCCTTCA ACTTCTCCAC CAGGTCATCA

9001 CCCAGCGCCG CGCCGATTTG TACCGGGCCG GATGGTTTGC GACCGTCACG CCGATTCCTC

9061 GGGCTTGGGG GTTCCAGTGC CATTGCAGGG CCGGCAGACA ACCCAGCCGC TTACGCCTGG

9121 CCAACCGCCC GTTCCTCCAC ACATGGGGCA TTCCACGGCG TCGGTGCCTG GTTGTTCTTG

9181 ATTTTCCATG CCGCCTCCTT TAGCCGCTAA AATTCATCTA CTCATTTATT CATTTGCTCA

9241 TTTACTCTGG TAGCTGCGCG ATGTATTCAG ATAGCAGCTC GGTAATGGTC TTGCCTTGGC

9301 GTACCGCGTA CATCTTCAGC TTGGTGTGAT CCTCCGCCGG CAACTGAAAG TTGACCCGCT

9361 TCATGGCTGG CGTGTCTGCC AGGCTGGCCA ACGTTGCAGC CTTGCTGCTG CGTGCGCTCG

9421 GACGGCCGGC ACTTAGCGTG TTTGTGCTTT TGCTCATTTT CTCTTTACCT CATTAACTCA

9481 AATGAGTTTT GATTTAATTT CAGCGGCCAG CGCCTGGACC TCGCGGGCAG CGTCGCCCTC

9541 GGGTTCTGAT TCAAGAACGG TTGTGCCGGC GGCGGCAGTG CCTGGGTAGC TCACGCGCTG

9601 CGTGATACGG GACTCAAGAA TGGGCAGCTC GTACCCGGCC AGCGCCTCGG CAACCTCACC

9661 GCCGATGCGC GTGCCTTTGA TCGCCCGCGA CACGACAAAG GCCGCTTGTA GCCTTCCATC

9721 CGTGACCTCA ATGCGCTGCT TAACCAGCTC CACCAGGTCG GCGGTGGCCC ATATGTCGTA

9781 AGGGCTTGGC TGCACCGGAA TCAGCACGAA GTCGGCTGCC TTGATCGCGG ACACAGCCAA

9841 GTCCGCCGCC TGGGGCGCTC CGTCGATCAC TACGAAGTCG CGCCGGCCGA TGGCCTTCAC

9901 GTCGCGGTCA ATCGTCGGGC GGTCGATGCC GACAACGGTT AGCGGTTGAT CTTCCCGCAC

9961 GGCCGCCCAA TCGCGGGCAC TGCCCTGGGG ATCGGAATCG ACTAACAGAA CATCGGCCCC

10021 GGCGAGTTGC AGGGCGCGGG CTAGATGGGT TGCGATGGTC GTCTTGCCTG ACCCGCCTTT

10081 CTGGTTAAGT ACAGCGATAA CCTTCATGCG TTCCCCTTGC GTATTTGTTT ATTTACTCAT

10141 CGCATCATAT ACGCAGCGAC CGCATGACGC AAGCTGTTTT ACTCAAATAC ACATCACCTT

10201 TTTAGACGGC GGCGCTCGGT TTCTTCAGCG GCCAAGCTGG CCGGCCAGGC CGCCAGCTTG

Supplemental Figure S10. F-SHMT08cΔ+F284A-CDS, insert size, vector, restriction enzymes, and sequenced positive clones used for cloning.

```
10261 GCATCAGACA AACCGGCCAG GATTTCATGC AGCCGCACGG TTGAGACGTG CGCGGGCGGC
10321 TCGAACACGT ACCCGGCCGC GATCATCTCC GCCTCGATCT CTTCGGTAAT GAAAAACGGT
10381 TCGTCCTGGC CGTCCTGGTG CGGTTTCATG CTTGTTCCTC TTGGCGTTCA TTCTCGGCGG
10441 CCGCCAGGGC GTCGGCCTCG GTCAATGCGT CCTCACGGAA GGCACCGCGC CGCCTGGCCT
10501 CGGTGGGCGT CACTTCCTCG CTGCGCTCAA GTGCGCGGTA CAGGGTCGAG CGATGCACGC
10561 CAAGCAGTGC AGCCGCCTCT TTCACGGTGC GGCCTTCCTG GTCGATCAGC TCGCGGGCGT
10621 GCGCGATCTG TGCCGGGGTG AGGGTAGGGC GGGGGCCAAA CTTCACGCCT CGGGCCTTGG
10681 CGGCCTCGCG CCCGCTCCGG GTGCGGTCGA TGATTAGGGA ACGCTCGAAC TCGGCAATGC
10741 CGGCGAACAC GGTCAACACC ATGCGGCCGG CCGGCGTGGT GGTGTCGGCC CACGGCTCTG
10801 CCAGGCTACG CAGGCCCGCG CCGGCCTCCT GGATGCGCTC GGCAATGTCC AGTAGGTCGC
10861 GGGTGCTGCG GGCCAGGCGG TCTAGCCTGG TCACTGTCAC AACGTCGCCA GGGCGTAGGT
10921 GGTCAAGCAT CCTGGCCAGC TCCGGGCGGT CGCGCCTGGT GCCGGTGATC TTCTCGGAAA
10981 ACAGCTTGGT GCAGCCGGCC GCGTGCAGTT CGGCCCGTTG GTTGGTCAAG TCCTGGTCGT
11041 CGGTGCTGAC GCGGGCATAG CCCAGCAGGC CAGCGGCGGC GCTCTTGTTC ATGGCGTAAT
11101 GTCTCCGGTT CTAGTCGCAA GTATTCTACT TTATGCGACT AAAACACGCG ACAAGAAAAC
11161 GCCAGGAAAA GGGCAGGGCG GCAGCCTGTC GCGTAACTTA GGACTTGTGC GACATGTCGT
11221 TTTCAGAAGA CGGCTGCACT GAACGTCAGA AGCCGACTGC ACTATAGCAG CGGAGGGGTT
11281 GGATCAAAGT ACT
```

Supplemental Figure S10. F-SHMT08cΔ+F284A-CDS, insert size, vector, restriction enzymes, and sequenced positive clones used for cloning.

Gene Name F-SHMT08cΔ+N374A-CDS
Cloning Vector pG2RNAi2
Length (bp) 1430
Cloning Sites AscI-AvrII

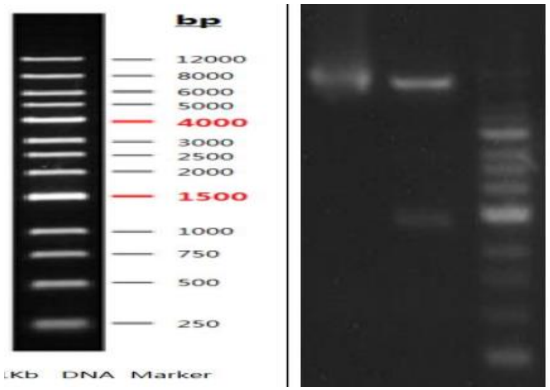

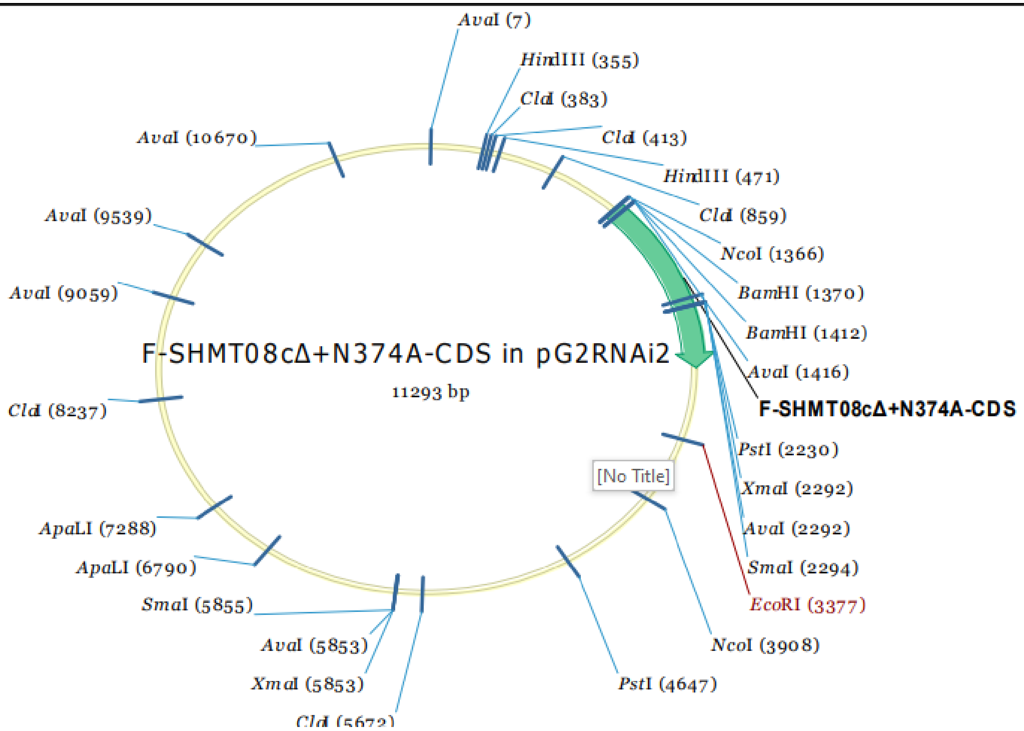

Supplemental Figure S11. F-SHMT08cΔ+N374A-CDS, insert size, vector, restriction enzymes, and sequenced positive clones used for cloning.

Detailed Sequence of the Whole Construct:

```
  1 TTGATCCCGA GGGGAACCCT GTGGTTGGCA TGCACATACA AATGGACGAA CGGATAAACC

 61 TTTTCACGCC CTTTTAAATA TCCGTTATTC TAATAAACGC TCTTTTCTCT TAGGTTTACC

121 CGCCAATATA TCCTGTCAAA CACTGATAGT TTAAACTGAA GGCGGGAAAC GACAATCTGA

181 TCCAAGCTCA AGCTCATTCG CCATTCAGGC TGCGCAACTG TTGGGAAGGG CGATCGGTGC

241 GGGCCTCTTC GCTATTACGC CAGCTGGCGA AAGGGGGATG TGCTGCAAGG CGATTAAGTT

301 GGGTAACGCC AGGGTTTTCC CAGTCACGAC GTTGTAAAAC GACGGCCAGT GCCAAGCTTA

361 TTACCCTGTT ATCCCTAGAT ATCGATTTTG GCTACCTTAA GGTAGCCAAA ATCGATCACT

421 AGTGCGGCCG CGGGCCCAAT ATAACAACGA CGTCGTAACA GATAAAGCGA AGCTTGAAGG

481 TGCATGTGAC TCCGTCAAGA TTACGAAACC GCCAACTACC ACGCAAATTG CAATTCTCAA

541 TTTCCTAGAA GGACTCTCCG AAAATGCATC CAATACCAAA TATTACCCGT GTCATAGGCA

601 CCAAGTGACA CCATACATGA ACACGCGTCA CAATATGACT GGAGAAGGGT TCCACACCTT

661 ATGCTATAAA ACGCCCCACA CCCCTCCTCC TTCCTTCGCA GTTCAATTCC AATATATTCC

721 ATTCTCTCTG TGTATTTCCC TACCTCTCCC TTCAAGGTTA GTCGATTTCT TCTGTTTTTC

781 TTCTTCGTTC TTTCCATGAA TTGTGTATGT TCTTTGATCA ATACGATGTT GATTTGATTG

841 TGTTTTGTTT GGTTTCATCG ATCTTCAATT TTCATAATCA GATTCAGCTT TTATTATCTT
```

Supplemental Figure S11. F-SHMT08cΔ+N374A-CDS, insert size, vector, restriction enzymes, and sequenced positive clones used for cloning.

```
901 TACAACAACG TCCTTAATTT GATGATTCTT TAATCGTAGA TTTGCTCTAA TTAGAGCTTT
961 TTCATGTCAG ATCCCTTTAC AACAAGCCTT AATTGTTGAT TCATTAATCG TAGATTAGGG
1021 CTTTTTTCAT TGATTACTTC AGATCCGTTA AACGTAACCA TAGATCAGGG CTTTTTCATG
1081 AATTACTTCA GATCCGTTAA ACAACAGCCT TATTTTTTAT ACTTCTGTGG TTTTTCAAGA
1141 AATTGTTCAG ATCCGTTGAC AAAAAGCCTT ATTCGTTGAT TCTATATCGT TTTTCGAGAG
1201 ATATTGCTCA GATCTGTTAG CAACTGCCTT GTTTGTTGAT TCTATTGCCG TGGATTAGGG
1261 TTTTTTTTCA CGAGATTGCT TCAGATCCGT ACTTAAGATT ACGTAATGGA TTTTGATTCT
1321 GATTTATCTG TGATTGTTGA CTCGACAGCG GCCGCACCGG CGCGCCATGG ATCCAGTAAG
1381 CGTGTGGGGT AACACGCCCT TGGCGACGGT GGATCCCGAG ATCCATGACC TCATCGAGAA
1441 GGAGAAGCGC CGTCAATGCC GCGGAATCGA GCTCATCGCC TCCGAGAACT TCACCTCCTT
1501 CGCCGTCATC GAGGCCCTCG GCAGCGCTCT CACGAACAAA TACTCCGAGG GCATGCCGGG
1561 CAACCGCTAC TACGGCGGCA ATGAATACAT CGACCAGATC GAAAACCTCT GCCGCTCACG
1621 CGCCCTCCAA GCCTTCCACC TCGACGCCCA ATCCTGGGGC GTCAACGTCC AGCCCTACTC
1681 CGGCTCCCCG GCCAACTTCG CCGCCTACAC CGCCGTCCTC AACCCCCACG ACCGCATCAT
1741 GGGGCTAGAT CTCCGCTCCG GCGGCCACCT CACCCACGGC TACTACACCT CCGGCGGAAA
1801 GAAGATCTCC GCCACCTCCA TTTACTTCGA GAGTCTCCCT TACAAGGTAA ACTCCACCAC
1861 CGGCTACATC GACTACGACC GCTTGGAAGA AAAAGCCCTA GACTTCAGGC CAAAACTCAT
1921 AATCTGCGGT GGCAGCGCGT ACCCTCGCGA TTGGGACTAC AAACGTTTCA GGGAAGTCGC
1981 TGATAAGTGC GGAGCATTGC TTCTCTGCGA CATGGCGCAC ACTAGCGGCC TTGTGGCCGC
2041 GCAGGAAGTG AACAGCCCCT TCGAGTATTG CGACATTGTG ACCACCACGA CTCACAAGAG
2101 CTTGCGGGGC CCACGTGCGG GGATGATCTT TTACCGGAAG GGCCCCAAGC CGCCGAAGAA
2161 GGGGCAGCCG GAGAACGCGG TTTATGATTT CGAGGACAAG ATTAACTTCG CGGTGTTCCC
2221 TTCGCTGCAG GGTGGGCCCC ACAACCACCA GATCGGTGCT CTCGCCGTGG CGCTGAAGCA
2281 GGCCGCGTCG CCCGGGTTTA AGGCCTACGC GAAGCAGGTT AAGGCGAACG CCGTTGCGCT
2341 TGGAAAATAC TTGATGGGGA AAGGGTACAG CCTTGTCACT GGCGGAACGG AGAACCATCT
2401 TGTTTTGTGG GATCTGAGAC CTCTTGGATT GACTGGGTAT AAGGTGGAGA AACTCTGTGA
2461 TCTCTGTAAC ATTACTGTTA ACAAGGCTGC TGTTTTTGGT GATAGCAGTG CCTTGGCCCC
2521 TGGTGGAGTG CGAATTGGTG CCCCTGCCAT GACTTCTAGG GGTTTGGTTG AAAAAGACTT
2581 TGAGCAGATT GGTGAGTTCC TTCACCGTGC TGTGACTCTC ACACTGGAGA TCCAGAAGGA
2641 GCATGGCAAA CTTCTCAAGG ATTTCAACAA GGGTCTCGTC AACAACAAGG CTATTGAAGA
2701 TCTCAAAGCT GATGTTGAGA AGTTCTCTGC CTTGTTTGAC ATGCCTGGCT TCCTGGTATC
2761 TGAAATGAAG TACAAGGATT AGCCTAGGTT CGAGTATTAT GGCATTGGGA AAACTGTTTT
2821 TCTTGTACCA TTTGTTGTGC TTGTAATTTA CTGTGTTTTT TATTCGGTTT TCGCTATCGA
2881 ACTGTGAAAT GGAAATGGAT GGAGAAGAGT TAATGAATGA TATGGTCCTT TTGTTCATTC
2941 TCAAATTAAT ATTATTTGTT TTTTCTCTTA TTTGTTGTGT GTTGAATTTG AAATTATAAG
3001 AGATATGCAA ACATTTTGTT TTGAGTAAAA ATGTGTCAAA TCGTGGCCTC TAATGACCGA
3061 AGTTAATATG AGGAGTAAAA CACTTGTAGT TGTACCATTA TGCTTATTCA CTAGGCAACA
3121 AATATATTTT CAGACCTAGA AAAGCTGCAA ATGTTACTGA ATACAAGTAT GTCCTCTTGT
3181 GTTTTAGACA TTTATGAACT TTCCTTTATG TAATTTTCCA GAATCCTTGT CAGATTCTAA
3241 TCATTGCTTT ATAATTATAG TTATACTCAT GGATTTGTAG TTGAGTATGA AAATATTTTT
3301 TAATGCATTT TATGACTTGC CAATTGATTG ACAACATGCA TCAATCCGCG GTTATGACTC
3361 TCTTAAGAGA GTCATGAATT CGAGCTTCCA GAAGGTAATT ATCCAAGATG TAGCATCAAG
3421 AATCCAATGT TTACGGGAAA AACTATGGAA GTATTATGTG AGCTCAGCAA GAAGCAGATC
3481 AATATGCGGC ACATATGCAA CCTATGTTCA AAAATGAAGA ATGTACAGAT ACAAGATCCT
3541 ATACTGCCAG AATACGAAGA AGAATACGTA GAAATTGAAA AAGAAGAACC AGGCGAAGAA
3601 AAGAATCTTG AAGACGTAAG CACTGACGAC AACAATGAAA AGAAGAAGAT AAGGTCGGTG
3661 ATTGTGAAAG AGACATAGAG GACACATGTA AGGTGGAAAA TGTAAGGGCG GAAAGTAACC
3721 TTATCACAAA GGAATCTTAT CCCCCACTAC TTATCCTTTT ATATTTTTCC GTGTCATTTT
3781 TGCCCTTGAG TTTTCCTATA TAAGGAACCA AGTTCGGCAT TTGTGAAAAC AAGAAAAAAT
3841 TTGGTGTAAG CTATTTTCTT TGAAGTACTG AGGATACAAC TTCAGAGAAA TTTGTAAGTT
3901 TGTGATCCAT GGTGAGCAAG GGCGAGGAGC TGTTCACCGG GGTGGTGCCC ATCCTGGTCG
3961 AGCTGGACGG CGACGTAAAC GGCCACAAGT TCAGCGTGTC CGGCGAGGGC GAGGGCGATG
```

Supplemental Figure S11. F-SHMT08cΔ+N374A-CDS, insert size, vector, restriction enzymes, and sequenced positive clones used for cloning.

4021 CCACCTACGG CAAGCTGACC CTGAAGTTCA TCTGCACCAC CGGCAAGCTG CCCGTGCCCT
4081 GGCCCACCCT CGTGACCACC TTCACCTACG GCGTGCAGTG CTTCAGCCGC TACCCCGACC
4141 ACATGAAGCA GCACGACTTC TTCAAGTCCG CCATGCCCGA AGGCTACGTC CAGGAGCGCA
4201 CCATCTTCTT CAAGGACGAC GGCAACTACA AGACCCGCGC CGAGGTGAAG TTCGAGGGCG
4261 ACACCCTGGT GAACCGCATC GAGCTGAAGG GCATCGACTT CAAGGAGGAC GGCAACATCC
4321 TGGGGCACAA GCTGGAGTAC AACTACAACA GCCACAACGT CTATATCATG GCCGACAAGC
4381 AGAAGAACGG CATCAAGGTG AACTTCAAGA TCCGCCACAA CATCGAGGAC GGCAGCGTGC
4441 AGCTCGCCGA CCACTACCAG CAGAACACCC CCATCGGCGA CGGCCCCGTG CTGCTGCCCG
4501 ACAACCACTA CCTGAGCACC CAGTCCGCCC TGAGCAAAGA CCCCAACGAG AAGCGCGATC
4561 ACATGGTCCT GCTGGAGTTC GTGACCGCCG CCGGGATCAC TCACGGCATG GACGAGCTGT
4621 ACAAGTAAAG CGGCCGCCCG GCTGCAGATC GTTCAAACAT TTGGCAATAA AGTTTCTTAA
4681 GATTGAATCC TGTTGCCGGT CTTGCGATGA TTATCATATA ATTTCTGTTG AATTACGTTA
4741 AGCATGTAAT AATTAACATG TAATGCATGA CGTTATTTAT GAGATGGGTT TTTATGATTA
4801 GAGTCCCGCA ATTATACATT TAATACGCGA TAGAAAACAA AATATAGCGC GCAAACTAGG
4861 ATAAATTATC GCGCGCGGTG TCATCTATGT TACTAGATCC GATGATAAGC TGTCAAACAT
4921 GAGAATTAAT TCGTAATCAT GTCATAGCTG TTTCCTGTGT GAAATTGTTA TCCGCTCACA
4981 ATTCCACACA ACATACGAGC CGGAAGCATA AAGTGTAAAG CCTGGGGTGC CTAATGAGTG
5041 AGCTAACTCA CATTAATTGC GTTGCGCTCA CTGCCCGCTT TCCAGTCGGG AAACCTGTCG
5101 TGCCAGCTGC ATTAATGAAT CGGCCAACGC GCGGGGAGAG GCGGTTTGCG TATTGGCTAG
5161 AGCAATTCGG CGTTAATTCA GTACATTAAA AACGTCCGCA ATGTGTTATT AAGTTGTCTA
5221 AGCGTCAATT TGTTTACACC ACAATATATC CTGCCACCAG CCAGCCAACA GCTCCCCGAC
5281 CGGCAGCTCG GCACAAAATC ACCACTCGAT ACAGGCAGCC CATCAGTCCG GGACGGCGTC
5341 AGCGGGAGAG CCGTTGTAAG GCGGCAGACT TTGCTCATGT TACCGATGCT ATTCGGAAGA
5401 ACGGCAACTA AGCTGCCGGG TTTGAAACAC GGATGATCTC GCGGAGGGTA GCATGTTGAT
5461 TGTAACGATG ACAGAGCGTT GCTGCCTGTG ATCCAGATCA TGAACAATAA AACTGTCTGC
5521 TTACATAAAC AGTAATACAA GGGGTGTTAT GAGCCATATT CAACGGGAAA CGTCTTGCTC
5581 TAGGCCGCGA TTAAATTCCA ACATGGATGC TGATTTATAT GGGTATAAAT GGGCTCGCGA
5641 TAATGTCGGG CAATCAGGTG CGACAATCTA TCGATTGTAT GGGAAGCCCG ATGCGCCAGA
5701 GTTGTTTCTG AAACATGGCA AAGGTAGCGT TGCCAATGAT GTTACAGATG AGATGGTCAG
5761 ACTAAACTGG CTGACGGAAT TTATGCCTCT TCCGACCATC AAGCATTTTA TCCGTACTCC
5821 TGATGATGCA TGGTTACTCA CCACTGCGAT CCCCGGGAAA ACAGCATTCC AGGTATTAGA
5881 AGAATATCCT GATTCAGGTG AAAATATTGT TGATGCGCTG GCAGTGTTCC TGCGCCGGTT
5941 GCATTCGATT CCTGTTTGTA ATTGTCCTTT TAACAGCGAT CGCGTATTTC GTCTCGCTCA
6001 GGCGCAATCA CGAATGAATA ACGGTTTGGT TGATGCGAGT GATTTTGATG ACGAGCGTAA
6061 TGGCTGGCCT GTTGAACAAG TCTGGAAAGA AATGCATAAA CTTTTGCCAT TCTCACCGGA
6121 TTCAGTCGTC ACTCATGGTG ATTTCTCACT TGATAACCTT ATTTTTGACG AGGGGAAATT
6181 AATAGGTTGT ATTGATGTTG GACGAGTCGG AATCGCAGAC CGATACCAGG ATCTTGCCAT
6241 CCTATGGAAC TGCCTCGGTG AGTTTTCTCC TTCATTACAG AAACGGCTTT TTCAAAAATA
6301 TGGTATTGAT AATCCTGATA TGAATAAATT GCAGTTTCAT TTGATGCTCG ATGAGTTTTT
6361 CTAAGAATTA ATTCATGATC CTGCATGACC AAAATCCCTT AACGTGAGTT TTCGTTCCAC
6421 TGAGCGTCAG ACCCCGTAGA AAAGATCAAA GGATCTTCTT GAGATCCTTT TTTTCTGCGC
6481 GTAATCTGCT GCTTGCAAAC AAAAAAACCA CCGCTACCAG CGGTGGTTTG TTTGCCGGAT
6541 CAAGAGCTAC CAACTCTTTT TCCGAAGGTA ACTGGCTTCA GCAGAGCGCA GATACCAAAT
6601 ACTGTCCTTC TAGTGTAGCC GTAGTTAGGC CACCACTTCA AGAACTCTGT AGCACCGCCT
6661 ACATACCTCG CTCTGCTAAT CCTGTTACCA GTGGCTGCTG CCAGTGGCGA TAAGTCGTGT
6721 CTTACCGGGT TGGACTCAAG ACGATAGTTA CCGGATAAGG CGCAGCGGTC GGGCTGAACG
6781 GGGGGTTCGT GCACACAGCC CAGCTTGGAG CGAACGACCT ACACCGAACT GAGATACCTA
6841 CAGCGTGAGC TATGAGAAAG CGCCACGCTT CCCGAAGGGA GAAAGGCGGA CAGGTATCCG
6901 GTAAGCGGCA GGGTCGGAAC AGGAGAGCGC ACGAGGGAGC TTCCAGGGGG AAACGCCTGG
6961 TATCTTTATA GTCCTGTCGG GTTTCGCCAC CTCTGACTTG AGCGTCGATT TTTGTGATGC
7021 TCGTCAGGGG GGCGGAGCCT ATGGAAAAAC GCCAGCAACG CGGCCTTTTT ACGGTTCCTG
7081 GCCTTTTGCT GGCCTTTTGC TCACATGTTC TTTCCTGCGT TATCCCCTGA TTCTGTGGAT

Supplemental Figure S11. F-SHMT08cΔ+N374A-CDS, insert size, vector, restriction enzymes, and sequenced positive clones used for cloning.

7141 AACCGTATTA CCGCCTTTGA GTGAGCTGAT ACCGCTCGCC GCAGCCGAAC GACCGAGCGC

7201 AGCGAGTCAG TGAGCGAGGA AGCGGAAGAG CGCCTGATGC GGTATTTTCT CCTTACGCAT

7261 CTGTGCGGTA TTTCACACCG CATATGGTGC ACTCTCAGTA CAATCTGCTC TGATGCCGCA

7321 TAGTTAAGCC AGTATACACT CCGCTATCGC TACGTGACTG GGTCATGGCT GCGCCCCGAC

7381 ACCCGCCAAC ACCCGCTGAC GCGCCCTGAC GGGCTTGTCT GCTCCCGGCA TCCGCTTACA

7441 GACAAGCTGT GACCGTCTCC GGGAGCTGCA TGTGTCAGAG GTTTTCACCG TCATCACCGA

7501 AACGCGCGAG GCAGGGTGCC TTGATGTGGG CGCCGGCGGT CGAGTGGCGA CGGCGCGGCT

7561 TGTCCGCGCC CTGGTAGATT GCCTGGCCGT AGGCCAGCCA TTTTTGAGCG GCCAGCGGCC

7621 GCGATAGGCC GACGCGAAGC GGCGGGGCGT AGGGAGCGCA GCGACCGAAG GGTAGGCGCT

7681 TTTTGCAGCT CTTCGGCTGT GCGCTGGCCA GACAGTTATG CACAGGCCAG GCGGGTTTTA

7741 AGAGTTTTAA TAAGTTTTAA AGAGTTTTAG GCGGAAAAAT CGCCTTTTTT CTCTTTTATA

7801 TCAGTCACTT ACATGTGTGA CCGGTTCCCA ATGTACGGCT TTGGGTTCCC AATGTACGGG

7861 TTCCGGTTCC CAATGTACGG CTTTGGGTTC CCAATGTACG TGCTATCCAC AGGAAAGAGA

7921 CCTTTTCGAC CTTTTTCCCC TGCTAGGGCA ATTTGCCCTA GCATCTGCTC CGTACATTAG

7981 GAACCGGCGG ATGCTTCGCC CTCGATCAGG TTGCGGTAGC GCATGACTAG GATCGGGCCA

8041 GCCTGCCCCG CCTCCTCCTT CAAATCGTAC TCCGGCAGGT CATTTGACCC GATCAGCTTG

8101 CGCACGGTGA AACAGAACTT CTTGAACTCT CCGGCGCTGC CACTGCGTTC GTAGATCGTC

8161 TTGAACAACC ATCTGGCTTC TGCCTTGCCT GCGGCGCGGC GTGCCAGGCG GTAGAGAAAA

8221 CGGCCGATGC CGGGATCGAT CAAAAAGTAA TCGGGGTGAA CCGTCAGCAC GTCCGGGTTC

8281 TTGCCTTCTG TGATCTCGCG GTACATCCAA TCAGCTAGCT CGATCTCGAT GTACTCCGGC

8341 CGCCCGGTTT CGCTCTTTAC GATCTTGTAG CGGCTAATCA AGGCTTCACC CTCGGATACC

8401 GTCACCAGGC GGCCGTTCTT GGCCTTCTTC GTACGCTGCA TGGCAACGTG CGTGGTGTTT

8461 AACCGAATGC AGGTTTCTAC CAGGTCGTCT TTCTGCTTTC CGCCATCGGC TCGCCGGCAG

8521 AACTTGAGTA CGTCCGCAAC GTGTGGACGG AACACGCGGC CGGGCTTGTC TCCCTTCCCT

8581 TCCCGGTATC GGTTCATGGA TTCGGTTAGA TGGGAAACCG CCATCAGTAC CAGGTCGTAA

8641 TCCCACACAC TGGCCATGCC GGCCGGCCCT GCGGAAACCT CTACGTGCCC GTCTGGAAGC

8701 TCGTAGCGGA TCACCTCGCC AGCTCGTCGG TCACGCTTCG ACAGACGGAA AACGGCCACG

8761 TCCATGATGC TGCGACTATC GCGGGTGCCC ACGTCATAGA GCATCGGAAC GAAAAAATCT

8821 GGTTGCTCGT CGCCCTTGGG CGGCTTCCTA ATCGACGGCG CACCGGCTGC CGGCGGTTGC

8881 CGGGATTCTT TGCGGATTCG ATCAGCGGCC GCTTGCCACG ATTCACCGGG GCGTGCTTCT

8941 GCCTCGATGC GTTGCCGCTG GGCGGCCTGC GCGGCCTTCA ACTTCTCCAC CAGGTCATCA

9001 CCCAGCGCCG CGCCGATTTG TACCGGGCCG GATGGTTTGC GACCGTCACG CCGATTCCTC

9061 GGGCTTGGGG GTTCCAGTGC CATTGCAGGG CCGGCAGACA ACCCAGCCGC TTACGCCTGG

9121 CCAACCGCCC GTTCCTCCAC ACATGGGGCA TTCCACGGCG TCGGTGCCTG GTTGTTCTTG

9181 ATTTTCCATG CCGCCTCCTT TAGCCGCTAA AATTCATCTA CTCATTTATT CATTTGCTCA

9241 TTTACTCTGG TAGCTGCGCG ATGTATTCAG ATAGCAGCTC GGTAATGGTC TTGCCTTGGC

9301 GTACCGCGTA CATCTTCAGC TTGGTGTGAT CCTCCGCCGG CAACTGAAAG TTGACCCGCT

9361 TCATGGCTGG CGTGTCTGCC AGGCTGGCCA ACGTTGCAGC CTTGCTGCTG CGTGCGCTCG

9421 GACGGCCGGC ACTTAGCGTG TTTGTGCTTT TGCTCATTTT CTCTTTACCT CATTAACTCA

9481 AATGAGTTTT GATTTAATTT CAGCGGCCAG CGCCTGGACC TCGCGGGCAG CGTCGCCCTC

9541 GGGTTCTGAT TCAAGAACGG TTGTGCCGGC GGCGGCAGTG CCTGGGTAGC TCACGCGCTG

9601 CGTGATACGG GACTCAAGAA TGGGCAGCTC GTACCCGGCC AGCGCCTCGG CAACCTCACC

9661 GCCGATGCGC GTGCCTTTGA TCGCCCGCGA CACGACAAAG GCCGCTTGTA GCCTTCCATC

9721 CGTGACCTCA ATGCGCTGCT TAACCAGCTC CACCAGGTCG GCGGTGGCCC ATATGTCGTA

9781 AGGGCTTGGC TGCACCGGAA TCAGCACGAA GTCGGCTGCC TTGATCGCGG ACACAGCCAA

9841 GTCCGCCGCC TGGGGCGCTC CGTCGATCAC TACGAAGTCG CGCCGGCCGA TGGCCTTCAC

9901 GTCGCGGTCA ATCGTCGGGC GGTCGATGCC GACAACGGTT AGCGGTTGAT CTTCCCGCAC

9961 GGCCGCCCAA TCGCGGGCAC TGCCCTGGGG ATCGGAATCG ACTAACAGAA CATCGGCCCC

10021 GGCGAGTTGC AGGGCGCGGG CTAGATGGGT TGCGATGGTC GTCTTGCCTG ACCCGCCTTT

10081 CTGGTTAAGT ACAGCGATAA CCTTCATGCG TTCCCCTTGC GTATTTGTTT ATTTACTCAT

10141 CGCATCATAT ACGCAGCGAC CGCATGACGC AAGCTGTTTT ACTCAAATAC ACATCACCTT

10201 TTTAGACGGC GGCGCTCGGT TTCTTCAGCG GCCAAGCTGG CCGGCCAGGC CGCCAGCTTG

Supplemental Figure S11. F-SHMT08cΔ+N374A-CDS, insert size, vector, restriction enzymes, and sequenced positive clones used for cloning.

10261 GCATCAGACA AACCGGCCAG GATTTCATGC AGCCGCACGG TTGAGACGTG CGCGGGCGGC

10321 TCGAACACGT ACCCGGCCGC GATCATCTCC GCCTCGATCT CTTCGGTAAT GAAAAACGGT

10381 TCGTCCTGGC CGTCCTGGTG CGGTTTCATG CTTGTTCCTC TTGGCGTTCA TTCTCGGCGG

10441 CCGCCAGGGC GTCGGCCTCG GTCAATGCGT CCTCACGGAA GGCACCGCGC CGCCTGGCCT

10501 CGGTGGGCGT CACTTCCTCG CTGCGCTCAA GTGCGCGGTA CAGGGTCGAG CGATGCACGC

10561 CAAGCAGTGC AGCCGCCTCT TTCACGGTGC GGCCTTCCTG GTCGATCAGC TCGCGGGCGT

10621 GCGCGATCTG TGCCGGGGTG AGGGTAGGGC GGGGGCCAAA CTTCACGCCT CGGGCCTTGG

10681 CGGCCTCGCG CCCGCTCCGG GTGCGGTCGA TGATTAGGGA ACGCTCGAAC TCGGCAATGC

10741 CGGCGAACAC GGTCAACACC ATGCGGCCGG CCGGCGTGGT GGTGTCGGCC CACGGCTCTG

10801 CCAGGCTACG CAGGCCCGCG CCGGCCTCCT GGATGCGCTC GGCAATGTCC AGTAGGTCGC

10861 GGGTGCTGCG GGCCAGGCGG TCTAGCCTGG TCACTGTCAC AACGTCGCCA GGGCGTAGGT

10921 GGTCAAGCAT CCTGGCCAGC TCCGGGCGGT CGCGCCTGGT GCCGGTGATC TTCTCGGAAA

10981 ACAGCTTGGT GCAGCCGGCC GCGTGCAGTT CGGCCCGTTG GTTGGTCAAG TCCTGGTCGT

11041 CGGTGCTGAC GCGGGCATAG CCCAGCAGGC CAGCGGCGGC GCTCTTGTTC ATGGCGTAAT

11101 GTCTCCGGTT CTAGTCGCAA GTATTCTACT TTATGCGACT AAAACACGCG ACAAGAAAAC

11161 GCCAGGAAAA GGGCAGGGCG GCAGCCTGTC GCGTAACTTA GGACTTGTGC GACATGTCGT

11221 TTTCAGAAGA CGGCTGCACT GAACGTCAGA AGCCGACTGC ACTATAGCAG CGGAGGGGTT

11281 GGATCAAAGT ACT

Supplemental Figure S11. F-SHMT08cΔ+N374A-CDS, insert size, vector, restriction enzymes, and sequenced positive clones used for cloning.
